# Polymorphism analysis of six selenoprotein genes: support for a selective sweep at the glutathione peroxidase 1 locus (3p21) in Asian populations

**DOI:** 10.1186/1471-2156-7-56

**Published:** 2006-12-11

**Authors:** Charles B Foster, Kshama Aswath, Stephen J Chanock, Heather F McKay, Ulrike Peters

**Affiliations:** 1 Section of Pediatric Infectious Diseases, Division of Pediatrics, Desk A120, The Children's Hospital, The Cleveland Clinic, 9500 Euclid Avenue, Cleveland, OH 44195, USA; 2 Division of Pediatric Infectious Diseases, Department of Pediatrics, Johns Hopkins University, 600 North Wolfe Street, Park 256, Baltimore, MD, 21287, USA; 3 Section of Genomic Variation, Pediatric Oncology Branch, National Cancer Institute, National Institutes of Health, Bethesda, MD, 20892, USA; 4 Core Genotyping Facility, Advanced Technology Center, National Cancer Institute, Bethesda, MD, 20892, USA; 5 Division of Cancer Epidemiology and Genetics, National Cancer Institute, NIH, Department of Health and Human Services, Rockville, Maryland, USA; 6 Cancer Prevention Program, Fred Hutchinson Cancer Research Center, Seattle, WA, 98109, USA

## Abstract

**Background:**

There are at least 25 human selenoproteins, each characterized by the incorporation of selenium into the primary sequence as the amino acid selenocysteine. Since many selenoproteins have antioxidant properties, it is plausible that inter-individual differences in selenoprotein expression or activity could influence risk for a range of complex diseases, such as cancer, infectious diseases as well as deleterious responses to oxidative stressors like cigarette smoke. To capture the common genetic variants for 6 important selenoprotein genes (*GPX1*, *GPX2*, *GPX3*, *GPX4*, *TXNRD1*, and *SEPP1*) known to contribute to antioxidant host defenses, a re-sequence analysis was conducted across these genes with particular interest directed at the coding regions, intron-exon borders and flanking untranslated regions (UTR) for each gene in an 102 individual population representative of 4 major ethnic groups found within the United States.

**Results:**

For 5 of the genes there was no strong evidence for selection according to the expectations of the neutral equilibrium model of evolution; however, at the *GPX1 *locus (3p21) there was evidence for positive selection. Strong confirmatory evidence for recent positive selection at the genomic region 3p21 in Asian populations is provided by data from the International HapMap project.

**Conclusion:**

The SNPs and fine haplotype maps described in this report will be valuable resources for future functional studies, for population specific genetic studies designed to comprehensively explore the role of selenoprotein genetic variants in the etiology of various human diseases, and to define the forces responsible for a recent selective sweep in the vicinity of the *GPX1 *locus.

## Background

Increasing data suggests that selenium deficiency is a risk factor for certain cancers, neurodegenerative disorders and complications from diabetes [1-4]. Selenium is required for normal immune function and selenium deficiency can be associated with enhanced infectious disease severity [1,5]. Selenium deficiency impairs the expression and production of selenium containing enzymes, known as selenoproteins, resulting in enhanced susceptibility to oxidative stress. In addition, it is possible that functional polymorphisms in selenoprotein genes might also influence selenoenzyme expression, stability or activity modifying disease outcomes in a manner similar to that observed with selenium deficiency.

The 6 genes selected for re-sequencing in this project play an important role in antioxidant defense; they include selenoprotein P (*SEPP1*), thioredoxin reductase 1 (*TXNRD1*), and 4 selenium containing glutathione peroxidase genes, *GPX1*, *GPX2*, *GPX3 *and *GPX4 *[6-8]. The glutathione peroxidase family is the largest of the selenoprotein gene families. Glutathione peroxidases are named for the ability to use glutathione as a reducing substrate. *GPX1 *and *GPX2 *appear to have similar substrate specificity, catalyzing the reduction of hydrogen peroxide to water, but differ in their tissue distribution, with *GPX1 *expression being particularly abundant in erythrocytes and *GPX2 *expression being restricted primarily to the gastrointestinal tract [9,10]. GPX1 knockout mice have a normal phenotype, but are highly sensitive to oxidative stressors[11]. Some epidemiologic studies have correlated low GPX1 activity or particular *GPX1 *polymorphisms with enhanced risk of cancer, although these correlations have not been consistently observed in all populations [12-17]. Mice with combined disruption of *GPX1 *and *GPX2 *develop bacteria associated ileocolitis and intestinal cancers [9]. *GPX3 *(extracellular or plasma) is a circulating plasma selenoprotein and is able to utilize thioredoxin reductase, thioredoxin or glutaredoxin as reductants [18]. GPX4 reduces phospholipid hydroperoxides, localizes to the mitochondria or to the nucleus and the cytosol, and appears to be essential for survival [19,20]. *GPX4 *expression is particularly high in various endocrine tissues, especially the testis. Moreover, in mature spermatozoa, GPX4 functions as a structural protein that helps anchor the helix of mitochondria in the midpiece of spermatozoa, suggesting a possible mechanism by which selenium deficiency might impair fertility [21,22]. *SEPP1 *is a major plasma selenoprotein and along with *GPX3 *accounts for the majority of plasma selenium[23]. *SEPP1 *is a secreted protein that likely functions as a selenium delivery molecule and perhaps as an extracellular antioxidant with glutathione peroxidase-like activity [24]. Unique among the selenoproteins, *SEPP1 *has 10 in frame UGA codons, each encoding for the selenium containing amino acid selenocysteine [25]; the other known selenoproteins generally have only one UGA codon [26]. Cytosolic thioredoxin reductase (*TXNRD1*) is one of the most abundant selenium-containing proteins and is able to catalyze the reduction of thioredoxin in a reaction that uses electrons from NADPH [27]. TXNRD1 is a major antioxidant redox regulator and supports the function of p53. It's expression may be regulated in a contrasting pattern to *GPX1 *in certain cancer systems and disruption of its expression may reverse the phenotype and carcinogenicity of lung cancer cells [28].

The primary goal of this study was to characterize genetic variation across 6 selenoprotein genes. Specifically, re-sequence analysis was performed in a multiethnic population to determine common single nucleotide polymorphisms (SNPs) and estimate haplotypes for use in large genetic association studies or for future functional studies. Sequence analysis targeted exons, regulatory regions and the sequence motifs characteristic of selenoproteins; the latter include an in frame UGA "stop" codon that is recoded to allow insertion of the selenium containing amino acid selenocysteine [26]. Both cis-acting features, including a 3' UTR RNA stem loop known as a selenocysteine insertion sequence (SECIS), and trans-acting factors (including tRNA-selenocysteine (*TRSP*), a selenocysteine-tRNA-specific elongation factor (*EEFSEC*) and SECIS binding protein 2 (*SECISBP2*)) are required for efficient selenoprotein translation [29-32]. Lastly, the selenoprotein SNPs and fine haplotype maps described in this report will be valuable resources for future functional studies and for population specific genetic studies designed to comprehensively explore the role of selenoprotein genetic variants in the etiology of human diseases.

## Results

### Polymorphism analysis

Six selenoprotein genes (*GPX1*, *GPX2*, *GPX3*, *GPX4*, *SEPP1 *and *TXNRD1*) were re-sequenced using the SNP500 polymorphism discovery resource (Table 1), a panel of 102 DNA samples obtained from lymphoblastoid cell lines from 4 ethnically diverse control groups, Caucasian (CA, n = 31), African American (AA, n = 24), Pacific Rim/Asian (PR, n = 24), and Hispanic (HI, n = 23). In all, the re-sequencing project covered 58,251 base pairs of genomic sequence, for a total of >5.9 million sequenced base pairs. The mean number of base pairs sequenced per gene was 9709 (range, 7007 to 13,880). On average we sequenced 3320 bases 5' of the ATG and 3282 bases 3' of the stop codon. In each case the re-sequencing spanned all exonic regions and the 3' UTR SECIS region. The re-sequencing of the *SEPP1 *locus was extended to include the exons and 5' region of an antisense transcript that overlaps the 3' UTR of the *SEPP1 *locus. Of the 235 segregating sites, the number of SNPs with a rare allele frequency ≥0.05 or ≥0.1 were 103 and 92, respectively. In this regard, we observed a small number of rare variants (Additional Files 1 to 6).

**Table 1:** Details of Sequence Analysis of 6 Selenoprotein Genes in a 102 Person Multi-ethnic Population Performed to Identify Single Nucleotide Polymorphisms

**Gene**	**Chromosome**	**Exons**	**Gene region**	**5'** **3'** **Intron**	**Total base pairs (bp) sequenced**	**Total SNPs** **NSYN** **SYN** **SECIS Region**	**SNPs/kbp**	**NSYN/kbp** **SYN/kbp**	**SNPs/kbp Gene coding region**
*GPX1*	3p21.31	2	609	3359 3431 -	7399	38 3 1 0	5.136	4.926 1.642	6.568
*GPX2*	14q24.1	2	570	3001 2896 2666	9133	47 0 0 0	5.146	- -	-
*GPX3*	5q33.1	5	678	2930 2030 2853	8491	52 0 1 0	6.124	- 1.475	1.475
*GPX4*	19p13.3	7	594	2453 1783 2177	7007	31 0 1 2	4.424	- 1.684	1.684
*TXNRD1*	12q23.3	13	1497	5341 3320 3722	13880	43 0 3 0	3.098	- 2.004	2.004
*SEPP1*	5p13.1	4	1143	2833 6231 2134	12341	24 2 0 0	1.945	1.750 -	1.750
*Total*		33	5091	53160	58251	235 5 6 0	4.034	0.982 1.179	2.161

The analysis of the possible sites of heterozygosity in the coding regions revealed several interesting observations. Of the 235 SNPs determined across the 6 genes, our analysis identified 5 non-synonymous variants, 6 synonymous variants and 224 non-coding SNPs. The coding region SNPs identified were located in the *GPX1 *(P75R, L91L, A192T, and P198L), *GPX3 *(L13L), *GPX4 *(L193L), *TXNRD1 *(L55L, L80L, and C383C), and *SEPP1 *(K19E, A234T) loci. Since sequence variation at the RNA level could in theory influence translation read through efficiency at the UGA selenocysteine codon, synonymous variants might be of particular functional relevance in selenoproteins; however, none of the identified synonymous substitutions were in the immediate vicinity of a selenocysteine codon. No putative coding region SNPs were identified in the antisense transcript that overlaps the 3' UTR of *SEPP1*. Identified non-coding SNPs included two SECIS region SNPs, both located within the *GPX4 *locus. One of these is a previously reported high frequency SNP, of possible functional significance, located 44 bp from the stop codon and just before the SECIS stem loop (stop +35 to +128) [33]. The other is a rare variant, identified in a single individual of African American/African heritage; this SNP (stop +103) is located in the vicinity of the highly conserved SECIS core. SNP density varied from 1.945 SNPs/kbp of genomic sequence in *SEPP1 *to 6.124 SNPs/kbp at the *GPX3 *locus. The mean number of SNPs/kbp for all 6 gene loci was 4.034. Perhaps reflecting greater functional constraint, the mean number of SNPs/kbp was lower in coding regions at 2.161. Within the coding region, *GPX1 *had the most SNPs/kbp (6.568) while *GPX2 *had no SNPs. Additional variation is present at the *GPX1 *and *SEPP1 *loci in the form of a variable number alanine repeat polymorphism within the first exon of *GPX1 *and a complex variable repeat polymorphism in the promoter of *SEPP1*, neither of which could be accurately resolved from our sequence tracings [34,35].

### Evolutionary analysis

We determined two measures of sequence diversity at the 6 selenoprotein loci (Table 2), the population mutation parameter (Θ) and nucleotide diversity (π). Nucleotide diversity and the population mutation parameter differ in that Θ is a measure of the number of variant sites and π is a measure of the observed heterozygosity per base pair. More specifically, nucleotide diversity is a parameter used to measure the degree of polymorphism within a population; it is defined as the average number of nucleotide differences per site between and two DNA sequences chosen randomly from the sample population. The population mutation parameter differs in that it is a measure of the observed number of variant sites, normalized to the number of chromosomes studied and the total sequence length, which corrects for sample size [36]. For the 6 genes the mean value for nucleotide diversity was 7.2 × 10^-4^. The greatest amount of nucleotide diversity (11.0 × 10^-4^) was observed at the *GPX3 *locus, while the least amount of nucleotide diversity was observed at the *TXNRD1 *locus (3.7 × 10^-4^). In general, the value for sequence diversity as measured by nucleotide diversity was similar to that measured by the population mutation parameter. For the 6 genes, the mean value for the population mutation parameter was 7.3 × 10^-4^. Under the infinite-sites model of DNA sequence evolution, if the nucleotide sequence variation among haplotypes at a locus is neutral and the sample population is in equilibrium with respect to drift and mutation, then the degree of polymorphism estimated by calculating the nucleotide diversity and the population mutation parameter should be equal. This is measured statistically using the Tajima's (D_T_) statistic [37]. A strongly negative Tajima's D test is suggestive of positive selection. In the Asian population at the *GPX1 *locus there was a strongly negative D_T _value (-1.760), however this test did not achieve statistical significance (P > 0.05, P < 0.10). Using an alternative neutrality test, the D_F _and F statistics of Fu and Li [38], however, we do detect possible evidence of selection at the *GPX1 *locus. Although non-significant for the various subpopulations, for the combined populations the values for D_F _(-2.495) and F (-2.319) are significant at the P < 0.05 level. We also observed significantly positive (P < 0.05) Tajima's D tests at the *GPX4 *(2.249) and the *SEPP1 *(2.056) loci, in the Hispanic and Caucasian populations, respectively. Although a positive D tests might be indicative of balancing selection (positive heterozygote advantage), a very plausible explanation for the positive tests in this case is the presence of a significant degree of genetic admixture within one or both of the control populations [39].

**Table 2:** Sequence Diversity and Evolutionary Analysis of 6 Selenoprotein Loci Stratified by Estimated Population

**Gene**	**Ethnic Group**	**SNPs**	**Singletons**	**SYN**	**NSYN**	**π × 10-4**	**Θ × 10-4**	**D**_**T **_**Tajima's**	**D**_**F**_	**F**
*GPX1*										
*202*	Total	38	14	1	3	5.2	8.7	-1.16256	-2.49532*	-2.31912*
*48*	AA	23	6	1	3	6.5	7.0	-0.25380	-0.23160	-0.28398
*62*	CA	15	3	0	2	5.3	4.3	0.69670	0.119874	0.38011
*46*	HI	20	10	0	2	5.1	6.2	-0.55642	-1.92897**	-1.72989
*46*	PR	15	6	0	2	2.0	4.7	-1.75961**	-1.12837	-1.58364

*GPX2*										
*204*	Total	47	11	0	0	8.2	8.7	-0.18534	-0.85302	-0.67535
*48*	AA	27	7			8.1	6.7	0.69919	-0.22648	0.11635
*62*	CA	33	7			6.7	7.7	-0.43521	0.03790	-0.16317
*46*	HI	32	8			8.7	8.0	0.31148	-0.13732	0.02421
*48*	PR	30	8			6.6	7.4	-0.36659	-0.28952	-0.37648

*GPX3*										
*204*	Total	52	6	1	0	11.0	10.4	0.18669	0.76044	0.60998
*48*	AA	42	4	1		13.9	11.1	0.84504	1.12623	1.22113
*62*	CA	30	2	0		8.6	7.5	0.47394	1.29418	1.18465
*46*	HI	38	8	1		10.1	10.2	-0.03486	0.17869	0.12254
*48*	PR	37	10	1		9.5	9.8	-0.11667	-0.32911	-0.30155

*GPX4*										
*204*	Total	31	7	1	0	10.4	7.7	0.99362	-0.65746	0.03750
*48*	AA	24	6	0		9.9	7.9	0.85392	-0.15103	0.23582
*62*	CA	21	3	0		10.2	6.5	1.77252**	0.57981	1.19930
*46*	HI	18	3	0		10.2	6.0	2.24912*	0.46442	1.26672
*48*	PR	19	3	1		10.0	6.3	1.88377**	0.53274	1.17584

*TXNRD1*										
*204*	Total	43	10	3	0	3.7	5.2	-0.84233	-0.81317	-0.99372
*48*	AA	28	5	2		4.4	4.5	-0.09852	0.40341	0.26989
*62*	CA	22	3	1		3.8	3.4	0.44241	0.64623	0.68209
*46*	HI	24	7	1		3.9	3.9	-0.02622	-0.44254	-0.35427
*48*	PR	20	7	2		2.3	3.2	-0.96694	-0.87634	-1.07578

*SEPP1*										
*204*	Total	24	5	0	2	4.8	3.3	1.25024	-0.41307	0.31238
*48*	AA	17	3		1	3.8	3.1	0.73630	0.37764	0.59147
*62*	CA	18	2		1	5.2	3.1	2.05607*	0.80818	1.48364**
*46*	HI	17	2		2	5.0	3.1	1.83966**	0.79897	1.36158
*48*	PR	16	0		1	4.5	2.9	1.66728	1.59572*	1.91681*

Single nucleotide polymorphisms (SNPs) were identified by re-sequencing 6 selenoprotein gene regions in a 102 member control population. Nucleotide diversity (π) and the population mutation parameter (Θ) were calculated using the most probable PHASED haplotypes. To determine whether variation was consistent with the expectations of the neutral equilibrium model, neutrality was tested using Tajima's (D_T_) and Fu and Li's (D_F _and F) statistics. D_T_, D_F _and F were computed using S, the number of segregating sites, in DNASP.

*Statistical significance: P < 0.05

**Statistical significance: P < 0.10, P > 0.05

### Confirmation of recent positive selection using data from the HapMap Project

**Figure 1:**
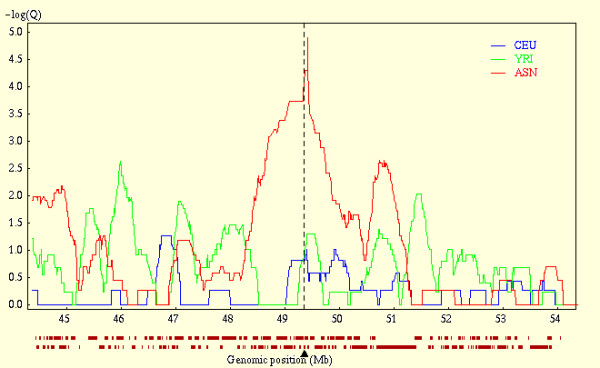
**Confirmation of recent positive selection at the *GPX1 *locus (3p21)**. To confirm recent positive selection at the *GPX1 *locus, we used Haplotter to query the results of a scan for positive selection in the human genome developed using SNP data from the International HapMap project [41]. The vertical line indicates the location of the *GPX1 *locus. The strong iHS (integrated haplotype score) signal in the Asian (ASN) population at this locus is highly suggestive for recent positive selection. Data is based on the analysis of unrelated individuals from 3 populations: ASN (Han Chinese and Japanese, n = 89), CEU (Northern and Western European, n = 60), and YRI (Sub-Saharan Africans from the Yoruban population, n = 60).

To confirm recent positive selection at the *GPX1 *locus, we used the web application Haplotter, developed in the Pritchard laboratory, to query a map of recent positive selection in the human genome. The input SNP data for this map are derived from the Phase 1 International HapMap Project [40]. Strong evidence for recent positive selection, as evidenced by a strong iHS (integrated haplotype score) signal, supports the hypothesis that the *GPX1 *locus has undergone a recent selective sweep in the Asian Population (Figure 1) [41]. Strong signatures of positive selection were not observed at the *GPX2*, *GPX3*, *GPX4*, *TXNRD1 *or *SEPP1 *loci in any of the subpopulations.

### Genetic difference between sample groups

The proportion of the total genetic variance (*Fst*) contained in a subpopulation relative to the total genetic variance was calculated (Table 3). The data from the re-sequencing of the SNP500Cancer population suggest that there is some evidence for specific differences in genotype distribution between different ethnic groups, especially at the *GPX1 *locus. At the *GPX1 *locus the estimation of population subdivision between the Pacific Rim/Asian and the African American/African populations was 0.2418, and between the Pacific Rim/Asian and the Caucasian populations it was 0.2682. Altogether, these data suggest that there is evidence for specific differences in genotype distribution between the different ethnic groups, especially at the *GPX1 *locus.

**Table 3:** Estimation of Population Subdivision (*Fst*) at 6 Selenoprotein Loci

**GPX1**	**HI**	**AA**	**PR**
**AA**	0.0718		
**PR**	0.0882	0.2418	
**CA**	0.0441	0.0631	0.2682
**GPX2**	**HI**	**AA**	**PR**
**AA**	0.0825		
**PR**	0.0739	0.1347	
**CA**	0.0178	0.2105	0.1127
**GPX3**	**HI**	**AA**	**PR**
**AA**	0.0667		
**PR**	0.0388	0.1553	
**CA**	0.0048	0.1273	0.0318
**GPX4**	**HI**	**AA**	**PR**
**AA**	0.0761		
**PR**	-0.0100	0.0535	
**CA**	-0.0175	0.0915	-0.0038
**SEPP1**	**HI**	**AA**	**PR**
**AA**	0.0589		
**PR**	-0.0110	0.1023	
**CA**	-0.0104	0.0649	0.0096
**TXNRD1**	**HI**	**AA**	**PR**
**AA**	-0.0078		
**PR**	0.0801	0.1088	
**CA**	-0.0137	0.0066	0.0836
**All 6 Genes**	**HI**	**AA**	**PR**
**AA**	0.0610		
**PR**	0.0381	0.1290	
**CA**	0.0022	0.1057	0.0684

*Fst *was calculated by pairwise comparison of all subpopulations. A high *Fst* implies a considerable degree of differentiation among populations. The negative *Fst* values noted in some cells should be interpreted that there is no genetic differentiation between the two population and likely reflects the imprecision of the algorithm used by the software to estimate this value.

### Haplotype structure

**Figure 2:**
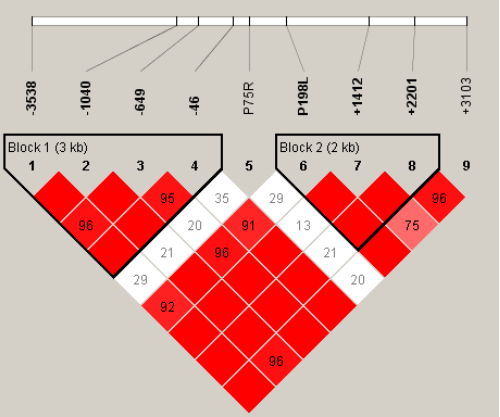
**Estimates for linkage disequilibrium (LD) and location of major haplotype blocks across 6 selenoprotein loci**. Pair wise plots (D') across 6 selenoprotein loci based on genotype data obtained from re-sequencing the 102 person multiethnic SNP500 DNA population, which is comprised of individuals of AA, CA, HI and PR heritage. LD plots for the various ethnic subpopulations are available as supplementary data. Re-sequenced genes include *GPX1 *(**Figure 2**), *GPX2 *(**Figure 3**), *GPX3 *(**Figure 4**), *GPX4 *(**Figure 5**), *SEPP1 *(**Figure 6**), and *TXNRD1 *(**Figure 7**). SNP identifiers are indicated on the abscissas. Numbers within cells correspond to LD values (D'). The LD color scheme is stratified according to the logarithm of the odds (LOD) score and D': LOD <2 (white for D'<1 and blue for D' = 1) or LOD >2 (shades of pink/red for D'<1 and bright red for D' = 1). Haplotype blocks were created using the algorithm of Gabriel et al, Science 2002 [76]. 95% confidence bounds on D' were generated and each comparison was called "strong LD", "inconclusive" or "strong recombination". A block was created if 95% of informative comparisons were "strong LD".

**Figure 3:**
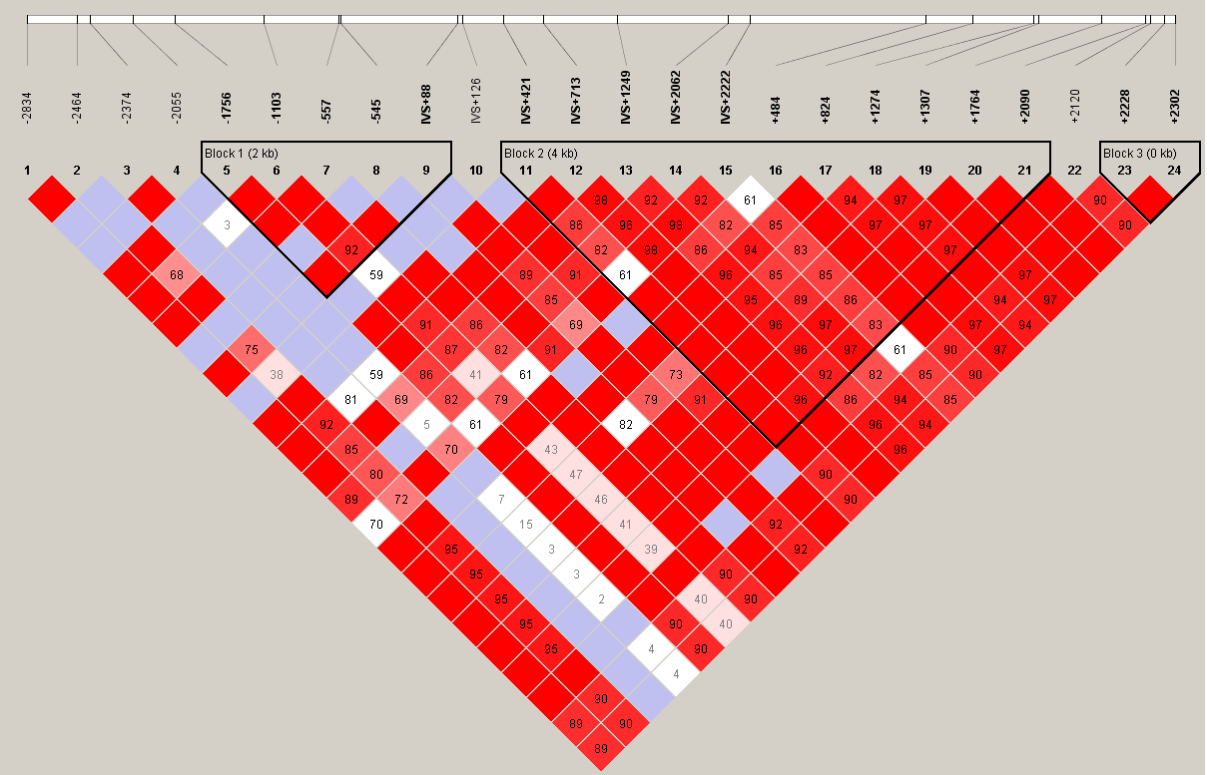
**Estimates for linkage disequilibrium (LD) and location of major haplotype blocks across 6 selenoprotein loci**. Pair wise plots (D') across 6 selenoprotein loci based on genotype data obtained from re-sequencing the 102 person multiethnic SNP500 DNA population, which is comprised of individuals of AA, CA, HI and PR heritage. LD plots for the various ethnic subpopulations are available as supplementary data. Re-sequenced genes include *GPX1 *(**Figure 2**), *GPX2 *(**Figure 3**), *GPX3 *(**Figure 4**), *GPX4 *(**Figure 5**), *SEPP1 *(**Figure 6**), and *TXNRD1 *(**Figure 7**). SNP identifiers are indicated on the abscissas. Numbers within cells correspond to LD values (D'). The LD color scheme is stratified according to the logarithm of the odds (LOD) score and D': LOD <2 (white for D'<1 and blue for D' = 1) or LOD >2 (shades of pink/red for D'<1 and bright red for D' = 1). Haplotype blocks were created using the algorithm of Gabriel et al, Science 2002 [76]. 95% confidence bounds on D' were generated and each comparison was called "strong LD", "inconclusive" or "strong recombination". A block was created if 95% of informative comparisons were "strong LD".

**Figure 4:**
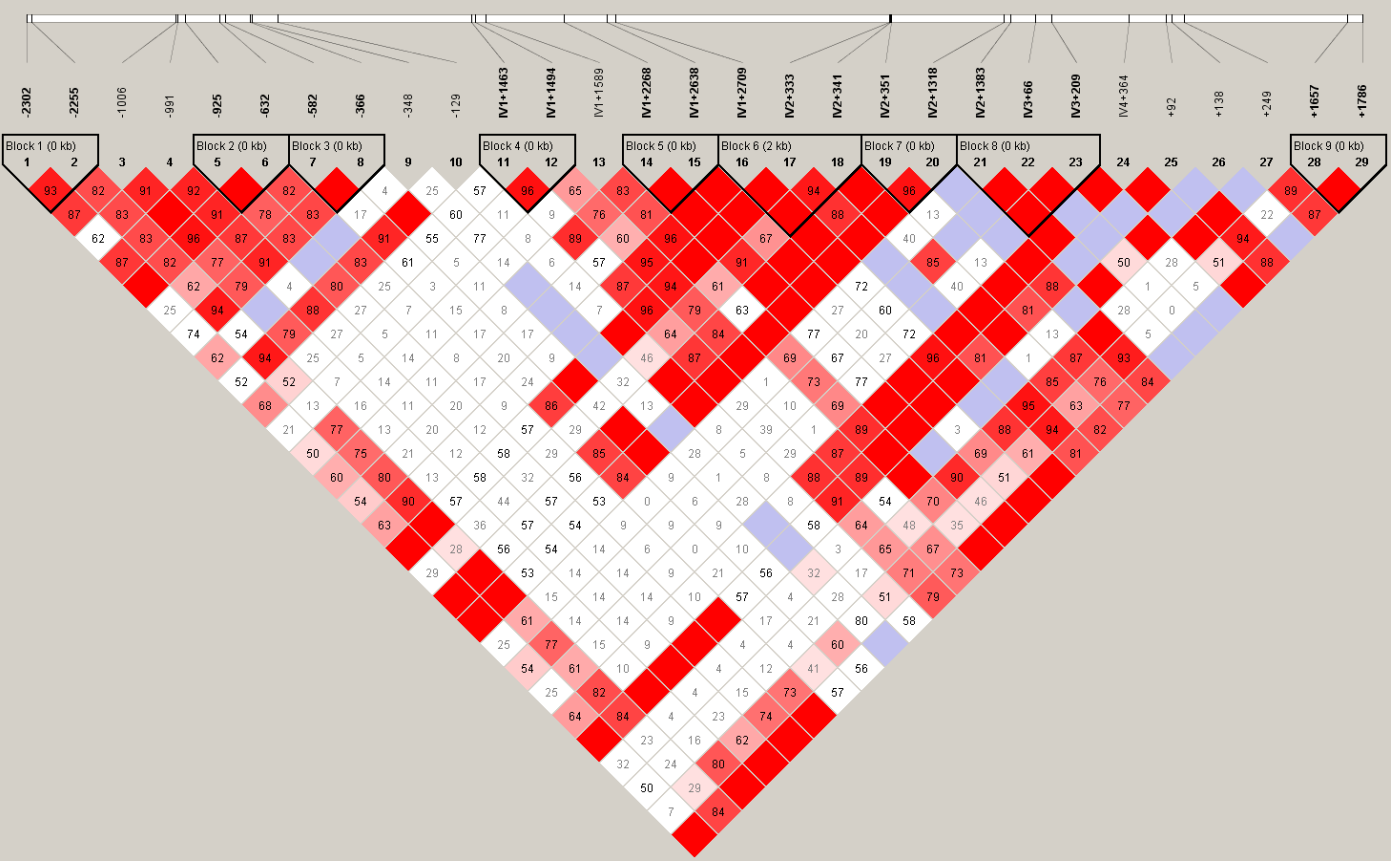
**Estimates for linkage disequilibrium (LD) and location of major haplotype blocks across 6 selenoprotein loci**. Pair wise plots (D') across 6 selenoprotein loci based on genotype data obtained from re-sequencing the 102 person multiethnic SNP500 DNA population, which is comprised of individuals of AA, CA, HI and PR heritage. LD plots for the various ethnic subpopulations are available as supplementary data. Re-sequenced genes include *GPX1 *(**Figure 2**), *GPX2 *(**Figure 3**), *GPX3 *(**Figure 4**), *GPX4 *(**Figure 5**), *SEPP1 *(**Figure 6**), and *TXNRD1 *(**Figure 7**). SNP identifiers are indicated on the abscissas. Numbers within cells correspond to LD values (D'). The LD color scheme is stratified according to the logarithm of the odds (LOD) score and D': LOD <2 (white for D'<1 and blue for D' = 1) or LOD >2 (shades of pink/red for D'<1 and bright red for D' = 1). Haplotype blocks were created using the algorithm of Gabriel et al, Science 2002 [76]. 95% confidence bounds on D' were generated and each comparison was called "strong LD", "inconclusive" or "strong recombination". A block was created if 95% of informative comparisons were "strong LD".

**Figure 5:**
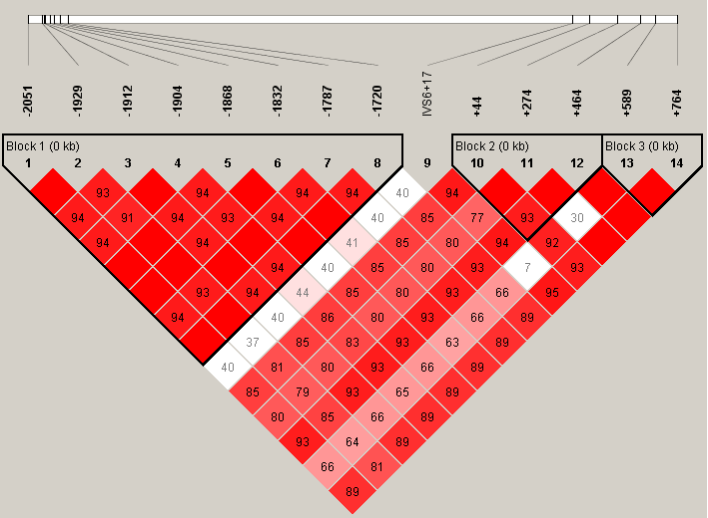
**Estimates for linkage disequilibrium (LD) and location of major haplotype blocks across 6 selenoprotein loci**. Pair wise plots (D') across 6 selenoprotein loci based on genotype data obtained from re-sequencing the 102 person multiethnic SNP500 DNA population, which is comprised of individuals of AA, CA, HI and PR heritage. LD plots for the various ethnic subpopulations are available as supplementary data. Re-sequenced genes include *GPX1 *(**Figure 2**), *GPX2 *(**Figure 3**), *GPX3 *(**Figure 4**), *GPX4 *(**Figure 5**), *SEPP1 *(**Figure 6**), and *TXNRD1 *(**Figure 7**). SNP identifiers are indicated on the abscissas. Numbers within cells correspond to LD values (D'). The LD color scheme is stratified according to the logarithm of the odds (LOD) score and D': LOD <2 (white for D'<1 and blue for D' = 1) or LOD >2 (shades of pink/red for D'<1 and bright red for D' = 1). Haplotype blocks were created using the algorithm of Gabriel et al, Science 2002 [76]. 95% confidence bounds on D' were generated and each comparison was called "strong LD", "inconclusive" or "strong recombination". A block was created if 95% of informative comparisons were "strong LD".

**Figure 6:**
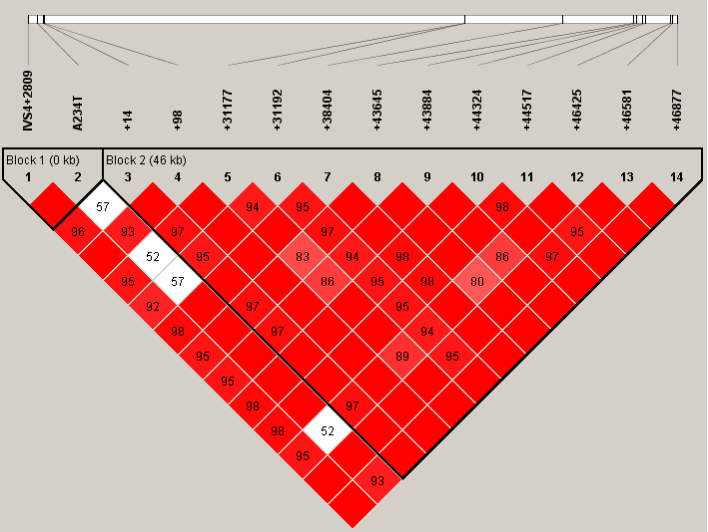
**Estimates for linkage disequilibrium (LD) and location of major haplotype blocks across 6 selenoprotein loci**. Pair wise plots (D') across 6 selenoprotein loci based on genotype data obtained from re-sequencing the 102 person multiethnic SNP500 DNA population, which is comprised of individuals of AA, CA, HI and PR heritage. LD plots for the various ethnic subpopulations are available as supplementary data. Re-sequenced genes include *GPX1 *(**Figure 2**), *GPX2 *(**Figure 3**), *GPX3 *(**Figure 4**), *GPX4 *(**Figure 5**), *SEPP1 *(**Figure 6**), and *TXNRD1 *(**Figure 7**). SNP identifiers are indicated on the abscissas. Numbers within cells correspond to LD values (D'). The LD color scheme is stratified according to the logarithm of the odds (LOD) score and D': LOD <2 (white for D'<1 and blue for D' = 1) or LOD >2 (shades of pink/red for D'<1 and bright red for D' = 1). Haplotype blocks were created using the algorithm of Gabriel et al, Science 2002 [76]. 95% confidence bounds on D' were generated and each comparison was called "strong LD", "inconclusive" or "strong recombination". A block was created if 95% of informative comparisons were "strong LD".

**Figure 7:**
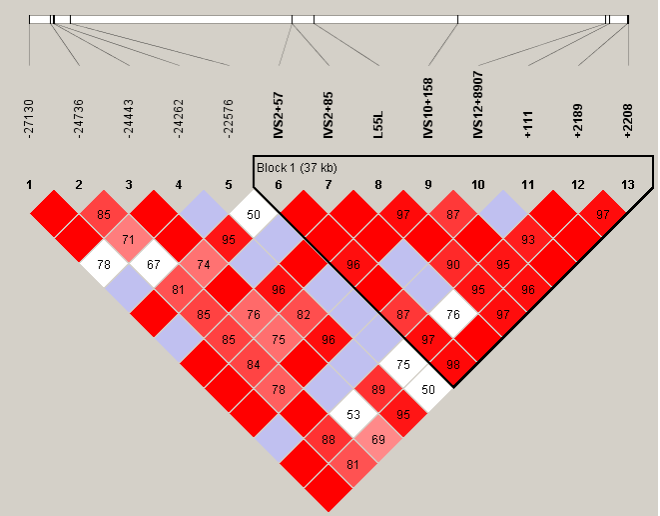
**Estimates for linkage disequilibrium (LD) and location of major haplotype blocks across 6 selenoprotein loci**. Pair wise plots (D') across 6 selenoprotein loci based on genotype data obtained from re-sequencing the 102 person multiethnic SNP500 DNA population, which is comprised of individuals of AA, CA, HI and PR heritage. LD plots for the various ethnic subpopulations are available as supplementary data. Re-sequenced genes include *GPX1 *(**Figure 2**), *GPX2 *(**Figure 3**), *GPX3 *(**Figure 4**), *GPX4 *(**Figure 5**), *SEPP1 *(**Figure 6**), and *TXNRD1 *(**Figure 7**). SNP identifiers are indicated on the abscissas. Numbers within cells correspond to LD values (D'). The LD color scheme is stratified according to the logarithm of the odds (LOD) score and D': LOD <2 (white for D'<1 and blue for D' = 1) or LOD >2 (shades of pink/red for D'<1 and bright red for D' = 1). Haplotype blocks were created using the algorithm of Gabriel et al, Science 2002 [76]. 95% confidence bounds on D' were generated and each comparison was called "strong LD", "inconclusive" or "strong recombination". A block was created if 95% of informative comparisons were "strong LD".

The most probable PHASED haplotypes derived using SNPs with minimum rare allele frequencies of ≥5% are presented as supplementary data (Additional Files 7 to 12). Estimates for linkage disequilibrium (LD) and location of major haplotype blocks across each of the 6 selenoprotein loci are provided for the total population in Figures 2 to 7 and for each of the individual ethnic groups in the supplementary data files (Additional File 13). For the data set, the haplotype diversity is restricted and the number of unique haplotypes varied by gene locus from 16 (*GPX1*) to 51 (*GPX3*), with a mean of 28.2. In most cases, the African American population had the greatest number of unique haplotypes (mean 14.8), whereas the Pacific Rim/Asian population had the fewest (mean 9.2). The number of common haplotypes with a frequency of ≥0.05 ranged from 3 (*GPX3*) to 5 (*GPX1*, *SEPP1 *and *TXNRD1*). Examined in a population specific manner, we also noted variation in the frequency of these major haplotypes. At the *GPX1 *locus, for example, haplotype number 1 was observed in 63% of individuals of Pacific Rim/Asian heritage, whereas the frequency of this major haplotype was much lower in the other populations (AA 0.17, CA 0.13 and HI 0.30). Similarly, at the *TXNRD1 *locus haplotype 1 had a frequency of 65% in the Pacific Rim/Asian population, but was observed less often in the other populations (AA 0.29, CA 0.19, and HI 0.28). Although the functional significance of the various imputed haplotypes remains to be determined, it is of interest to note that key SNPs of possible functional consequences segregate with particular haplotypes. For example, the T variant of a common *GPX4 *SECIS region SNP (Stop +44) is found in haplotype 1 but not in any of the 8 next most common *GPX4 *haplotypes. Similarly, for the *GPX1 *P198L variant, the proline variant (C) resides on the 4 most common *GPX1 *haplotypes whereas the lucine variant (T) is only observed on the backbone of several rarer haplotypes (5, 6, 9, 10, 13, and 14); these rarer haplotypes are relatively uncommon among individuals of Pacific Rim/Asian heritage. In addition, there is a common non-synonymous (A234T) variant in *SEPP1*, located between 2 histidine rich regions. This variant is a major distinguishing feature between the most common *SEPP1 *haplotype (0.36) and the next most common haplotype (0.18). Again it is notable that the T234 encoding haplotypes (2, 6 and 10) are rare in the Asian/Pacific Rim Populations, with respective frequencies of only 0.04, 0.02 and 0.

## Discussion

Selenium deficiency impairs the production of selenium containing proteins and may be a risk factor for cancer, infectious disease severity and enhanced susceptibility to oxidant stressors. Recently, selenium has emerged as one of the most promising cancer chemoprevention agents and is the focus of a large clinical trial (SELECT) that has enrolled 35,000 men to determine if selenium supplementation prevents prostate cancer[3,4,42]. It is possible that the anticancer properties of selenium are mediated through selenoproteins, many of which have antioxidant properties. An alternative hypothesis, however, suggests that the anticancer property of selenium compounds occurs at doses beyond those that are required to ensure maximal selenoprotein production [43,44]. If selenoproteins play a direct role in cancer chemoprevention, then it is possible that genetic variation in selenoprotein activity or expression might also modify susceptibility to genome damaging environmental exposures such as cigarette smoke or dietary carcinogens. Similarly, it is also possible that inter-individual variation in selenoprotein expression could modify disease outcomes by influencing major antioxidant pathways, such as the glutathione cycle or thioredoxin metabolism. Pathways relevant not only to cancer susceptibility, but also to chemotherapy induced toxicities [45], and infectious disease severity (i.e., viral myocarditis, malaria, and septic shock syndrome) [46-48]. We therefore explored the genetic variation in 6 selenoprotein genes in order to provide the foundation for the comprehensive analysis of selenoprotein genetic variation in candidate gene association studies. In this regard we have re-sequenced 6 of the 25 known human selenoprotein genes to identify common SNPs and haplotypes and to explore the selective processes acting on these loci. The genes selected for re-sequencing and evolutionary analysis are among the best-studied selenoproteins and all have important antioxidant properties; they include 4 glutathione peroxidases (*GPX1-4*), *SEPP1 *and *TXNRD1 *[6-8].

In total, we sequenced approximately 5.9 million base pairs of DNA from 102 individuals, representative of 4 ethnic populations common within the United States, Caucasian (CA, n = 31), African American (AA, n = 24), Pacific Rim/Asian (PR, n = 24), and Hispanic (HI, n = 23). We identified 235 SNPs, of which 103 had a rare allele frequency of greater than 0.05. For the 6 selenoprotein genes the mean value for nucleotide diversity was 7.2 × 10^-4^, which is similar to the value of 6.7 × 10^-4 ^obtained by the Environmental Genome Project which recently re-sequenced 213 genes in 90 individuals[49]. Particularly interesting SNPs (with minimum rare allele frequency ≥0.05), of potential functional importance, include the *GPX1 *P75R and P198L variants, a high frequency *GPX4 *SECIS region SNP, and an A234T non-synonymous variant in *SEPP1*. The *GPX1 *P198L and *GPX4 *SECIS SNPs have both been previously described [16,33]. In addition, we identified a rare *GPX4 *SECIS SNP adjacent to the SECIS core. SECIS SNPs are of particular interest, as this RNA stem loop structure is required for the translational incorporation of the amino acid selenocysteine. In the absence of a functional SECIS, translation will terminate prematurely at the UGA-selenocysteine codon. At this point, the functional significance of the identified SNPs and haplotypes remains largely uncharacterized. Although there is data suggesting that each GPX1-L198 allele decreases red cell glutathione peroxidase activity by about 5%, attempts to correlate enzyme activities with specific genotypes have provided inconsistent results, perhaps reflective of the observation that selenium status may influence selenoprotein expression or enzymatic activity[16,33,50]. Moreover, it is possible that haplotype analysis may provide a better means for correlating enzymatic activity or serum selenium levels, especially if this is done in individuals maintained on a diet containing optimal supplemental selenium.

Overall the pattern of the observed genetic variation was consistent with the expectations of the neutral equilibrium model of evolution for 5 genes, but at the *GPX1 *locus we found evidence for selection. At the *GPX1 *locus, the D_F _and F statistics of Fu and Li were strongly negative. The presence of a significantly negative D value indicates the presence of an excess of rare alleles inconsistent with neutral processes in a stable population, but consistent with either a demographic or selective processes [38]. The fact that a similar phenomenon is not observed at the other loci, suggests that the phenomenon is not simply the result of a demographic process such as a recent population expansion. Additional support for selection at the *GPX1 *locus is provided by the negative value for the Tajima's test (-1.760) in the Pacific Rim/Asian population, which just missed achieving statistical significance (P > 0.05, P < 0.10). Of further interest, we also found evidence for differences in genotype distribution between different ethnic groups, especially at the *GPX1 *locus. The relatively high *Fst *values of 0.2418 (Pacific Rim/Asian vs. African American/African) and of 0.2682 (Pacific Rim/Asian vs. Caucasian) suggest that there is substantial genetic differentiation between these populations. Inspection of the major *GPX1 *haplotypes in the Pacific Rim/Asian population reveals that the P198 containing haplotypes predominate and that the L198 variant is rarely observed. Moreover, this is consistent with reports that the L198 variant was not observed among individuals of Chinese heritage [51].

Whether the relative absence of L198 haplotypes within the Asian population is the result of a recent selective sweep, perhaps in response to an environmental or infectious exposure, cannot be determined from our data set. However, strong confirmation for a recent selective sweep involving chromosome region 3p21, which includes the *GPX1 *locus, is provided by analysis of SNP data from the International HapMap project [40,41]. The strong iHS signal observed in the Asian population at this locus is one of the highest observed on Chromosome 3 and is highly suggestive for recent positive selection (Figure 1) [41]. A selective sweep at the *GPX1 *locus may explain an earlier observation that there is significantly less variation in red cell glutathione peroxidase activity among individuals of Asian heritage compared to what is observed in Occidental Populations [52]. Understanding whether functional variants of *GPX1*, or other genes at the 3p21 locus, confer protection or susceptibility in disease populations may provide insight into the selective pressures responsible for this recent selective sweep.

The genomic locations of several selenoprotein genes are of particular interest. For example, there is strong LD between the *GPX1 *P198L variant and variants in the nearby gene *RHOA*. Since *RHOA *belongs to the ras oncogene family and studies both *in vitro *and *in vivo *suggest that its overexpression may lead to cancer [53,54], it is possible that observed associations between the L198 variant and an increased risk of cancer may in fact be due to LD between this variant and as yet unidentified variants within *RHOA *or another nearby gene [51]. Similarly, it is of great interest that the 3p21 genomic region also includes the gene for α-dystroglycan (*DAG1*), which encodes for a peripheral membrane protein used as a cellular receptor for arenaviruses, the causative agents of fatal hemorrhagic fevers, and also as the Schwann cell receptor for *M. leprae *[55,56]. Likewise, it is also worth noting that *SEPP1 *is located at chromosome position 5p13.1, close to chromosomal regions that contain the growth hormone receptor and alpha-methylacyl-CoAracemase, genes of potential relevance to cancer susceptibility [57,58]. We also note here the presence of an antisense transcript that overlaps the 3' UTR of *SEPP1*. Since some antisense transcripts post-transcriptionally regulate the expression of the overlapping transcript, we extended our resequencing at the *SEPP1 *locus to include the antisense transcript. Future studies utilizing these data will be able to explore if this antisense transcript plays a role in the regulation of *SEPP1*.

## Conclusion

Genetic variation across selenoprotein genes could be of great interest to not only association testing strategies but also to strategies to investigate the pattern of molecular evolution in a group of genes with a distinctive feature, the incorporation of the amino acid selenocysteine. The 6 genes re-sequenced in this project include some of the best characterized selenoproteins, most of which have important antioxidant properties. It is likely that additional selenoproteins also play a role in pathways relevant to cancer and disease susceptibility, such as endoplasmic reticulum stress response and inflammation [59,60]. The potential importance of selenoproteins in a wide array of human diseases including cancer, heart disease, aging and infections coupled with the promise of selenium as a chemoprevention agent warrants further investigation of the role of these and other selenoproteins in human disease. We believe that the study of selenoproteins provides a unique model system for exploring the complex interaction between genes and environmental exposures. The fine haplotype maps described in this report will be useful for exploring associations between selenoprotein variants and diseases, studying selenoprotein loss of heterozygosity in tumor samples, or for correlating selenoprotein genotypes with serum selenium levels or selenoenzyme activity in patients enrolled on clinical trials using selenium as a chemoprevention agent[61].

## Methods

### Population

The control population used for re-sequencing is the SNP500Cancer DNA panel, which represents a subset of the available DNA Polymorphism Discovery Resource [62]. The SNP500Cancer set consists of DNAs from 102 lymphoblastoid cell lines from 4 ethnically diverse groups, 31 Caucasian-Americans (CA), 24 African/African-Americans (AA), 24 Pacific Rim/Asian-Americans (PR), and 23 Hispanic-Americans (HI). The use of these publicly available panels, which are anonymized except for information about ethnic group and gender, for re-sequencing was deemed exempt from Institutional Review Board (IRB) approval by the Johns Hopkins University IRB. Genotype data and validated assays for genotyping select haplotype tagged SNPs identified through this re-sequencing project and for additional unrelated loci are publicly available as part of the cancer genome anatomy project at the SNP500 website [63].

### PCR primers and sequencing

For each selenoprotein gene the full coding sequence and approximately 3000 bases of the 5' promoter and 3' UTR were re-sequenced. Overlapping PCR products of approximately 500 bases were designed using Primer 3 (Additional File 14)[64]. Each forward primer was tagged with a universal M13 forward sequence (5'-TGTAAAACGACGGCCAGT-3') and each reverse primer was tagged with a universal M13 reverse sequence (5'-CAGGAAACAGCTATGACC). The reliability of the sequencing data was ensured by sequencing in both directions, and in the case of most high frequency SNPs results were further confirmed by an independent genotype methods performed through the SNP500 genotyping project [63]. Primers were designed to include all exons, intron/exon borders, the 5' UTR and the 3' UTR, including SECIS elements. For some small regions, we were unable to obtain good quality sequence, despite multiple attempts at primer redesign and optimization. PCR and DNA sequencing reactions were amplified on MJ Research Tetrad thermalcyclers. Big Dye Terminator chemistry sequencing reactions were run in either 96 well or 384 well format on ABI 3700 capillary automatic sequencers. Forward and reverse sequence tracing were aligned in Sequencher 4.2 (Gene Codes, Ann Arbor, MI) and SNPs were determined by visual inspection. SNP data was placed in "prettybase" format and summary statistics and Hardy-Weinberg Equilibrium calculations were performed using software available through the Innate Immunity PGA [65]. Prettybase files, the reference sequences used to assign prettybase SNP locations, and gaps in sequence coverage are available as supplementary data for each gene (Additional Files 15, 16, 17, 18, 19, 20, 21).

### Mapping DATA

For the purpose of mapping SNPs and primer locations we used the May 2004 assembly of the human genome (Build 43, NCBI). Genomic sequences between the most 5' forward PCR primer and the most 3' reverse PCR primer were obtained using the UCSC In-Silico PCR program [66]. The location of each SNP was mapped onto the gene structure relative to the following Entrez RefSeq curated mRNA sequences (Additional Files 22, 23, 24, 25, 26, 27): *GPX1 *(NM_000581), *GPX2 *(NM_002083), *GPX3 *(NM_002084), *GPX4 *(NM_002085), *TXNRD1 *(NM_003330), and *SEPP1 *(NM_005410). SNPs 5' of the ATG are represented as a negative number relative to the first base of the start codon; SNP 3' of the stop are represented as a positive number relative to the last base of the stop codon; SNPs within an intron are represented as intron number plus the number of bases from the first base of the start of the intron; SNPs within an exon are represented as either synonymous (SYN) or non-synonymous (NSYN) and the amino acid position is provided. In the case of *GPX1*, the resequencing in the 5' direction extended into the coding region of a neighboring gene, ras homolog gene family member A (*RHOA*). Of note, there is an uncharacterized phylogenetically conserved transcript (BC039102) overlapping the 3' end of *SEPP1 *in an antisense orientation. Resequencing at the *SEPP1 *locus was expanded to include putative exons and the promoter region corresponding to this antisense transcript because of the possibility that overlapping transcripts might post-transcriptionally regulate each other's expression [67,68]. *TXNRD1 *exhibits alternative splicing at the 5' end. Our re-sequencing corresponded to the exons of *TXNRD1 *transcript variant 1 (NM_003330) and also included the published promoter region, which is conserved between the mouse and human[69].

### Evolutionary analysis

To compare the sequence diversity between genes, the heterozygosity per nucleotide site was estimated by calculating nucleotide diversity (π) and the population mutation parameter (Θ) [70]. To determine whether the observed variation was consistent with the expectations of the neutral equilibrium model of evolution, neutrality was tested using Tajima's (D_T_) and Fu and Li's (D_F _and F) statistics [37,38]. The most probable imputed PHASED haplotypes were used as input sequence for evolutionary analyses in the software program DNASP [71]. PHASED haplotypes were estimated using the Bayesian statistical method in PHASE2.0 run either locally or off the Innate Immunity web site [65,72]. PHASE output was transformed into the proper DNASP input format using the perl script phasetodnasp-v2.1.pl written and kindly provided by Eduardo Tarazona Santos (Section of Genomic Variation, Pediatric Oncology Branch, NCI, NIH, Bethesda, MD). Genomic regions for which sequence data was not available were excluded from various population genetic analyses (Additional File 21). Evidence for specific differences in genotype distribution between the various ethnic groups was explored by calculating the allele identity F-statistic (*FST*) for all population pairs using GENEPOP on the Web, developed from the Genepop DOS versions 3.3/3.4[73]. *FST *is the proportion of the total genetic variance contained in a subpopulation (s) relative to the total genetic variance (t). Values can range from 0 to 1. High *FST *implies a considerable degree of differentiation among populations. GENEPOP is a population genetics software package originally designed by Michel Raymond and Francois Rousset, at the Laboratiore de Genetique et Environment, Montpellier, France. Transformation of data from prettybase format to GENEPOP format was facilitated by using the perl script report_prettybase.pl written by Fares Z. Najar (revised by James D. White) at the Advanced Center for Genome Technology, University of Oklahoma. To confirm recent positive selection at the *GPX1 *locus, we used Haplotter to query the results of a scan for positive selection in the human genome developed using SNP data from the International HapMap project [40,41,74]. The iHS is a new test for detecting recent positive selection developed by the Pritchard laboratory and based on the extended haplotype homozygosity (EHH) statistic proposed by Sabeti et al [75].

### Haplotype structure and patterns of Linkage Disequilibrium (LD)

For each gene the most probable PHASED haplotype was determined, as described above, using only those SNPs that had a minimum rare allele frequency of ≥0.05. Using the Chimp BLAT Search at the UCSC Genome Bioinformatics Site, we aligned the human locus of interest and the corresponding locus from the chimp genome (Nov. 2003 assembly) to infer a chimp haplotype [66]. LD (D') between pairs of variants (minimum rare allele frequency of ≥0.05) was computed using the software program Haploview 3.2, using the most probable PHASED haplotypes as the input sequence. Using Haploview, haplotype blocks were created using the algorithm of Gabriel et al, Science 2002 [76]. 95% confidence bounds on D' were generated and each comparison was called "strong LD", "inconclusive" or "strong recombination". A block was created if 95% of informative comparisons were "strong LD". To identify a set of htSNPs for each gene, we used the Haploview's tagger feature with the following default settings: pairwise tagging only, r^2 ^threshold 0.8 and LOD threshold for multi-marker tests 3.0. Of note, htSNPs are selected on a block-by-block basis; therefore, the end set of htSNPs is not necessarily the most parsimonious one for the entire data set, but is more likely to capture variation in a new, larger data set that was not observed in the initial data set. Non-synonymous SNPs and SECIS region SNPs with a minimum rare allele frequency of ≥0.05 were force included as tagged SNPs.

## Authors' contributions

CBF conceived the idea for the study and is the principal investigator on an NIH K22 research award that provided partial funds for the re-sequencing. He oversaw and contributed to all aspects of the re-sequencing project, helped organize genotype data, performed all genetic analyses and authored the manuscript. KA contributed to all technical aspects of the project, performed most of the PCR and DNA sequencing and organized the genotype database. SJC helped develop the SNP500Cancer control population and database, provided input into study design and contributed to the revision of the manuscript. He is the director of the NIH core genotyping facility where sequencing reactions were run. HFM performed PCR amplification and re-sequencing of the *SEPP1 *locus and participated in the revision of the manuscript. UP is the principal investigator for an NIH intramural research award that provided funding for sequencing reagents. She contributed to the conception of the project, study design and the revision of the manuscript. All authors read and approved the final manuscript.

## Supplementary Material

Additional File 1 **Genotype Frequencies and Hardy-Weinberg Equilibrium (HWE) Calculations for Single Nucleotide Polymorphisms (SNPs) at the GPX1 Locus.** Genotype frequencies and HWE calculations are provided for each of the 4 ethnic subpopulations, AA (n = 24), CA (n = 31), HI (n = 23), and PR (n = 24; n = 23 for GPX1). RS# refers to the SNPs reference cluster ID, a unique SNP ID assigned by dbSNP[77]. Genotype data for identified SNPs have been made available through the SNP500 Cancer database. Where RS# are not yet assigned, the SNP500 Cancer ID# has been provided [63]. Location refers to SNP position relative to the ATG, Stop codon, or Intron/Exon position mapped to the provided genomic reference sequences. Similarly, the Prettybase ID# provides the location of each nucleotide variant/SNP, but refers to the nucleotide sequence position relative to the start of the genomic reference sequence. GPX1 Genotype Frequencies. Genotype frequencies, RS#, SNP location and Hardy-Weinberg equilibrium data is provided for all GPX1 SNPs in this file. Click here for file

Additional File 2 **Genotype Frequencies and Hardy-Weinberg Equilibrium (HWE) Calculations for Single Nucleotide Polymorphisms (SNPs) at the GPX2 Locus.** Genotype frequencies and HWE calculations are provided for each of the 4 ethnic subpopulations, AA (n = 24), CA (n = 31), HI (n = 23), and PR (n = 24; n = 23 for GPX1). RS# refers to the SNPs reference cluster ID, a unique SNP ID assigned by dbSNP[77]. Genotype data for identified SNPs have been made available through the SNP500 Cancer database. Where RS# are not yet assigned, the SNP500 Cancer ID# has been provided [63]. Location refers to SNP position relative to the ATG, Stop codon, or Intron/Exon position mapped to the provided genomic reference sequences. Similarly, the Prettybase ID# provides the location of each nucleotide variant/SNP, but refers to the nucleotide sequence position relative to the start of the genomic reference sequence. GPX2 Genotype Frequencies. Genotype frequencies, RS#, SNP location and Hardy-Weinberg equilibrium data is provided for all GPX2 SNPs in this file. Click here for file

Additional File 3 **Genotype Frequencies and Hardy-Weinberg Equilibrium (HWE) Calculations for Single Nucleotide Polymorphisms (SNPs) at the GPX3 Locus.** Genotype frequencies and HWE calculations are provided for each of the 4 ethnic subpopulations, AA (n = 24), CA (n = 31), HI (n = 23), and PR (n = 24; n = 23 for GPX1). RS# refers to the SNPs reference cluster ID, a unique SNP ID assigned by dbSNP[77]. Genotype data for identified SNPs have been made available through the SNP500 Cancer database. Where RS# are not yet assigned, the SNP500 Cancer ID# has been provided [63]. Location refers to SNP position relative to the ATG, Stop codon, or Intron/Exon position mapped to the provided genomic reference sequences. Similarly, the Prettybase ID# provides the location of each nucleotide variant/SNP, but refers to the nucleotide sequence position relative to the start of the genomic reference sequence. GPX3 Genotype Frequencies. Genotype frequencies, RS#, SNP location and Hardy-Weinberg equilibrium data is provided for all GPX3 SNPs in this file. Click here for file

Additional File 4 **Genotype Frequencies and Hardy-Weinberg Equilibrium (HWE) Calculations for Single Nucleotide Polymorphisms (SNPs) at the GPX4 Locus.** Genotype frequencies and HWE calculations are provided for each of the 4 ethnic subpopulations, AA (n = 24), CA (n = 31), HI (n = 23), and PR (n = 24; n = 23 for GPX1). RS# refers to the SNPs reference cluster ID, a unique SNP ID assigned by dbSNP[77]. Genotype data for identified SNPs have been made available through the SNP500 Cancer database. Where RS# are not yet assigned, the SNP500 Cancer ID# has been provided [63]. Location refers to SNP position relative to the ATG, Stop codon, or Intron/Exon position mapped to the provided genomic reference sequences. Similarly, the Prettybase ID# provides the location of each nucleotide variant/SNP, but refers to the nucleotide sequence position relative to the start of the genomic reference sequence. GPX4 Genotype Frequencies. Genotype frequencies, RS#, SNP location and Hardy-Weinberg equilibrium data is provided for all GPX4 SNPs in this file. Click here for file

Additional File 5 **Genotype Frequencies and Hardy-Weinberg Equilibrium (HWE) Calculations for Single Nucleotide Polymorphisms (SNPs) at the SEPP1 Locus.** Genotype frequencies and HWE calculations are provided for each of the 4 ethnic subpopulations, AA (n = 24), CA (n = 31), HI (n = 23), and PR (n = 24; n = 23 for GPX1). RS# refers to the SNPs reference cluster ID, a unique SNP ID assigned by dbSNP[77]. Genotype data for identified SNPs have been made available through the SNP500 Cancer database. Where RS# are not yet assigned, the SNP500 Cancer ID# has been provided [63]. Location refers to SNP position relative to the ATG, Stop codon, or Intron/Exon position mapped to the provided genomic reference sequences. Similarly, the Prettybase ID# provides the location of each nucleotide variant/SNP, but refers to the nucleotide sequence position relative to the start of the genomic reference sequence. SEPP1 Genotype Frequencies. Genotype frequencies, RS#, SNP location and Hardy-Weinberg equilibrium data is provided for all SEPP1 SNPs in this file. Click here for file

Additional File 6 **Genotype Frequencies and Hardy-Weinberg Equilibrium (HWE) Calculations for Single Nucleotide Polymorphisms (SNPs) at the TXNRD1 Locus.** Genotype frequencies and HWE calculations are provided for each of the 4 ethnic subpopulations, AA (n = 24), CA (n = 31), HI (n = 23), and PR (n = 24; n = 23 for GPX1). RS# refers to the SNPs reference cluster ID, a unique SNP ID assigned by dbSNP[77]. Genotype data for identified SNPs have been made available through the SNP500 Cancer database. Where RS# are not yet assigned, the SNP500 Cancer ID# has been provided [63]. Location refers to SNP position relative to the ATG, Stop codon, or Intron/Exon position mapped to the provided genomic reference sequences. Similarly, the Prettybase ID# provides the location of each nucleotide variant/SNP, but refers to the nucleotide sequence position relative to the start of the genomic reference sequence. TXNRD1 Genotype Frequencies. Genotype frequencies, RS#, SNP location and Hardy-Weinberg equilibrium data is provided for all TXNRD1 SNPs in this file. Click here for file

Additional File 7 **Most Probable PHASED Haplotypes at the GPX1 Locus Determined Using Only Those SNPs With a Minimum Rare Allele Frequency of ≥ 0.05.** Haplotype frequencies are provided for the combined SNP500 DNA population (n = 102), and for each of the 4 ethnic subpopulations, AA (n = 24), CA (n = 31), HI (n = 23), and PR (n = 24; n = 23 for GPX1). RS# refers to the SNPs reference cluster ID, a unique SNP ID assigned by dbSNP[77]. Location refers to SNP position relative to the ATG, Stop codon, or Intron/Exon position mapped to the provided genomic reference sequences. Similarly, the Prettybase ID# provides the location of each nucleotide variant/SNP, but refers to the nucleotide sequence position relative to the start of the genomic reference sequence. For convenience, we have identified a possible subset of SNPs for each gene that are most likely to capture the full variation at the locus in a new, larger data set. These so called haplotype tagged SNPs (htSNPs) are indicated by the word Yes. For reference purposes, an imputed Chimp haplotype was determined by aligning the human locus of interest to the Chimp genome using the Chimp BLAT Search program at the UCSC Genome Bioinformatics Site. GPX1 Haplotype Frequencies. The software program PHASE was used to define haplotypes for the GPX1 locus. Haplotype frequencies for each ethnic population, SNP locations, RS#, and htSNP data are provided. Click here for file

Additional File 8 **Most Probable PHASED Haplotypes at the GPX2 Locus Determined Using Only Those SNPs With a Minimum Rare Allele Frequency of ≥ 0.05.** Haplotype frequencies are provided for the combined SNP500 DNA population (n = 102), and for each of the 4 ethnic subpopulations, AA (n = 24), CA (n = 31), HI (n = 23), and PR (n = 24; n = 23 for GPX1). RS# refers to the SNPs reference cluster ID, a unique SNP ID assigned by dbSNP[77]. Location refers to SNP position relative to the ATG, Stop codon, or Intron/Exon position mapped to the provided genomic reference sequences. Similarly, the Prettybase ID# provides the location of each nucleotide variant/SNP, but refers to the nucleotide sequence position relative to the start of the genomic reference sequence. For convenience, we have identified a possible subset of SNPs for each gene that are most likely to capture the full variation at the locus in a new, larger data set. These so called haplotype tagged SNPs (htSNPs) are indicated by the word Yes. For reference purposes, an imputed Chimp haplotype was determined by aligning the human locus of interest to the Chimp genome using the Chimp BLAT Search program at the UCSC Genome Bioinformatics Site. GPX2 Haplotype Frequencies. The software program PHASE was used to define haplotypes for the GPX2 locus. Haplotype frequencies for each ethnic population, SNP locations, RS#, and htSNP data are provided. Click here for file

Additional File 9 **Most Probable PHASED Haplotypes at the GPX3 Locus Determined Using Only Those SNPs With a Minimum Rare Allele Frequency of ≥ 0.05.** Haplotype frequencies are provided for the combined SNP500 DNA population (n = 102), and for each of the 4 ethnic subpopulations, AA (n = 24), CA (n = 31), HI (n = 23), and PR (n = 24; n = 23 for GPX1). RS# refers to the SNPs reference cluster ID, a unique SNP ID assigned by dbSNP[77]. Location refers to SNP position relative to the ATG, Stop codon, or Intron/Exon position mapped to the provided genomic reference sequences. Similarly, the Prettybase ID# provides the location of each nucleotide variant/SNP, but refers to the nucleotide sequence position relative to the start of the genomic reference sequence. For convenience, we have identified a possible subset of SNPs for each gene that are most likely to capture the full variation at the locus in a new, larger data set. These so called haplotype tagged SNPs (htSNPs) are indicated by the word Yes. For reference purposes, an imputed Chimp haplotype was determined by aligning the human locus of interest to the Chimp genome using the Chimp BLAT Search program at the UCSC Genome Bioinformatics Site. GPX3 Haplotype Frequencies. The software program PHASE was used to define haplotypes for the GPX3 locus. Haplotype frequencies for each ethnic population, SNP locations, RS#, and htSNP data are provided. Click here for file

Additional File 10 **Most Probable PHASED Haplotypes at the GPX4 Locus Determined Using Only Those SNPs With a Minimum Rare Allele Frequency of ≥ 0.05.** Haplotype frequencies are provided for the combined SNP500 DNA population (n = 102), and for each of the 4 ethnic subpopulations, AA (n = 24), CA (n = 31), HI (n = 23), and PR (n = 24; n = 23 for GPX1). RS# refers to the SNPs reference cluster ID, a unique SNP ID assigned by dbSNP[77]. Location refers to SNP position relative to the ATG, Stop codon, or Intron/Exon position mapped to the provided genomic reference sequences. Similarly, the Prettybase ID# provides the location of each nucleotide variant/SNP, but refers to the nucleotide sequence position relative to the start of the genomic reference sequence. For convenience, we have identified a possible subset of SNPs for each gene that are most likely to capture the full variation at the locus in a new, larger data set. These so called haplotype tagged SNPs (htSNPs) are indicated by the word Yes. For reference purposes, an imputed Chimp haplotype was determined by aligning the human locus of interest to the Chimp genome using the Chimp BLAT Search program at the UCSC Genome Bioinformatics Site. GPX4 Haplotype Frequencies. The software program PHASE was used to define haplotypes for the GPX4 locus. Haplotype frequencies for each ethnic population, SNP locations, RS#, and htSNP data are provided. Click here for file

Additional File 11 **Most Probable PHASED Haplotypes at the SEPP1 Locus Determined Using Only Those SNPs With a Minimum Rare Allele Frequency of ≥ 0.05.** Haplotype frequencies are provided for the combined SNP500 DNA population (n = 102), and for each of the 4 ethnic subpopulations, AA (n = 24), CA (n = 31), HI (n = 23), and PR (n = 24; n = 23 for GPX1). RS# refers to the SNPs reference cluster ID, a unique SNP ID assigned by dbSNP[77]. Location refers to SNP position relative to the ATG, Stop codon, or Intron/Exon position mapped to the provided genomic reference sequences. Similarly, the Prettybase ID# provides the location of each nucleotide variant/SNP, but refers to the nucleotide sequence position relative to the start of the genomic reference sequence. For convenience, we have identified a possible subset of SNPs for each gene that are most likely to capture the full variation at the locus in a new, larger data set. These so called haplotype tagged SNPs (htSNPs) are indicated by the word Yes. For reference purposes, an imputed Chimp haplotype was determined by aligning the human locus of interest to the Chimp genome using the Chimp BLAT Search program at the UCSC Genome Bioinformatics Site. SEPP1 Haplotype Frequencies. The software program PHASE was used to define haplotypes for the SEPP1 locus. Haplotype frequencies for each ethnic population, SNP locations, RS#, and htSNP data are provided. Click here for file

Additional File 12 **Most Probable PHASED Haplotypes at the TXNRD1 Locus Determined Using Only Those SNPs With a Minimum Rare Allele Frequency of ≥ 0.05.** Haplotype frequencies are provided for the combined SNP500 DNA population (n = 102), and for each of the 4 ethnic subpopulations, AA (n = 24), CA (n = 31), HI (n = 23), and PR (n = 24; n = 23 for GPX1). RS# refers to the SNPs reference cluster ID, a unique SNP ID assigned by dbSNP[77]. Location refers to SNP position relative to the ATG, Stop codon, or Intron/Exon position mapped to the provided genomic reference sequences. Similarly, the Prettybase ID# provides the location of each nucleotide variant/SNP, but refers to the nucleotide sequence position relative to the start of the genomic reference sequence. For convenience, we have identified a possible subset of SNPs for each gene that are most likely to capture the full variation at the locus in a new, larger data set. These so called haplotype tagged SNPs (htSNPs) are indicated by the word Yes. For reference purposes, an imputed Chimp haplotype was determined by aligning the human locus of interest to the Chimp genome using the Chimp BLAT Search program at the UCSC Genome Bioinformatics Site. TXNRD1 Haplotype Frequencies. The software program PHASE was used to define haplotypes for the TXNRD1 locus. Haplotype frequencies for each ethnic population, SNP locations, RS#, and htSNP data are provided. Click here for file

Additional File 13 **Estimates for linkage disequilibrium (LD) and location of major haplotype blocks across 6 selenoprotein loci, stratified by ethnic subpopulation**. Pair wise plots (D') across 6 selenoprotein loci based on genotype data obtained from re-sequencing DNA samples from individuals of AA (n = 24), CA (n = 31), HI (n = 23) and PR (n = 24; n = 23 for GPX1)heritage from the SNP500 DNA population. Re-sequenced genes include a) *GPX1*, b) *GPX2*, c) *GPX3*, d) *GPX4*, e) *SEPP1*, and f) *TXNRD1*. SNP identifiers are indicated on the abscissas. Numbers within cells correspond to LD values (D'). The LD color scheme is stratified according to the logarithm of the odds (LOD) score and D': LOD <2 (white for D'<1 and blue for D' = 1) or LOD >2 (shades of pink/red for D'<1 and bright red for D' = 1). Haplotype blocks were created using the algorithm of Gabriel et al, Science 2002 [76]. 95% confidence bounds on D' were generated and each comparison was called "strong LD", "inconclusive" or "strong recombination". A block was created if 95% of informative comparisons were "strong LD". LD Plots For Ethnic Subpopulations. Estimation of linkage disequilibrium (D') and the location of major haplotype blocks across each of the six selenoprotein loci is provided; the data in this file is stratified by ethnic subpopulation. Click here for file

Additional File 14 PCR Primer Pairs. This file provides the name, location and sequence for PCR primers used in the resequencing project. Click here for file

Additional File 15 GPX1 Prettybase File. This file is in prettybase format and provides SNP location in the reference sequence, SNP500 sample ID number, and genotype calls for each identified SNP at the GPX1 locus. Click here for file

Additional File 16 GPX2 Prettybase File. This file is in prettybase format and provides SNP location in the reference sequence, SNP500 sample ID number, and genotype calls for each identified SNP at the GPX2 locus. Click here for file

Additional File 17 GPX3 Prettybase File. This file is in prettybase format and provides SNP location in the reference sequence, SNP500 sample ID number, and genotype calls for each identified SNP at the GPX3 locus. Click here for file

Additional File 18 GPX4 Prettybase File. This file is in prettybase format and provides SNP location in the reference sequence, SNP500 sample ID number, and genotype calls for each identified SNP at the GPX4 locus. Click here for file

Additional File 19 SEPP1 Prettybase File. This file is in prettybase format and provides SNP location in the reference sequence, SNP500 sample ID number, and genotype calls for each identified SNP at the SEPP1 locus. Click here for file

Additional File 20 TXNRD1 Prettybase File. This file is in prettybase format and provides SNP location in the reference sequence, SNP500 sample ID number, and genotype calls for each identified SNP at the TXNRD1 locus. Click here for file

Additional File 21 Regions Covered in Resequencing. This file provides the information on the regions of the reference sequence for which we were able to get good quality sequence data, allowing identification of gaps in sequence coverage or regions that could not be resequenced. Click here for file

Additional File 22 GPX1 Genomic Sequence. This file provides the chromosomal location and genomic DNA sequence for GPX1 and is the reference used map the location of SNPs in the prettybase file and PCR primer pair file. Click here for file

Additional File 23 GPX2 Genomic Sequence. This file provides the chromosomal location and genomic DNA sequence for GPX2 and is the reference used map the location of SNPs in the prettybase file and PCR primer pair file. Click here for file

Additional File 24 GPX3 Genomic Sequence. This file provides the chromosomal location and genomic DNA sequence for GPX3 and is the reference used map the location of SNPs in the prettybase file and PCR primer pair file. Click here for file

Additional File 25 GPX4 Genomic Sequence. This file provides the chromosomal location and genomic DNA sequence for GPX4 and is the reference used map the location of SNPs in the prettybase file and PCR primer pair file. Click here for file

Additional File 26 SEPP1 Genomic Sequence. This file provides the chromosomal location and genomic DNA sequence for SEPP1 and is the reference used map the location of SNPs in the prettybase file and PCR primer pair file. Click here for file

Additional File 27 TXNRD1 Genomic Sequence. This file provides the chromosomal location and genomic DNA sequence for TXNRD1 and is the reference used map the location of SNPs in the prettybase file and PCR primer pair file. Click here for file
