# Supplementary material for: Polymorphism analysis of six selenoprotein genes: support for a selective sweep at the glutathione peroxidase 1 locus (3p21) in Asian populations
Source: BMC Genet. 2006 Dec 11;7:56. doi: 10.1186/1471-2156-7-56 (PMC1769511; doi:10.1186/1471-2156-7-56)
Supplement: Additional File 1 — Genotype Frequencies and Hardy-Weinberg Equilibrium (HWE) Calculations for Single Nucleotide Polymorphisms (SNPs) at the GPX1 Locus. Genotype frequencies and HWE calculations are provided for each of the 4 ethnic subpopulations, AA (n = 24), CA (n = 31), HI (n = 23), and PR (n = 24; n = 23 for GPX1). RS# refers to the SNPs reference cluster ID, a unique SNP ID assigned by dbSNP[77]. Genotype data for identified SNPs have been made available through the SNP500 Cancer database. Where RS# are not yet assigned, the SNP500 Cancer ID# has been provided [63]. Location refers to SNP position relative to the ATG, Stop codon, or Intron/Exon position mapped to the provided genomic reference sequences. Similarly, the Prettybase ID# provides the location of each nucleotide variant/SNP, but refers to the nucleotide sequence position relative to the start of the genomic reference sequence. GPX1 Genotype Frequencies. Genotype frequencies, RS#, SNP location and Hardy-Weinberg equilibrium data is provided for all GPX1 SNPs in this file. [file 1471-2156-7-56-S1.pdf]

Genotype Frequency For Glutathione Peroxidase 1 (GPX1) SNPs

**RS# or SNP500Cancer ID#**	**Location**	**Prettybase ID#**	**Frequencies**						**HWE P Values**				
			Variant	AA	CA	HI	PR	Avg	AA	CA	H	PR	Avg
rs1987628	-3538	85	C:C	0.542	0.7	0.739	0.913	0.72	0.289	1	1	1	0.686
			C:T	0.458	0.267	0.261	0.087	0.27					
			T:T	0	0.033	0	0	0.01					
	-3106	527	A:A	0	0	0	0	0	1	1	1	1	1
			A:G	0.087	0	0	0	0.02					
			G:G	0.913	1	1	1	0.98					
	-3013	620	C:C	0.958	1	1	1	0.99	1	1	1	1	1
			C:G	0.042	0	0	0	0.01					
			G:G	0	0	0	0	0					
	-2753	880	C:C	1	0.966	0.957	1	0.98	1	1	1	1	1
			C:T	0	0.034	0.043	0	0.02					
			T:T	0	0	0	0	0					
	-2737	896	C:C	1	1	0.957	1	0.99	1	1	1	1	1
			C:G	0	0	0.043	0	0.01					
			G:G	0	0	0	0	0					
	-2249	1384	C:C	0.958	1	1	1	0.99	1	1	1	1	1
			C:T	0.042	0	0	0	0.01					
			T:T	0	0	0	0	0					
	-1704	1929	C:C	0.958	1	1	1	0.99	1	1	1	1	1
			C:T	0.042	0	0	0	0.01					
			T:T	0	0	0	0	0					
rs8179164	-1573	2060	A:A	1	0.935	1	1	0.98	1	1	1	1	1
			A:T	0	0.065	0	0	0.02					
			T:T	0	0	0	0	0					
	-1203	2430	A:A	0	0	0	0	0	1	1	1	1	1
			A:G	0	0	0.043	0	0.01					
			G:G	1	1	0.957	1	0.99					
rs8179165	-1133	2500	C:C	0	0	0	0	0	1	1	1	1	1
			C:T	0.042	0	0	0	0.01					
			T:T	0.958	1	1	1	0.99					
rs3448	-1040	2593	A:A	0.083	0.226	0.087	0	0.109	1	1	0.61	1	0.223
			A:G	0.458	0.484	0.348	0.087	0.356					
			G:G	0.458	0.29	0.565	0.913	0.535					
GPX1-12	-929	2704	A:A	0.875	1	1	1	0.97	1	1	1	1	1
			A:G	0.125	0	0	0	0.03					
			G:G	0	0	0	0	0					
	-728	2905	C:C	1	1	0.957	1	0.99	1	1	1	1	1
			C:T	0	0	0.043	0	0.01					
			T:T	0	0	0	0	0					
rs3811699	-649	2984	A:A	0.583	0.677	0.739	0.913	0.723	1	0.29	0.41	1	0.264
			A:G	0.375	0.258	0.217	0.087	0.238					
			G:G	0.042	0.065	0.043	0	0.04					
rs8179167	-105	3528	C:C	0.917	1	1	1	0.978	1	1	1	1	1
			C:G	0.083	0	0	0	0.022					
			G:G	0	0	0	0	0					
rs1800668	-46	3587	C:C	0.583	0.789	0.789	0.957	0.776	0.539	0.26	1	1	1
			C:T	0.417	0.158	0.211	0.043	0.212					
			T:T	0	0.053	0	0	0.012					
GPX1-08	P75R	3862	C:C	0.625	0.129	0.391	0.696	0.436	0.113	0.22	0.01	0	0
			C:G	0.25	0.323	0.217	0.087	0.228					
			G:G	0.125	0.548	0.391	0.217	0.337					
	L91L	4188	C:C	0.958	1	1	1	0.99	1	1	1	1	1
			C:T	0.042	0	0	0	0.01					
			T:T	0	0	0	0	0					
rs6446261	A192T	4491	A:A	0	0	0	0	0	1	1	1	1	1
			A:G	0.083	0	0	0	0.02					
			G:G	0.917	1	1	1	0.98					
rs1050450	P198L	4510	C:C	0.542	0.71	0.739	0.913	0.723	1	1	0.41	1	0.698
			C:T	0.417	0.258	0.217	0.087	0.248					
			T:T	0.042	0.032	0.043	0	0.03					
SECIS Region		4563-4657											
	Stop +227	4750	C:C	0	0	0	0	0	1	1	1	1	1
			C:T	0	0	0.045	0	0.01					
			T:T	1	1	0.955	1	0.99					

	Stop +425	4948	C:C	1	1	0.957	1	0.99	1	1	1	1	1
			C:T	0	0	0.043	0	0.01					
			T:T	0	0	0	0	0					
	Stop +564	5087	C:C	0	0	0	0	0	1	1	1	1	1
			C:T	0.083	0	0	0	0.02					
			T:T	0.917	1	1	1	0.98					
	Stop +1148	5671	C:C	1	1	1	0.957	0.99	1	1	1	1	1
			C:T	0	0	0	0.043	0.01					
			T:T	0	0	0	0	0					
	Stop +1300	5823	A:A	0	0	0	0	0	1	1	1	1	1
			A:G	0	0	0	0.043	0.01					
			G:G	1	1	1	0.957	0.99					
	Stop +1366	5889	A:A	0	0	0	0	0	1	1	1	1	1
			A:G	0.125	0	0	0	0.03					
			G:G	0.875	1	1	1	0.97					
rs6790105	Stop +1412	5935	A:A	0.25	0.29	0.565	0.913	0.485	0.403	1	0.3	1	0.257
			A:G	0.625	0.484	0.304	0.087	0.386					
			G:G	0.125	0.226	0.13	0	0.129					
	Stop +1425	5948	C:C	0.958	1	1	0.913	0.97	1	1	1	1	1
			C:T	0.042	0	0	0.087	0.03					
			T:T	0	0	0	0	0					
rs8179172	Stop +1554	6077	A:A	0	0	0	0	0	1	1	1	1	1
			A:T	0.25	0	0.043	0	0.069					
			T:T	0.75	1	0.957	1	0.931					
	Stop +1761	6284	C:C	1	1	0.957	1	0.99	1	1	1	1	1
			C:T	0	0	0.043	0	0.01					
			T:T	0	0	0	0	0					
	Stop +1798	6321	C:C	0.917	1	1	1	0.98	1	1	1	1	1
			C:T	0.083	0	0	0	0.02					
			T:T	0	0	0	0	0					
	Stop +2002	6525	A:A	0	0	0	0	0	1	1	1	1	1
			A:G	0	0.032	0.043	0.043	0.03					
			G:G	1	0.968	0.957	0.957	0.97					
	Stop +2157	6680	C:C	0	0	0.043	0	0.01	1	1	0.07	1	0.049
			C:G	0	0.032	0.043	0.043	0.03					
			G:G	1	0.968	0.913	0.957	0.96					
rs11720657	Stop +2201	6724	C:C	0.458	0.29	0.565	0.913	0.535	1	1	0.61	1	0.223
			C:T	0.458	0.484	0.348	0.087	0.356					
			T:T	0.083	0.226	0.087	0	0.109					
	Stop +2322	6845	C:C	1	1	1	0.957	0.99	1	1	1	1	1
			C:T	0	0	0	0.043	0.01					
			T:T	0	0	0	0	0					
GPX1-19	Stop +2617	7140	C:C	0	0.032	0	0	0.01	1	0.1	1	1	0.049
			C:G	0	0.065	0.043	0	0.03					
			G:G	1	0.903	0.957	1	0.96					
	Stop +3103	7626	C:C	0.75	0.452	0.1	0	0.311	1	0.7	0.65	1	0.021
			C:T	0.25	0.419	0.55	0.261	0.378					
			T:T	0	0.129	0.35	0.739	0.311					
	Stop +3107	7630	C:C	1	0.935	1	1	0.978	1	1	1	1	1
			C:T	0	0.065	0	0	0.022					
			T:T	0	0	0	0	0					
