# Supplementary material for: Polymorphism analysis of six selenoprotein genes: support for a selective sweep at the glutathione peroxidase 1 locus (3p21) in Asian populations
Source: BMC Genet. 2006 Dec 11;7:56. doi: 10.1186/1471-2156-7-56 (PMC1769511; doi:10.1186/1471-2156-7-56)
Supplement: Additional File 2 — Genotype Frequencies and Hardy-Weinberg Equilibrium (HWE) Calculations for Single Nucleotide Polymorphisms (SNPs) at the GPX2 Locus. Genotype frequencies and HWE calculations are provided for each of the 4 ethnic subpopulations, AA (n = 24), CA (n = 31), HI (n = 23), and PR (n = 24; n = 23 for GPX1). RS# refers to the SNPs reference cluster ID, a unique SNP ID assigned by dbSNP[77]. Genotype data for identified SNPs have been made available through the SNP500 Cancer database. Where RS# are not yet assigned, the SNP500 Cancer ID# has been provided [63]. Location refers to SNP position relative to the ATG, Stop codon, or Intron/Exon position mapped to the provided genomic reference sequences. Similarly, the Prettybase ID# provides the location of each nucleotide variant/SNP, but refers to the nucleotide sequence position relative to the start of the genomic reference sequence. GPX2 Genotype Frequencies. Genotype frequencies, RS#, SNP location and Hardy-Weinberg equilibrium data is provided for all GPX2 SNPs in this file. [file 1471-2156-7-56-S2.pdf]

Genotype Frequency for Glutathione Peroxidase 2 (GPX2) SNPs

RS#	Location	Prettybase ID#	Frequencies					
			Variant	AA	CA	HI	PR	Avg
	-2943	59	A:A	0	0	0	0	0
			A:G	0	0	0	0.042	0.01
			G:G	1	1	1	0.958	0.99
rs2277502	-2834	168	A:A	0	0.04	0.043	0	0.021
			A:G	0	0.12	0.087	0.125	0.083
			G:G	1	0.84	0.87	0.875	0.896
rs3742599	-2464	538	G:G	0.792	0.767	0.696	0.875	0.782
			G:T	0.167	0.2	0.174	0.125	0.168
			T:T	0.042	0.033	0.13	0	0.05
	-2374	628	C:C	0.625	1	0.913	1	0.892
			C:T	0.375	0	0.087	0	0.108
			T:T	0	0	0	0	0
	-2322	680	C:C	0	0	0	0	0
			C:T	0.042	0	0	0	0.01
			T:T	0.958	1	1	1	0.99
	-2080	922	A:A	0	0	0	0.042	0.01
			A:G	0	0	0	0.042	0.01
			G:G	1	1	1	0.917	0.98
	-2072	930	C:C	0	0	0	0	0
			C:T	0.083	0	0	0	0.02
			T:T	0.917	1	1	1	0.98
rs3825644	-2055	947	A:A	0.5	0.968	0.826	0.958	0.824
			A:C	0.458	0.032	0.13	0.042	0.157
			C:C	0.042	0	0.043	0	0.02
	-2037	965	A:A	0	0	0	0	0
			A:C	0	0	0.043	0	0.01
			C:C	1	1	0.957	1	0.99
	-1944	1058	C:C	1	0.968	1	1	0.99
			C:G	0	0.032	0	0	0.01
			G:G	0	0	0	0	0
	-1943	1059	G:G	0	0	0	0	0
			G:T	0	0.032	0	0	0.01
			T:T	1	0.968	1	1	0.99
	-1937	1065	C:C	0	0	0	0	0
			C:G	0	0	0	0.042	0.01
			G:G	1	1	1	0.958	0.99
rs17880531	-1756	1246	A:A	0	0.032	0.043	0	0.02
			A:G	0	0.161	0.13	0.125	0.108
			G:G	1	0.806	0.826	0.875	0.873
	-1692	1310	A:A	0.958	1	1	1	0.99
			A:G	0.042	0	0	0	0.01
			G:G	0	0	0	0	0
	-1624	1378	C:C	1	0.968	0.957	1	0.98
			C:G	0	0.032	0.043	0	0.02
			G:G	0	0	0	0	0
rs17880145	-1104	1898	C:C	1	0.933	1	1	0.98
			C:T	0	0.067	0	0	0.02
			T:T	0	0	0	0	0

rs2296327	-1103	1899	A:A	0.087	0.067	0.045	0.417	0.152
			A:G	0.522	0.167	0.364	0.417	0.354
			G:G	0.391	0.767	0.591	0.167	0.495
rs17102360	-876	2126	C:C	0	0	0	0	0
			C:T	0.174	0	0.136	0.042	0.081
			T:T	0.826	1	0.864	0.958	0.919
rs17880939	-557	2445	A:A	1	0.8	0.818	0.875	0.87
			A:G	0	0.167	0.136	0.125	0.11
			G:G	0	0.033	0.045	0	0.02
rs17093568	-545	2457	G:G	1	0.966	0.955	0.304	0.816
			G:T	0	0.034	0.045	0.522	0.143
			T:T	0	0	0	0.174	0.041
	-393	2609	C:C	0	0	0	0	0
			C:T	0	0.033	0	0.042	0.02
			T:T	1	0.967	1	0.958	0.98
rs1800669	IVS+18	3242	A:A	0	0	0	0	0
			A:T	0	0.097	0	0	0.029
			T:T	1	0.903	1	1	0.971
rs17882875	IVS+88	3312	G:G	1	0.778	0.783	0.833	0.847
			G:T	0	0.185	0.174	0.083	0.112
			T:T	0	0.037	0.043	0.083	0.041
rs17880758	IVS+126	3350	A:A	0.042	0	0	0	0.01
			A:G	0.25	0.074	0.217	0	0.133
			G:G	0.708	0.926	0.783	1	0.857
	IVS+298	3522	A:A	0.958	1	1	1	0.99
			A:C	0.042	0	0	0	0.01
			C:C	0	0	0	0	0
rs17880303	IVS+421	3645	C:C	0	0.037	0.043	0	0.02
			C:T	0	0.185	0.13	0.125	0.112
			T:T	1	0.778	0.826	0.875	0.867
	IVS+530	3754	C:C	0.917	1	1	1	0.98
			C:T	0.083	0	0	0	0.02
			T:T	0	0	0	0	0
	IVS+712	3936	C:C	0	0	0	0	0
			C:G	0.042	0	0	0	0.01
			G:G	0.958	1	1	1	0.99
rs17884367	IVS+713	3937	C:C	0.042	0.533	0.429	0.167	0.303
			C:T	0.333	0.4	0.333	0.375	0.364
			T:T	0.625	0.067	0.238	0.458	0.333
rs17881779	IVS+1249	4473	C:C	0.625	0.074	0.227	0.435	0.333
			C:T	0.375	0.407	0.273	0.391	0.365
			T:T	0	0.519	0.5	0.174	0.302
	IVS+1335	4559	A:A	0	0	0	0	0
			A:G	0.083	0	0.045	0	0.031
			G:G	0.917	1	0.955	1	0.969
rs4902346	IVS+2062	5286	C:C	0.273	0.032	0.182	0.083	0.131
			C:T	0.455	0.161	0.318	0.167	0.263
			T:T	0.273	0.806	0.5	0.75	0.606
rs2071566	IVS+2222	5446	A:A	0.682	0.032	0.227	0.458	0.323
			A:G	0.318	0.323	0.318	0.375	0.333
			G:G	0	0.645	0.455	0.167	0.343
SECIS Region		6441-6535						

rs17886370	Stop+443	6683	C:C	0	0	0	0	0
			C:T	0.042	0	0	0	0.01
			T:T	0.958	1	1	1	0.99
rs17884597	Stop+484	6724	A:A	0	0.033	0.045	0	0.02
			A:C	0	0.167	0.091	0.125	0.101
			C:C	1	0.8	0.864	0.875	0.879
	Stop+513	6753	A:A	0	0	0	0	0
			A:G	0	0	0.043	0	0.01
			G:G	1	1	0.957	1	0.99
rs17883891	Stop+824	7064	C:C	0.292	0.677	0.545	0.75	0.574
			C:T	0.458	0.29	0.318	0.167	0.307
			T:T	0.25	0.032	0.136	0.083	0.119
rs17880850	Stop+945	7185	A:A	0	0	0	0	0
			A:G	0	0.097	0.043	0.042	0.049
			G:G	1	0.903	0.957	0.958	0.951
rs17881498	Stop+1274	7514	C:C	0.208	0.033	0.2	0	0.103
			C:T	0.333	0.3	0.3	0.217	0.289
			T:T	0.458	0.667	0.5	0.783	0.608
rs17880380	Stop+1307	7547	C:C	0.429	0.583	0.467	0.571	0.5
			C:T	0.429	0.417	0.333	0.143	0.354
			T:T	0.143	0	0.2	0.286	0.146
rs17881134	Stop+1764	8004	G:G	0.25	0.032	0.13	0.083	0.118
			G:T	0.458	0.29	0.348	0.167	0.314
			T:T	0.292	0.677	0.522	0.75	0.569
rs17886858	Stop+2090	8330	C:C	0.25	0.032	0.174	0.083	0.127
			C:G	0.458	0.29	0.304	0.167	0.304
			G:G	0.292	0.677	0.522	0.75	0.569
rs17883358	Stop+2120	8360	C:C	1	0.806	0.826	0.875	0.873
			C:T	0	0.161	0.13	0.125	0.108
			T:T	0	0.032	0.043	0	0.02
rs17883073	Stop+2147	8387	C:C	0.958	0.871	0.957	0.958	0.931
			C:T	0.042	0.129	0.043	0.042	0.069
			T:T	0	0	0	0	0
rs10132858	Stop+2228	8468	C:C	0.25	0.032	0.13	0.083	0.118
			C:T	0.458	0.29	0.348	0.125	0.304
			T:T	0.292	0.677	0.522	0.792	0.578
rs4902345	Stop+2302	8542	A:A	0.292	0.677	0.522	0.792	0.578
			A:G	0.458	0.29	0.348	0.125	0.304
			G:G	0.25	0.032	0.13	0.083	0.118
	Stop+2681	8921	A:A	0.958	0.871	0.957	0.958	0.931
			A:T	0.042	0.129	0.043	0.042	0.069
			T:T	0	0	0	0	0

HWE P Values

AA	CA	HI	PR	Avg
1	1	1	1	1
1	0.198	0.132	1	0.037
0.298	0.414	0.035	1	0.014
0.551	1	1	1	1
1	1	1	1	1
1	1	1	0.064	0.015
1	1	1	1	1
0.636	1	0.214	1	0.236
1	1	1	1	1
1	1	1	1	1
1	1	1	1	1
1	1	1	1	1
1	0.316	0.214	1	0.083
1	1	1	1	1
1	1	1	1	1
1	1	1	1	1

0.662	0.099	1	0.667	0.066
1	1	1	1	1
1	0.325	0.224	1	0.086
1	1	1	1	0.016
1	1	1	1	1
1	1	1	1	1
1	0.358	0.31	0.021	0.005
0.501	1	1	1	0.44
1	1	1	1	1
1	0.358	0.214	1	0.089
1	1	1	1	1
1	1	1	1	1
1	1	0.184	0.391	0.009
0.551	1	0.067	0.411	0.008
1	1	1	1	1
0.683	0.316	0.178	0.09	0.003
1	1	0.174	0.391	0.001

1	1	1	1	1
1	0.325	0.138	1	0.067
1	1	1	1	1
0.695	1	0.311	0.09	0.025
1	1	1	1	1
0.194	1	0.15	1	0.03
1	1	0.296	0.105	0.195
0.695	1	0.365	0.09	0.044
0.695	1	0.162	0.09	0.015
1	0.316	0.214	1	0.083
1	1	1	1	1
0.695	1	0.365	0.047	0.024
0.695	1	0.365	0.047	0.024
1	1	1	1	1
