# Supplementary material for: Polymorphism analysis of six selenoprotein genes: support for a selective sweep at the glutathione peroxidase 1 locus (3p21) in Asian populations
Source: BMC Genet. 2006 Dec 11;7:56. doi: 10.1186/1471-2156-7-56 (PMC1769511; doi:10.1186/1471-2156-7-56)
Supplement: Additional File 3 — Genotype Frequencies and Hardy-Weinberg Equilibrium (HWE) Calculations for Single Nucleotide Polymorphisms (SNPs) at the GPX3 Locus. Genotype frequencies and HWE calculations are provided for each of the 4 ethnic subpopulations, AA (n = 24), CA (n = 31), HI (n = 23), and PR (n = 24; n = 23 for GPX1). RS# refers to the SNPs reference cluster ID, a unique SNP ID assigned by dbSNP[77]. Genotype data for identified SNPs have been made available through the SNP500 Cancer database. Where RS# are not yet assigned, the SNP500 Cancer ID# has been provided [63]. Location refers to SNP position relative to the ATG, Stop codon, or Intron/Exon position mapped to the provided genomic reference sequences. Similarly, the Prettybase ID# provides the location of each nucleotide variant/SNP, but refers to the nucleotide sequence position relative to the start of the genomic reference sequence. GPX3 Genotype Frequencies. Genotype frequencies, RS#, SNP location and Hardy-Weinberg equilibrium data is provided for all GPX3 SNPs in this file. [file 1471-2156-7-56-S3.pdf]

Genotype Frequency for Glutathione Peroxidase 3 (GPX3) SNPs

RS#	Location	Prettybase ID#	Frequencies						HWE P Values	
			Variant	AA	CA	HI	PR	Avg	AA	CA
	-2758	173	C:C	1	1	1	0.917	0.98	1	1
			C:T	0	0	0	0.042	0.01		
			T:T	0	0	0	0.042	0.01		
	-2735	196	A:A	0.042	0	0	0	0.01	0.126	1
			A:G	0.083	0	0	0	0.02		
			G:G	0.875	1	1	1	0.97		
rs2042235	-2302	629	C:C	0.2	0.708	0.636	0.708	0.578	0.674	0.152
			C:T	0.45	0.208	0.273	0.208	0.278		
			T:T	0.35	0.083	0.091	0.083	0.144		
rs3763013	-2255	676	C:C	0.684	0.083	0.19	0.167	0.261	0.143	1
			C:T	0.211	0.375	0.333	0.25	0.295		
			T:T	0.105	0.542	0.476	0.583	0.443		
rs1946234	-1006	1925	A:A	0.609	0.8	0.696	0.833	0.74	0.561	1
			A:C	0.304	0.2	0.304	0.167	0.24		
			C:C	0.087	0	0	0	0.02		
rs1946235	-991	1940	C:C	0.087	0	0	0	0.02	0.609	1
			C:T	0.348	0.2	0.304	0.167	0.25		
			T:T	0.565	0.8	0.696	0.833	0.73		
rs1946236	-925	2006	A:A	0.565	0.8	0.696	0.833	0.73	0.609	1
			A:T	0.348	0.2	0.304	0.167	0.25		
			T:T	0.087	0	0	0	0.02		
rs8177402	-914	2017	C:C	0.783	1	0.913	1	0.93	1	1
			C:T	0.217	0	0.087	0	0.07		
			T:T	0	0	0	0	0		
rs17515820	-632	2299	C:C	0.087	0	0	0	0.02	0.561	1
			C:T	0.304	0.2	0.318	0.167	0.242		
			T:T	0.609	0.8	0.682	0.833	0.737		
rs8177407	-582	2349	C:C	0.087	0.276	0	0	0.102	0.561	0.001
			C:T	0.304	0.172	0.318	0.167	0.235		
			T:T	0.609	0.552	0.682	0.833	0.663		
	-491	2440	A:A	0	0	0	0	0	1	1
			A:G	0	0	0.05	0	0.011		
			G:G	1	1	0.95	1	0.989		
rs8177409	-366	2565	A:A	0.609	0.8	0.65	0.833	0.728	0.561	1
			A:T	0.304	0.2	0.35	0.167	0.25		
			T:T	0.087	0	0	0	0.022		
rs6888961	-348	2583	A:A	0.043	0	0	0	0.011	0.31	1
			A:T	0.174	0	0	0.167	0.088		
			T:T	0.783	1	1	0.833	0.901		
rs8177412	-129	2802	C:C	0.087	0	0	0	0.022	0.561	1
			C:T	0.304	0.2	0.35	0.208	0.261		
			T:T	0.609	0.8	0.65	0.792	0.717		
rs8177413	L13L	2969	C:C	0	0	0	0	0	1	1
			C:G	0.043	0	0.083	0.043	0.036		
			G:G	0.957	1	0.917	0.957	0.964		
rs3792798	IV1+1463	4480	A:A	0	0	0.045	0.174	0.05	0.135	0.568
			A:G	0.542	0.323	0.273	0.348	0.37		
			G:G	0.458	0.677	0.682	0.478	0.58		

rs3828599	IV1+1494	4511	A:A	0.25	0.065	0.182	0.217	0.17	0.403	0.678
			A:G	0.625	0.484	0.455	0.348	0.48		
			G:G	0.125	0.452	0.364	0.435	0.35		
	IV1+1588	4605	C:C	0.917	1	1	1	0.98	1	1
			C:T	0.083	0	0	0	0.02		
			T:T	0	0	0	0	0		
rs3792797	IV1+1589	4606	A:A	0.458	0.065	0.091	0.043	0.16	0.083	0.054
			A:C	0.292	0.129	0.318	0.13	0.21		
			C:C	0.25	0.806	0.591	0.826	0.63		
	IV1+1597	4614	A:A	0.042	0	0	0	0.01	0.064	1
			A:G	0.042	0	0	0	0.01		
			G:G	0.917	1	1	1	0.98		
rs8177418	IV1+1640	4657	C:C	0.917	1	0.955	1	0.97	1	1
			C:T	0.083	0	0.045	0	0.03		
			T:T	0	0	0	0	0		
rs4958434	IV1+2268	5285	A:A	0.25	0.087	0.091	0	0.108	0.083	0.052
			A:G	0.292	0.13	0.318	0.042	0.194		
			G:G	0.458	0.783	0.591	0.958	0.699		
rs8177425	IV1+2571	5588	C:C	1	0.968	1	1	0.99	1	1
			C:T	0	0.032	0	0	0.01		
			T:T	0	0	0	0	0		
	IV1+2586	5603	A:A	0	0	0	0	0	1	1
			A:G	0.083	0	0	0	0.021		
			G:G	0.917	1	1	1	0.979		
	IV1+2633	5650	C:C	0.958	1	1	1	0.989	1	1
			C:T	0.042	0	0	0	0.011		
			T:T	0	0	0	0	0		
rs8177426	IV1+2638	5655	A:A	0.333	0.087	0.13	0.042	0.149	0.047	0.052
			A:G	0.292	0.13	0.348	0.125	0.223		
			G:G	0.375	0.783	0.522	0.833	0.628		
rs8177427	IV1+2709	5726	A:A	0.13	0.087	0	0	0.057	0.643	0.052
			A:G	0.391	0.13	0.167	0.042	0.182		
			G:G	0.478	0.783	0.833	0.958	0.761		
	IV1+2712	5729	A:A	0	0	0	0	0	1	1
			A:G	0.042	0	0	0	0.011		
			G:G	0.958	1	1	1	0.989		
	IV2+139	7908	C:C	0.875	1	1	1	0.968	1	1
			C:G	0.125	0	0	0	0.032		
			G:G	0	0	0	0	0		
rs8177437	IV2+333	8102	C:C	0	0	0.067	0.222	0.069	1	1
			C:G	0	0.304	0.2	0.222	0.194		
			G:G	1	0.696	0.733	0.556	0.736		
rs4958874	IV2+341	8110	C:C	0.25	0.304	0.25	0.389	0.301	1	0.379
			C:T	0.5	0.609	0.562	0.278	0.493		
			T:T	0.25	0.087	0.188	0.333	0.205		
rs8177438	IV2+351	8120	C:C	1	0.696	0.867	0.556	0.764	1	1
			C:T	0	0.304	0.133	0.222	0.181		
			T:T	0	0	0	0.222	0.056		
rs869975	IV2+1318	9087	A:A	0	0	0	0.13	0.03	1	1
			A:G	0.083	0.267	0.217	0.261	0.21		
			G:G	0.917	0.733	0.783	0.609	0.76		
rs869976	IV2+1383	9152	A:A	0.5	1	0.913	0.87	0.83	0.352	1

			A:G	0.333	0	0.087	0.087	0.12		
			G:G	0.167	0	0	0.043	0.05		
	IV2+1402	9171	C:C	1	0.839	1	1	0.951	1	0.028
			C:T	0	0.097	0	0	0.029		
			T:T	0	0.065	0	0	0.02		
rs8177444	IV3+66	9361	A:A	0.583	1	0.957	0.913	0.87	1	1
			A:T	0.375	0	0.043	0.043	0.11		
			T:T	0.042	0	0	0.043	0.02		
	IV3+209	9504	A:A	0.5	1	0.909	0.875	0.832	0.352	1
			A:G	0.333	0	0.091	0.083	0.119		
			G:G	0.167	0	0	0.042	0.05		
	IV4+138	9945	A:A	0	0	0	0	0	1	1
			A:G	0.136	0	0	0.042	0.048		
			G:G	0.864	1	1	0.958	0.952		
rs8177447	IV4+364	10171	C:C	0.5	0.75	0.556	0.958	0.702	0.067	0.069
			C:T	0.273	0.15	0.389	0.042	0.202		
			T:T	0.227	0.1	0.056	0	0.095		
rs11548	Stop+92	10498	C:C	0.783	0.7	0.842	0.583	0.719	1	1
			C:T	0.217	0.3	0.158	0.25	0.24		
			T:T	0	0	0	0.167	0.042		
rs2230303	Stop+138	10544	G:G	0.043	0	0	0	0.01	0.519	1
			G:T	0.261	0	0.095	0.042	0.092		
			T:T	0.696	1	0.905	0.958	0.898		
rs8177448	Stop+244	10650	A:A	0	0	0	0	0	1	1
			A:G	0.087	0	0.053	0.042	0.042		
			G:G	0.913	1	0.947	0.958	0.958		
rs8177449	Stop+248	10654	C:C	1	0.839	0.905	0.958	0.919	1	1
			C:T	0	0.161	0.095	0.042	0.081		
			T:T	0	0	0	0	0		
rs2070593	Stop+249	10655	A:A	0	0	0	0.333	0.08	1	0.148
			A:G	0.217	0.484	0.318	0.292	0.34		
			G:G	0.783	0.516	0.682	0.375	0.58		
rs4661	Stop+624	11030	C:C	1	0.903	0.957	1	0.961	1	1
			C:T	0	0.097	0.043	0	0.039		
			T:T	0	0	0	0	0		
SECIS Region		11041-11133								
rs8177454	Stop+948	11354	C:C	0	0	0	0.042	0.01	1	1
			C:G	0	0	0.053	0.042	0.02		
			G:G	1	1	0.947	0.917	0.969		
rs736775	Stop+1657	12063	C:C	0.292	0.355	0.286	0.25	0.3	1	1
			C:T	0.542	0.484	0.333	0.417	0.45		
			T:T	0.167	0.161	0.381	0.333	0.25		
	Stop+1736	12142	A:A	0	0	0	0	0	1	1
			A:G	0	0	0	0.042	0.01		
			G:G	1	1	1	0.958	0.99		
rs2277940	Stop+1786	12192	C:C	0	0	0	0.167	0.04	1	0.568
			C:T	0.125	0.323	0.238	0.25	0.24		
			T:T	0.875	0.677	0.762	0.583	0.72		
	Stop+1844	12250	A:A	0.958	1	1	1	0.99	1	1
			A:C	0.042	0	0	0	0.01		
			C:C	0	0	0	0	0		
	Stop+1851	12257	A:A	0	0	0	0	0	1	1

			A:G	0	0	0	0.042	0.01		
			G:G	1	1	1	0.958	0.99		
rs8177458	Stop+1976	12382	A:A	0	0	0	0	0	1	1
			A:G	0	0.032	0.087	0	0.029		
			G:G	1	0.968	0.913	1	0.971		

HI	PR	Avg
1	0.064	0.015
1	1	0.03
0.271	0.152	0.004
0.335	0.06	0
1	1	1
1	1	1
1	1	1
1	1	1
1	1	1
1	1	0.003
1	1	1
1	1	1
1	1	0.23
1	1	1
1	1	1
0.538	0.355	1

1	0.208	1
1	1	1
0.568	0.214	0
1	1	0.015
1	1	1
0.568	1	0
1	1	1
1	1	1
1	1	1
0.365	0.206	0
1	1	0.018
1	1	1
1	1	1
0.326	0.041	0.019
1	0.069	1
1	0.041	0.034
1	0.128	0.378
1	0.132	0.002

1	1	0.003
1	0.067	0.086
1	0.126	0.002
1	1	1
1	1	0.002
1	0.06	0.257
1	1	0.257
1	1	1
1	1	1
1	0.047	0.421
1	1	1
1	0.064	0.031
0.185	0.433	0.322
1	1	1
1	0.06	0.268
1	1	1
1	1	1

1 1 1
