# Supplementary material for: Polymorphism analysis of six selenoprotein genes: support for a selective sweep at the glutathione peroxidase 1 locus (3p21) in Asian populations
Source: BMC Genet. 2006 Dec 11;7:56. doi: 10.1186/1471-2156-7-56 (PMC1769511; doi:10.1186/1471-2156-7-56)
Supplement: Additional File 4 — Genotype Frequencies and Hardy-Weinberg Equilibrium (HWE) Calculations for Single Nucleotide Polymorphisms (SNPs) at the GPX4 Locus. Genotype frequencies and HWE calculations are provided for each of the 4 ethnic subpopulations, AA (n = 24), CA (n = 31), HI (n = 23), and PR (n = 24; n = 23 for GPX1). RS# refers to the SNPs reference cluster ID, a unique SNP ID assigned by dbSNP[77]. Genotype data for identified SNPs have been made available through the SNP500 Cancer database. Where RS# are not yet assigned, the SNP500 Cancer ID# has been provided [63]. Location refers to SNP position relative to the ATG, Stop codon, or Intron/Exon position mapped to the provided genomic reference sequences. Similarly, the Prettybase ID# provides the location of each nucleotide variant/SNP, but refers to the nucleotide sequence position relative to the start of the genomic reference sequence. GPX4 Genotype Frequencies. Genotype frequencies, RS#, SNP location and Hardy-Weinberg equilibrium data is provided for all GPX4 SNPs in this file. [file 1471-2156-7-56-S4.pdf]

Genotype Frequency For Glutathione Peroxidase 4 (GPX4) SNPs

RS#	Location	Prettybase ID#	Frequencies					HWE P Values		
			Variant	AA	CA	HI	PR	Avg	AA	CA
	-2338	913	A:A	0.958	1	1	1	0.99	1	1
			A:G	0.042	0	0	0	0.01		
			G:G	0	0	0	0	0		
	-2233	1018	C:C	1	0.935	1	1	0.98	1	1
			C:T	0	0.065	0	0	0.02		
			T:T	0	0	0	0	0		
	-2137	1114	G:G	1	0.968	1	1	0.99	1	1
			G:T	0	0.032	0	0	0.01		
			T:T	0	0	0	0	0		
	-2122	1129	A:A	0	0	0	0	0	1	1
			A:G	0	0.032	0	0	0.01		
			G:G	1	0.968	1	1	0.99		
rs757228	-2051	1200	A:A	0.083	0.258	0.304	0.167	0.206	1	0.725
			A:G	0.417	0.548	0.391	0.542	0.48		
			G:G	0.5	0.194	0.304	0.292	0.314		
	-2030	1221	A:A	0.042	0	0	0	0.01	0.126	1
			A:G	0.083	0	0	0	0.02		
			G:G	0.875	1	1	1	0.971		
	-1961	1290	A:A	0.042	0	0	0	0.01	0.298	1
			A:G	0.167	0	0	0	0.039		
			G:G	0.792	1	1	1	0.951		
rs757229	-1929	1322	C:C	0.5	0.267	0.304	0.292	0.337	1	1
			C:G	0.417	0.533	0.391	0.542	0.475		
			G:G	0.083	0.2	0.304	0.167	0.188		
rs757230	-1912	1339	A:A	0.083	0.258	0.304	0.167	0.206	1	0.725
			A:G	0.417	0.548	0.391	0.542	0.48		
			G:G	0.5	0.194	0.304	0.292	0.314		
rs4588110	-1904	1347	A:A	0.083	0.258	0.304	0.167	0.206	1	1
			A:G	0.417	0.516	0.391	0.542	0.471		
			G:G	0.5	0.226	0.304	0.292	0.324		
rs3746166	-1868	1383	C:C	0.083	0.258	0.333	0.167	0.21	1	0.725
			C:T	0.417	0.548	0.381	0.542	0.48		
			T:T	0.5	0.194	0.286	0.292	0.31		
rs3746165	-1832	1419	A:A	0.083	0.29	0.333	0.167	0.22	1	1
			A:G	0.417	0.516	0.381	0.542	0.47		
			G:G	0.5	0.194	0.286	0.292	0.31		
rs757231	-1787	1464	A:A	0.083	0.258	0.35	0.167	0.212	1	0.725
			A:G	0.417	0.548	0.4	0.542	0.485		
			G:G	0.5	0.194	0.25	0.292	0.303		
rs1808194	-1720	1531	A:A	0.5	0.194	0.25	0.292	0.303	1	0.725
			A:G	0.417	0.548	0.4	0.542	0.485		
			G:G	0.083	0.258	0.35	0.167	0.212		
rs8178970	IVS2+30	4518	C:C	0.042	0	0	0	0.011	0.064	1
			C:T	0.042	0.045	0	0	0.023		
			T:T	0.917	0.955	1	1	0.966		
	IVS2+36	4524	C:C	1	0.903	1	1	0.971	1	0.098
			C:G	0	0.065	0	0	0.02		
			G:G	0	0.032	0	0	0.01		

rs4807543	IVS4+299	5315	G:G	0.958	0.897	0.909	0.917	0.919	1	1
			G:T	0.042	0.103	0.091	0.083	0.081		
			T:T	0	0	0	0	0		
	IVS4+322	5338	C:C	1	0.931	0.955	1	0.97	1	1
			C:T	0	0.069	0.045	0	0.03		
			T:T	0	0	0	0	0		
	IVS4+335	5351	C:C	1	1	1	0.958	0.99	1	1
			C:T	0	0	0	0.042	0.01		
			T:T	0	0	0	0	0		
	IVS4+402	5418	A:A	0	0	0	0	0	1	1
			A:G	0.125	0	0	0	0.03		
			G:G	0.875	1	1	1	0.97		
rs8178977	IVS6+17	5685	C:C	0.143	0.042	0.158	0.042	0.091	1	1
			C:G	0.524	0.292	0.158	0.208	0.295		
			G:G	0.333	0.667	0.684	0.75	0.614		
	L193L	5764	A:A	0	0	0	0	0	1	1
			A:G	0	0	0	0.042	0.011		
			G:G	1	1	1	0.958	0.989		
SECIS Region		5814-5907								
rs713041	Stop+44	5823	C:C	0.524	0.375	0.278	0.375	0.391	0.328	1
			C:T	0.333	0.458	0.389	0.458	0.414		
			T:T	0.143	0.167	0.333	0.167	0.195		
	Stop+103	5882	C:C	0.952	1	1	1	0.989	1	1
			C:T	0.048	0	0	0	0.011		
			T:T	0	0	0	0	0		
	Stop+161	5940	A:A	0	0	0	0	0	1	1
			A:G	0.048	0	0.056	0.125	0.057		
			G:G	0.952	1	0.944	0.875	0.943		
rs8178979	Stop+248	6027	C:C	0	0	0	0	0	1	1
			C:T	0.048	0	0.056	0.042	0.034		
			T:T	0.952	1	0.944	0.958	0.966		
rs2075710	Stop+274	6053	C:C	0.19	0.667	0.611	0.75	0.563	1	1
			C:T	0.524	0.292	0.278	0.208	0.322		
			T:T	0.286	0.042	0.111	0.042	0.115		
rs2074451	Stop+464	6243	G:G	0.708	0.29	0.409	0.348	0.43	0.031	0.471
			G:T	0.167	0.419	0.273	0.478	0.34		
			T:T	0.125	0.29	0.318	0.174	0.23		
	Stop+513	6292	A:A	0	0	0	0	0	1	1
			A:G	0.042	0	0	0	0.01		
			G:G	0.958	1	1	1	0.99		
rs2074452	Stop+589	6368	C:C	0.333	0.452	0.571	0.292	0.41	1	0.378
			C:T	0.5	0.516	0.19	0.625	0.47		
			T:T	0.167	0.032	0.238	0.083	0.12		
rs2075711	Stop+764	6543	A:A	0.125	0.3	0.318	0.167	0.23	0.031	0.287
			A:G	0.167	0.4	0.364	0.542	0.37		
			G:G	0.708	0.3	0.318	0.292	0.4		

HI	PR	Avg
1	1	1
1	1	1
1	1	1
1	1	1
0.402	1	0.841
1	1	0.029
1	1	0.073
0.402	1	0.839
0.402	1	0.841
0.402	1	0.689
0.38	1	0.84
0.38	1	0.686
0.395	1	0.84
0.395	1	0.84
1	1	0.035
1	1	0.029

1	1	1
1	1	1
1	1	1
1	1	1
0.027	0.398	0.082
1	1	1
0.37	1	0.188
1	1	1
1	1	1
1	1	1
0.26	0.398	0.103
0.039	1	0.004
1	1	1
0.012	0.217	1
0.218	1	0.023
