# Supplementary material for: Polymorphism analysis of six selenoprotein genes: support for a selective sweep at the glutathione peroxidase 1 locus (3p21) in Asian populations
Source: BMC Genet. 2006 Dec 11;7:56. doi: 10.1186/1471-2156-7-56 (PMC1769511; doi:10.1186/1471-2156-7-56)
Supplement: Additional File 5 — Genotype Frequencies and Hardy-Weinberg Equilibrium (HWE) Calculations for Single Nucleotide Polymorphisms (SNPs) at the SEPP1 Locus. Genotype frequencies and HWE calculations are provided for each of the 4 ethnic subpopulations, AA (n = 24), CA (n = 31), HI (n = 23), and PR (n = 24; n = 23 for GPX1). RS# refers to the SNPs reference cluster ID, a unique SNP ID assigned by dbSNP[77]. Genotype data for identified SNPs have been made available through the SNP500 Cancer database. Where RS# are not yet assigned, the SNP500 Cancer ID# has been provided [63]. Location refers to SNP position relative to the ATG, Stop codon, or Intron/Exon position mapped to the provided genomic reference sequences. Similarly, the Prettybase ID# provides the location of each nucleotide variant/SNP, but refers to the nucleotide sequence position relative to the start of the genomic reference sequence. SEPP1 Genotype Frequencies. Genotype frequencies, RS#, SNP location and Hardy-Weinberg equilibrium data is provided for all SEPP1 SNPs in this file. [file 1471-2156-7-56-S5.pdf]

Genotype Frequency For Selenoprotein P (SEPP1)

RS#	Location	Prettybase ID#	Frequencies						HWE P Values	
			Variant	AA	CA	HI	PR	Avg	AA	CA
rs28919884	-4502	1918	A:A	0.833	1	1	1	0.96	1	1
			A:C	0.167	0	0	0	0.04		
			C:C	0	0	0	0	0		
	-4082	2338	C:C	1	0.933	1	1	0.98	1	1
			C:G	0	0.067	0	0	0.02		
			G:G	0	0	0	0	0		
	-4054	2366	A:A	0	0	0	0	0	1	1
			A:G	0	0	0	0.083	0.02		
			G:G	1	1	1	0.917	0.98		
	-3650	2770	G:G	0	0	0	0	0	1	1
			G:T	0	0.032	0	0	0.011		
			T:T	1	0.968	1	1	0.989		
	K19E	6504	A:A	1	1	0.957	1	0.99	1	1
			A:G	0	0	0.043	0	0.01		
			G:G	0	0	0	0	0		
	IVS3+8	7885	C:C	1	0.968	1	1	0.99	1	1
			C:T	0	0.032	0	0	0.01		
			T:T	0	0	0	0	0		
rs28919897	IVS3+20	7897	C:C	1	1	0.957	1	0.99	1	1
			C:T	0	0	0.043	0	0.01		
			T:T	0	0	0	0	0		
rs230816	IVS4+2809	12926	C:C	0.25	0.387	0.217	0.333	0.304	0.433	0.472
			C:T	0.417	0.419	0.609	0.458	0.471		
			T:T	0.333	0.194	0.174	0.208	0.225		
rs3877899	A234T	13607	A:A	0	0.111	0	0	0.036	1	1
			A:G	0.667	0.556	0.455	0	0.5		
			G:G	0.333	0.333	0.545	1	0.464		
rs7579	Stop+14	14067	A:A	0	0.226	0.095	0.091	0.112	1	0.018
			A:G	0.125	0.258	0.381	0.5	0.306		
			G:G	0.875	0.516	0.524	0.409	0.582		
rs6413428	Stop+98	14151	C:C	0.083	0.065	0	0	0.041	0.676	1
			C:T	0.5	0.387	0.333	0.091	0.337		
			T:T	0.417	0.548	0.667	0.909	0.622		
SECIS Region #1		14292-14391								
SECIS Region #2		14728-14816								
rs12055266	Stop+31177	45230	A:A	0.95	0.593	0.632	0.588	0.687	1	0.072
			A:G	0.05	0.259	0.316	0.294	0.229		
			G:G	0	0.148	0.053	0.118	0.084		
rs12055087	Stop+31192	45245	G:G	0.9	0.577	0.579	0.529	0.646	1	0.024
			G:T	0.1	0.231	0.316	0.353	0.244		
			T:T	0	0.192	0.105	0.118	0.11		
rs2972783	Stop+38404	52457	A:A	0.208	0.414	0.217	0.304	0.293	0.698	0.691
			A:G	0.458	0.414	0.609	0.478	0.485		
			G:G	0.333	0.172	0.174	0.217	0.222		
	Stop+41654	55707	A:A	0	0	0	0	0	1	1
			A:C	0.042	0	0	0	0.01		
			C:C	0.958	1	1	1	0.99		
rs3797311	Stop+43645	57698	C:C	0	0.172	0.13	0.043	0.091	1	0.029

			C:T	0.125	0.241	0.391	0.391	0.283		
			T:T	0.875	0.586	0.478	0.565	0.626		
rs3797310	Stop+43884	57937	A:A	0	0.167	0.13	0.042	0.089	1	0.073
			A:G	0.125	0.267	0.391	0.5	0.317		
			G:G	0.875	0.567	0.478	0.458	0.594		
rs2972994	Stop+44324	58377	C:C	0.208	0.433	0.217	0.348	0.31	0.698	1
			C:T	0.458	0.433	0.609	0.478	0.49		
			T:T	0.333	0.133	0.174	0.174	0.2		
	Stop+44446	58499	G:G	0	0	0	0.042	0.01	1	1
			G:T	0	0.097	0.087	0.125	0.078		
			T:T	1	0.903	0.913	0.833	0.912		
	Stop+44517	58570	C:C	0.217	0.387	0.273	0.364	0.316	0.674	0.723
			C:T	0.435	0.452	0.591	0.455	0.48		
			T:T	0.348	0.161	0.136	0.182	0.204		
rs7730190	Stop+46425	60478	C:C	0	0.192	0.158	0.1	0.118	1	0
			C:T	0.05	0.077	0.263	0.4	0.188		
			T:T	0.95	0.731	0.579	0.5	0.694		
rs1862630	Stop+46581	60634	G:G	0.6	0.5	0.526	0.4	0.506	0.001	0.104
			G:T	0.1	0.308	0.263	0.45	0.282		
			T:T	0.3	0.192	0.211	0.15	0.212		
	Stop+46720	60773	G:G	0.048	0	0	0	0.011	0.144	1
			G:T	0.095	0	0	0	0.023		
			T:T	0.857	1	1	1	0.966		
rs6414899	Stop+46877	60930	C:C	0.381	0.577	0.714	0.944	0.64	0.664	0.55
			C:T	0.524	0.423	0.286	0.056	0.337		
			T:T	0.095	0	0	0	0.023		

HI	PR	Avg
1	1	1
1	1	1
1	1	1
1	1	1
1	1	1
1	1	1
1	1	1
0.421	0.698	0.69
1	1	0.378
1	1	0.038
1	1	1
1	0.525	0.014
0.55	0.573	0.01
0.421	1	0.84
1	1	1
0.643	1	0.047

0.643	0.621	0.177
0.421	1	1
1	0.206	0.207
0.662	1	0.838
0.125	1	0
0.116	1	0.001
1	1	0.035
1	1	0.726
