# Supplementary material for: Polymorphism analysis of six selenoprotein genes: support for a selective sweep at the glutathione peroxidase 1 locus (3p21) in Asian populations
Source: BMC Genet. 2006 Dec 11;7:56. doi: 10.1186/1471-2156-7-56 (PMC1769511; doi:10.1186/1471-2156-7-56)
Supplement: Additional File 6 — Genotype Frequencies and Hardy-Weinberg Equilibrium (HWE) Calculations for Single Nucleotide Polymorphisms (SNPs) at the TXNRD1 Locus. Genotype frequencies and HWE calculations are provided for each of the 4 ethnic subpopulations, AA (n = 24), CA (n = 31), HI (n = 23), and PR (n = 24; n = 23 for GPX1). RS# refers to the SNPs reference cluster ID, a unique SNP ID assigned by dbSNP[77]. Genotype data for identified SNPs have been made available through the SNP500 Cancer database. Where RS# are not yet assigned, the SNP500 Cancer ID# has been provided [63]. Location refers to SNP position relative to the ATG, Stop codon, or Intron/Exon position mapped to the provided genomic reference sequences. Similarly, the Prettybase ID# provides the location of each nucleotide variant/SNP, but refers to the nucleotide sequence position relative to the start of the genomic reference sequence. TXNRD1 Genotype Frequencies. Genotype frequencies, RS#, SNP location and Hardy-Weinberg equilibrium data is provided for all TXNRD1 SNPs in this file. [file 1471-2156-7-56-S6.pdf]

# Genotype Frequencies For Thioredoxin Reductase 1 (TXNRD1) SNPs

| RS# | Location | Prettybase ID# | Frequencies | | | | | |
|---|---|---|---|---|---|---|---|---|
| | | | Variant | AA | CA | HI | PR | Avg |
| | -27135 | 99 | C:C | 0 | 0 | 0 | 0 | 0 |
| | | | C:T | 0.083 | 0.065 | 0.087 | 0 | 0.059 |
| | | | T:T | 0.917 | 0.935 | 0.913 | 1 | 0.941 |
| | -27130 | 104 | G:G | 0.917 | 0.645 | 0.783 | 0.913 | 0.802 |
| | | | G:T | 0.083 | 0.29 | 0.174 | 0.087 | 0.168 |
| | | | T:T | 0 | 0.065 | 0.043 | 0 | 0.03 |
| | -26450 | 784 | A:A | 0.957 | 1 | 0.87 | 1 | 0.96 |
| | | | A:G | 0.043 | 0 | 0.13 | 0 | 0.04 |
| | | | G:G | 0 | 0 | 0 | 0 | 0 |
| | -26409 | 825 | C:C | 0 | 0 | 0 | 0 | 0 |
| | | | C:T | 0 | 0 | 0.043 | 0 | 0.01 |
| | | | T:T | 1 | 1 | 0.957 | 1 | 0.99 |
| | -24961 | 2273 | A:A | 0.083 | 0 | 0 | 0 | 0.021 |
| | | | A:C | 0.083 | 0 | 0 | 0 | 0.021 |
| | | | C:C | 0.833 | 1 | 1 | 1 | 0.957 |
| rs4077561 | -24736 | 2498 | C:C | 0.25 | 0.346 | 0.35 | 0.417 | 0.34 |
| | | | C:T | 0.375 | 0.308 | 0.3 | 0.542 | 0.383 |
| | | | T:T | 0.375 | 0.346 | 0.35 | 0.042 | 0.277 |
| rs10735393 | -24443 | 2791 | G:G | 0.083 | 0.032 | 0 | 0 | 0.031 |
| | | | G:T | 0.292 | 0.387 | 0.368 | 0.25 | 0.327 |
| | | | T:T | 0.625 | 0.581 | 0.632 | 0.75 | 0.643 |
| rs1128446 | -24262 | 2972 | C:C | 0.565 | 0.6 | 0.75 | 0.826 | 0.677 |
| | | | C:G | 0.391 | 0.333 | 0.2 | 0.174 | 0.281 |
| | | | G:G | 0.043 | 0.067 | 0.05 | 0 | 0.042 |
| | -24154 | 3080 | A:A | 0 | 0 | 0 | 0 | 0 |
| | | | A:G | 0.087 | 0 | 0 | 0 | 0.021 |
| | | | G:G | 0.913 | 1 | 1 | 1 | 0.979 |
| | -22591 | 4643 | A:A | 0 | 0 | 0 | 0 | 0 |
| | | | A:G | 0 | 0.033 | 0 | 0 | 0.01 |
| | | | G:G | 1 | 0.967 | 1 | 1 | 0.99 |
| | -22576 | 4658 | C:C | 0.667 | 0.931 | 1 | 0.958 | 0.889 |
| | | | C:G | 0.292 | 0.069 | 0 | 0.042 | 0.101 |
| | | | G:G | 0.042 | 0 | 0 | 0 | 0.01 |
| | -221 | 27013 | A:A | 0.87 | 1 | 1 | 1 | 0.967 |
| | | | A:G | 0.13 | 0 | 0 | 0 | 0.033 |
| | | | G:G | 0 | 0 | 0 | 0 | 0 |
| | -172 | 27062 | G:G | 0 | 0 | 0 | 0 | 0 |
| | | | G:T | 0 | 0.037 | 0 | 0.091 | 0.033 |
| | | | T:T | 1 | 0.963 | 1 | 0.909 | 0.967 |
| | -72 | 27162 | A:A | 0.833 | 1 | 0.895 | 1 | 0.935 |
| | | | A:C | 0.167 | 0 | 0.105 | 0 | 0.065 |
| | | | C:C | 0 | 0 | 0 | 0 | 0 |
| rs5018287 | IVS2+57 | 29281 | A:A | 0.435 | 0.429 | 0.4 | 0.042 | 0.326 |
| | | | A:G | 0.261 | 0.357 | 0.25 | 0.417 | 0.326 |
| | | | G:G | 0.304 | 0.214 | 0.35 | 0.542 | 0.347 |
| | IVS2+85 | 29309 | A:A | 0 | 0.036 | 0 | 0 | 0.011 |
| | | | A:T | 0 | 0.25 | 0.15 | 0 | 0.105 |
| | | | T:T | 1 | 0.714 | 0.85 | 1 | 0.884 |

| | | | | | | | | |
|---|---|---|---|---|---|---|---|---|
| | IVS2+129 | 29353 | A:A | 1 | 1 | 1 | 0.958 | 0.989 |
| | | | A:G | 0 | 0 | 0 | 0.042 | 0.011 |
| | | | G:G | 0 | 0 | 0 | 0 | 0 |
| | IVS2+2414 | 31638 | A:A | 0.958 | 1 | 1 | 1 | 0.99 |
| | | | A:C | 0.042 | 0 | 0 | 0 | 0.01 |
| | | | C:C | 0 | 0 | 0 | 0 | 0 |
| rs4964287 | L55L | 31689 | C:C | 0.375 | 0.452 | 0.478 | 0.75 | 0.51 |
| | | | C:T | 0.417 | 0.419 | 0.217 | 0.25 | 0.333 |
| | | | T:T | 0.208 | 0.129 | 0.304 | 0 | 0.157 |
| | L80L | 31762 | C:C | 1 | 1 | 1 | 0.958 | 0.99 |
| | | | C:T | 0 | 0 | 0 | 0.042 | 0.01 |
| | | | T:T | 0 | 0 | 0 | 0 | 0 |
| | IVS3+72 | 31872 | A:A | 0 | 0 | 0 | 0 | 0 |
| | | | A:G | 0 | 0 | 0.043 | 0 | 0.01 |
| | | | G:G | 1 | 1 | 0.957 | 1 | 0.99 |
| rs17035358 | IVS3+75 | 31875 | A:A | 0 | 0 | 0 | 0 | 0 |
| | | | A:G | 0.083 | 0 | 0 | 0 | 0.02 |
| | | | G:G | 0.917 | 1 | 1 | 1 | 0.98 |
| | IVS3+2942 | 34742 | C:C | 0.864 | 0.968 | 1 | 1 | 0.957 |
| | | | C:T | 0.136 | 0.032 | 0 | 0 | 0.043 |
| | | | T:T | 0 | 0 | 0 | 0 | 0 |
| | IVS4+251 | 35214 | C:C | 0 | 0 | 0 | 0 | 0 |
| | | | C:T | 0 | 0 | 0.045 | 0 | 0.01 |
| | | | T:T | 1 | 1 | 0.955 | 1 | 0.99 |
| | IVS5+1500 | 36991 | C:C | 0 | 0 | 0 | 0 | 0 |
| | | | C:T | 0.083 | 0 | 0 | 0 | 0.02 |
| | | | T:T | 0.917 | 1 | 1 | 1 | 0.98 |
| rs7962256 | IVS9+126 | 43705 | C:C | 0 | 0 | 0 | 0 | 0 |
| | | | C:G | 0.067 | 0.095 | 0.091 | 0.043 | 0.074 |
| | | | G:G | 0.933 | 0.905 | 0.909 | 0.957 | 0.926 |
| | C383C | 47498 | C:C | 0 | 0 | 0 | 0 | 0 |
| | | | C:T | 0.05 | 0 | 0 | 0 | 0.011 |
| | | | T:T | 0.95 | 1 | 1 | 1 | 0.989 |
| rs10778322 | IVS10+158 | 47707 | C:C | 0.381 | 0.467 | 0.565 | 0.708 | 0.531 |
| | | | C:T | 0.381 | 0.433 | 0.13 | 0.292 | 0.316 |
| | | | T:T | 0.238 | 0.1 | 0.304 | 0 | 0.153 |
| | IVS10+199 | 47748 | C:C | 1 | 1 | 1 | 0.957 | 0.99 |
| | | | C:T | 0 | 0 | 0 | 0.043 | 0.01 |
| | | | T:T | 0 | 0 | 0 | 0 | 0 |
| | IVS12+887 | 64057 | G:G | 0 | 0 | 0 | 0 | 0 |
| | | | G:T | 0 | 0.065 | 0 | 0 | 0.021 |
| | | | T:T | 1 | 0.935 | 1 | 1 | 0.979 |
| rs11112003 | IVS12+890 | 64088 | C:C | 0.5 | 0.581 | 0.6 | 0.875 | 0.639 |
| | | | C:T | 0.409 | 0.355 | 0.25 | 0.125 | 0.289 |
| | | | T:T | 0.091 | 0.065 | 0.15 | 0 | 0.072 |
| rs11611385 | Stop+111 | 64440 | C:C | 0.957 | 0.828 | 0.875 | 0.833 | 0.87 |
| | | | C:T | 0.043 | 0.138 | 0.062 | 0.167 | 0.109 |
| | | | T:T | 0 | 0.034 | 0.062 | 0 | 0.022 |
| | Stop+124 | 64453 | C:C | 1 | 1 | 0.895 | 1 | 0.979 |
| | | | C:T | 0 | 0 | 0.105 | 0 | 0.021 |
| | | | T:T | 0 | 0 | 0 | 0 | 0 |
| rs4576900 | Stop+133 | 64462 | C:C | 0.826 | 0.966 | 0.947 | 0.958 | 0.926 |

| | | | | | | | | |
|---|---|---|---|---|---|---|---|---|
| | | | C:T | 0.174 | 0 | 0.053 | 0.042 | 0.063 |
| | | | T:T | 0 | 0.034 | 0 | 0 | 0.011 |
| SECIS Region | | 64545-64640 | | | | | | |
| | Stop+341 | 64670 | C:C | 0.87 | 1 | 1 | 1 | 0.968 |
| | | | C:T | 0.13 | 0 | 0 | 0 | 0.032 |
| | | | T:T | 0 | 0 | 0 | 0 | 0 |
| | Stop+413 | 64742 | C:C | 0.957 | 1 | 1 | 0.958 | 0.979 |
| | | | C:T | 0.043 | 0 | 0 | 0.042 | 0.021 |
| | | | T:T | 0 | 0 | 0 | 0 | 0 |
| | Stop+1075 | 65404 | A:A | 0 | 0 | 0 | 0 | 0 |
| | | | A:G | 0 | 0 | 0.053 | 0 | 0.011 |
| | | | G:G | 1 | 1 | 0.947 | 1 | 0.989 |
| | Stop+1262 | 65591 | C:C | 1 | 0.935 | 1 | 1 | 0.976 |
| | | | C:T | 0 | 0.065 | 0 | 0 | 0.024 |
| | | | T:T | 0 | 0 | 0 | 0 | 0 |
| | Stop+1407 | 65736 | G:G | 0 | 0 | 0 | 0 | 0 |
| | | | G:T | 0 | 0 | 0 | 0.091 | 0.024 |
| | | | T:T | 1 | 1 | 1 | 0.909 | 0.976 |
| | Stop+1653 | 65982 | A:A | 0 | 0 | 0 | 0 | 0 |
| | | | A:C | 0.048 | 0 | 0 | 0 | 0.011 |
| | | | C:C | 0.952 | 1 | 1 | 1 | 0.989 |
| | Stop+2047 | 66376 | A:A | 1 | 0.931 | 1 | 1 | 0.98 |
| | | | A:G | 0 | 0.069 | 0 | 0 | 0.02 |
| | | | G:G | 0 | 0 | 0 | 0 | 0 |
| rs10047589 | Stop+2189 | 66518 | C:C | 0.364 | 0.483 | 0.478 | 0.708 | 0.51 |
| | | | C:T | 0.455 | 0.379 | 0.217 | 0.292 | 0.337 |
| | | | T:T | 0.182 | 0.138 | 0.304 | 0 | 0.153 |
| rs17808524 | Stop+2208 | 66537 | C:C | 0.273 | 0.241 | 0.348 | 0.5 | 0.337 |
| | | | C:G | 0.273 | 0.379 | 0.217 | 0.458 | 0.337 |
| | | | G:G | 0.455 | 0.379 | 0.435 | 0.042 | 0.327 |

HWE P Values

| AA | CA | HI | PR | Avg |
|---|---|---|---|---|
| 1 | 1 | 1 | 1 | 1 |
| 1 | 0.583 | 0.31 | 1 | 0.111 |
| 1 | 1 | 1 | 1 | 1 |
| 1 | 1 | 1 | 1 | 1 |
| 0.021 | 1 | 1 | 1 | 0.001 |
| 0.241 | 0.056 | 0.084 | 0.357 | 0.024 |
| 0.556 | 1 | 1 | 1 | 1 |
| 1 | 0.632 | 0.354 | 1 | 0.507 |
| 1 | 1 | 1 | 1 | 1 |
| 1 | 1 | 1 | 1 | 1 |
| 1 | 1 | 1 | 1 | 0.298 |
| 1 | 1 | 1 | 1 | 1 |
| 1 | 1 | 1 | 1 | 1 |
| 1 | 1 | 1 | 1 | 1 |
| 0.033 | 0.234 | 0.03 | 1 | 0.001 |
| 1 | 0.533 | 1 | 1 | 0.309 |

| | | | | |
|---|---|---|---|---|
| 1 | 1 | 1 | 1 | 1 |
| 1 | 1 | 1 | 1 | 1 |
| 0.672 | 0.7 | 0.01 | 1 | 0.022 |
| 1 | 1 | 1 | 1 | 1 |
| 1 | 1 | 1 | 1 | 1 |
| 1 | 1 | 1 | 1 | 1 |
| 1 | 1 | 1 | 1 | 1 |
| 1 | 1 | 1 | 1 | 1 |
| 1 | 1 | 1 | 1 | 1 |
| 1 | 1 | 1 | 1 | 1 |
| 1 | 1 | 1 | 1 | 1 |
| 0.377 | 1 | 0.001 | 1 | 0.01 |
| 1 | 1 | 1 | 1 | 1 |
| 1 | 1 | 1 | 1 | 1 |
| 1 | 1 | 0.107 | 1 | 0.14 |
| 1 | 0.249 | 0.097 | 1 | 0.076 |
| 1 | 1 | 1 | 1 | 1 |
| 1 | 0.018 | 1 | 1 | 0.142 |

| | | | | |
|---|---|---|---|---|
| 1 | 1 | 1 | 1 | 1 |
| 1 | 1 | 1 | 1 | 1 |
| 1 | 1 | 1 | 1 | 1 |
| 1 | 1 | 1 | 1 | 1 |
| 1 | 1 | 1 | 1 | 1 |
| 1 | 1 | 1 | 1 | 1 |
| 1 | 1 | 1 | 1 | 1 |
| 1 | 0.427 | 0.01 | 1 | 0.035 |
| 0.071 | 0.26 | 0.01 | 0.636 | 0.001 |
