# Supplementary material for: Polymorphism analysis of six selenoprotein genes: support for a selective sweep at the glutathione peroxidase 1 locus (3p21) in Asian populations
Source: BMC Genet. 2006 Dec 11;7:56. doi: 10.1186/1471-2156-7-56 (PMC1769511; doi:10.1186/1471-2156-7-56)
Supplement: Additional File 7 — Most Probable PHASED Haplotypes at the GPX1 Locus Determined Using Only Those SNPs With a Minimum Rare Allele Frequency of ≥ 0.05. Haplotype frequencies are provided for the combined SNP500 DNA population (n = 102), and for each of the 4 ethnic subpopulations, AA (n = 24), CA (n = 31), HI (n = 23), and PR (n = 24; n = 23 for GPX1). RS# refers to the SNPs reference cluster ID, a unique SNP ID assigned by dbSNP[77]. Location refers to SNP position relative to the ATG, Stop codon, or Intron/Exon position mapped to the provided genomic reference sequences. Similarly, the Prettybase ID# provides the location of each nucleotide variant/SNP, but refers to the nucleotide sequence position relative to the start of the genomic reference sequence. For convenience, we have identified a possible subset of SNPs for each gene that are most likely to capture the full variation at the locus in a new, larger data set. These so called haplotype tagged SNPs (htSNPs) are indicated by the word Yes. For reference purposes, an imputed Chimp haplotype was determined by aligning the human locus of interest to the Chimp genome using the Chimp BLAT Search program at the UCSC Genome Bioinformatics Site. GPX1 Haplotype Frequencies. The software program PHASE was used to define haplotypes for the GPX1 locus. Haplotype frequencies for each ethnic population, SNP locations, RS#, and htSNP data are provided. [file 1471-2156-7-56-S7.pdf]

Glutathione Peroxidase 1 (GPX1) Haplotypes

Prettybase	85	2593	2984	3587	3862	4510	5935	6724	7626	All (n=202)		AA (n=48)		CA (n=62)		PR (n=46)		HI (n=46)	
SNP Location	-3538	-1040	-649	-46	P75R	P198L	+1412	+2201	+3103	n	f	n	f	n	f	n	f	n	f
RS#	rs1987628	rs3448	rs3811699	rs1800668		rs1050450	rs6790105	rs11720657											
htSNP	yes				yes	yes	yes		yes										
Chimp	C	G	?	C	C	C	A	C	T										
1	C	G	A	C	C	C	A	C	T	59	0.292	8	0.17	8	0.13	29	0.63	14	0.30
2	C	G	A	C	G	C	A	C	T	36	0.178	2	0.04	13	0.21	10	0.22	11	0.24
3	C	A	A	C	G	C	G	T	C	33	0.163	5	0.10	21	0.34	1	0.02	6	0.13
4	C	A	A	C	C	C	G	T	C	24	0.119	10	0.21	8	0.13			6	0.13
5	T	G	G	T	G	T	A	C	C	17	0.084	3	0.06	9	0.15	1	0.02	4	0.09
6	T	G	G	T	C	T	A	C	C	8	0.040	6	0.13					2	0.04
7	C	G	A	C	C	C	A	C	C	7	0.035	4	0.08			3	0.07		
8	C	G	A	C	C	C	G	C	T	6	0.030	6	0.13						
9	C	G	G	C	C	T	A	C	C	2	0.010	1	0.02					1	0.02
10	T	G	G	C	C	T	A	C	C	2	0.010	1	0.02			1	0.02		
11	C	G	A	C	G	C	A	C	C	2	0.010	1	0.02					1	0.02
12	T	G	G	T	C	C	A	C	C	2	0.010			2	0.03				
13	T	G	A	T	G	T	A	C	C	1	0.005	1	0.02						
14	T	G	G	C	G	T	A	C	C	1	0.005			1	0.02				
15	C	G	A	C	G	C	G	C	T	1	0.005							1	0.02
16	C	A	A	C	C	C	G	T	T	1	0.005					1	0.02		
									Unique Haplotypes	16		12		7		7		9	
