# Supplementary material for: Polymorphism analysis of six selenoprotein genes: support for a selective sweep at the glutathione peroxidase 1 locus (3p21) in Asian populations
Source: BMC Genet. 2006 Dec 11;7:56. doi: 10.1186/1471-2156-7-56 (PMC1769511; doi:10.1186/1471-2156-7-56)
Supplement: Additional File 8 — Most Probable PHASED Haplotypes at the GPX2 Locus Determined Using Only Those SNPs With a Minimum Rare Allele Frequency of ≥ 0.05. Haplotype frequencies are provided for the combined SNP500 DNA population (n = 102), and for each of the 4 ethnic subpopulations, AA (n = 24), CA (n = 31), HI (n = 23), and PR (n = 24; n = 23 for GPX1). RS# refers to the SNPs reference cluster ID, a unique SNP ID assigned by dbSNP[77]. Location refers to SNP position relative to the ATG, Stop codon, or Intron/Exon position mapped to the provided genomic reference sequences. Similarly, the Prettybase ID# provides the location of each nucleotide variant/SNP, but refers to the nucleotide sequence position relative to the start of the genomic reference sequence. For convenience, we have identified a possible subset of SNPs for each gene that are most likely to capture the full variation at the locus in a new, larger data set. These so called haplotype tagged SNPs (htSNPs) are indicated by the word Yes. For reference purposes, an imputed Chimp haplotype was determined by aligning the human locus of interest to the Chimp genome using the Chimp BLAT Search program at the UCSC Genome Bioinformatics Site. GPX2 Haplotype Frequencies. The software program PHASE was used to define haplotypes for the GPX2 locus. Haplotype frequencies for each ethnic population, SNP locations, RS#, and htSNP data are provided. [file 1471-2156-7-56-S8.pdf]

Glutathione Peroxidase 2 (GPX2) Haplotypes

Prettybase	168	538	628	947	1246	1899	2445	2457	3312	3350	3645	3937	4473	5286	5446	6724	7064	7514	7547	8004	8330	8360	8468	8542	All (n=204)		AA (n=48)		CA (n=62)		PR (n=48)		HI (n=46)	
SNP Location	-2834	-2464	-2374	-2055	-1756	-1103	-557	-545	IVS	IVS	IVS	IVS	IVS	IVS	IVS	+484	+824	+1274	+1307	+1764	+2090	+2120	+2228	+2302	n	f	n	f	n	f	n	f	n	f
									+88	+126	+421	+713	+1249	+2062	+2222																			
RS#	rs2277502	rs3742599		rs3825644	rs17880531	rs17880145	rs17880939	rs17093568	rs17882875	rs17880758	rs17880303	rs17884367	rs17881779	rs4902346	rs2071566	rs17884597	rs17883891	rs17881498	rs17880380	rs17881134	rs17886858	rs17883358	rs10132858	rs4902345										
htSNP	yes	yes	yes	yes		yes		yes	yes	yes		yes				yes			yes		yes													
Chimp	G	G	C	A	G	A	A	G	G	G	T	T	C	T	A	C	C	C	C	G	G	C	T	G										
1	G	G	C	A	G	G	A	G	G	G	T	C	T	T	G	C	C	T	C	T	G	C	T	A	94	0.461	9	0.19	45	0.73	17	0.35	23	0.50
2	G	G	C	A	G	A	A	T	G	G	T	T	C	T	A	C	C	T	C	T	G	C	T	A	22	0.108					22	0.46		
3	G	G	T	C	G	G	A	G	G	G	T	T	C	T	A	C	C	T	C	T	G	C	T	A	10	0.049	1	0.02					2	0.04
4	A	T	C	A	A	A	G	G	T	G	C	T	C	C	A	A	T	C	T	G	C	T	C	G	10	0.049			5	0.08	2	0.04	3	0.07
5	G	G	C	A	G	G	A	G	G	G	T	T	C	T	A	C	C	T	C	T	G	C	T	A	7	0.034	1	0.02	4	0.06	1	0.02	1	0.02
6	G	G	C	A	G	A	A	G	G	G	T	T	C	T	A	C	C	T	C	T	G	C	T	A	6	0.029	1	0.02						0.00
7	G	G	C	A	G	A	A	G	G	G	T	T	C	C	A	C	T	C	T	G	C	C	C	G	5	0.025	3	0.06	1	0.02	1	0.02		
8	G	G	C	A	G	G	A	G	G	A	T	T	C	C	A	C	T	C	T	G	C	C	C	G	5	0.025	3	0.06					2	0.04
9	G	T	C	A	G	A	A	G	G	G	T	T	C	C	A	C	T	C	T	G	C	C	C	G	5	0.025	3	0.06					2	0.04
10	G	G	C	A	G	G	A	G	G	G	T	T	C	C	A	C	T	C	T	G	C	C	C	G	4	0.020	4	0.08						
11	G	T	C	A	G	G	A	G	G	A	T	T	C	C	A	C	T	C	T	G	C	C	C	G	4	0.020	1	0.02	1	0.02			2	0.04
12	G	G	C	C	G	A	A	G	G	G	T	T	C	C	A	C	T	C	T	G	C	C	C	G	3	0.015	2	0.04			1	0.02		
13	G	G	C	C	G	A	A	G	G	G	T	C	T	T	G	C	C	T	C	T	G	C	T	A	3	0.015			1	0.02			2	0.04
14	G	G	C	A	G	A	A	G	T	G	T	T	C	C	A	C	T	T	T	G	C	C	C	G	3	0.015					3	0.06		
15	G	G	C	A	G	G	A	G	G	A	T	T	C	C	A	C	T	T	T	G	C	C	C	G	2	0.010	1	0.02					1	0.02
16	G	T	C	A	G	A	A	G	G	A	T	T	C	C	A	C	T	C	T	G	C	C	C	G	2	0.010	2	0.04						
17	G	G	C	A	G	G	A	G	G	G	T	T	C	C	A	C	T	T	C	G	C	C	C	G	1	0.005	1	0.02						
18	G	G	C	A	G	G	A	G	G	A	T	T	C	C	A	C	T	T	C	G	C	C	C	G	1	0.005	1	0.02						
19	G	G	C	C	G	A	A	G	G	G	T	T	C	C	A	C	T	T	T	G	C	C	C	G	1	0.005	1	0.02						
20	G	G	T	C	G	G	A	G	G	G	T	C	C	T	A	C	C	T	C	T	G	C	T	A	1	0.005	1	0.02						
21	G	G	C	C	G	G	A	G	G	G	T	T	C	C	A	C	T	T	C	G	C	C	C	G	1	0.005	1	0.02						
22	A	T	C	A	A	A	G	G	T	G	C	T	C	T	G	A	T	C	T	G	C	T	C	G	1	0.005			1	0.02				
23	G	G	C	A	G	A	A	T	G	G	T	T	C	T	G	C	C	T	C	T	G	C	T	A	1	0.005			1	0.02				
24	G	T	C	A	A	A	G	G	T	G	C	T	C	T	G	A	T	C	T	G	C	T	C	G	1	0.005			1	0.02				
25	G	T	C	A	G	G	A	G	G	A	T	T	C	T	A	C	T	C	T	G	C	C	C	G	1	0.005			1	0.02				
26	G	G	C	A	G	G	A	G	G	G	T	T	C	T	G	C	T	C	T	G	C	C	C	G	1	0.005			1	0.02				
27	G	G	C	A	G	G	A	G	G	G	T	C	T	T	G	C	C	T	C	T	C	C	T	A	1	0.005							1	0.02
28	A	T	C	A	A	A	G	G	T	G	C	T	C	C	G	A	T	C	T	G	C	T	C	G	1	0.005							1	0.02
29	G	G	C	A	G	G	A	G	G	G	T	C	T	C	G	C	C	T	C	T	G	C	T	A	1	0.005							1	0.02
30	G	G	C	A	G	G	A	G	T	G	T	T	C	C	A	C	T	C	T	G	C	C	C	G	1	0.005							1	0.02
31	G	T	C	A	G	G	A	G	G	G	T	C	T	T	G	C	C	T	C	T	G	C	T	A	1	0.005							1	0.02
32	G	T	C	A	A	A	G	G	T	G	C	T	T	C	A	A	T	C	T	G	C	T	C	G	1	0.005							1	0.02
33	G	G	C	C	G	G	A	G	G	G	T	T	C	C	A	C	C	C	T	G	C	C	C	G	1	0.005							1	0.02
34	G	G	C	A	G	A	A	T	G	G	T	T	C	T	A	C	C	C	C	T	G	C	T	A	1	0.005							1	0.02
35	A	T	C	A	A	A	G	G	T	G	C	T	C	C	A	A	T	C	T	G	C	T	T	A	1	0.005					1	0.02		
																						Unique Haplotypes			35		17		11		8		17	
