# Supplementary material for: Polymorphism analysis of six selenoprotein genes: support for a selective sweep at the glutathione peroxidase 1 locus (3p21) in Asian populations
Source: BMC Genet. 2006 Dec 11;7:56. doi: 10.1186/1471-2156-7-56 (PMC1769511; doi:10.1186/1471-2156-7-56)
Supplement: Additional File 9 — Most Probable PHASED Haplotypes at the GPX3 Locus Determined Using Only Those SNPs With a Minimum Rare Allele Frequency of ≥ 0.05. Haplotype frequencies are provided for the combined SNP500 DNA population (n = 102), and for each of the 4 ethnic subpopulations, AA (n = 24), CA (n = 31), HI (n = 23), and PR (n = 24; n = 23 for GPX1). RS# refers to the SNPs reference cluster ID, a unique SNP ID assigned by dbSNP[77]. Location refers to SNP position relative to the ATG, Stop codon, or Intron/Exon position mapped to the provided genomic reference sequences. Similarly, the Prettybase ID# provides the location of each nucleotide variant/SNP, but refers to the nucleotide sequence position relative to the start of the genomic reference sequence. For convenience, we have identified a possible subset of SNPs for each gene that are most likely to capture the full variation at the locus in a new, larger data set. These so called haplotype tagged SNPs (htSNPs) are indicated by the word Yes. For reference purposes, an imputed Chimp haplotype was determined by aligning the human locus of interest to the Chimp genome using the Chimp BLAT Search program at the UCSC Genome Bioinformatics Site. GPX3 Haplotype Frequencies. The software program PHASE was used to define haplotypes for the GPX3 locus. Haplotype frequencies for each ethnic population, SNP locations, RS#, and htSNP data are provided. [file 1471-2156-7-56-S9.pdf]

Glutathione Peroxidase 3 (GPX3) Haplotypes

Prettybase	629	676	1925	1940	2006	2299	2349	2565	2583	2802	4480	4511	4606	5285	5655	5726	8102	8110	8120	9087	9152	9361	9504	10171	10498	10544	10655	12063	12192	All (n=204)		AA (n=48)		CA (n=62)		PR (n=48)		HI (n=46)	
SNP Location	-2302	-2255	-1006	-991	-925	-632	-582	-366	-348	-129	IVS1 +1463	IVS1 +1494	IVS1 +1589	IVS1 +2268	IVS1 +2638	IVS1 +2709	IVS2 +333	IVS2 +341	IVS2 +351	IVS2 +1318	IVS2 +1383	IVS3 +66	IVS3 +209	IVS4 +364	+92	+138	+249	+1657	+1786	n	f	n	f	n	f	n	f	n	f
RS#	rs2042235	rs3763013	rs1946234	rs1946235	rs1946236	rs17515820	rs8177407	rs8177409	rs6888961	rs8177412	rs3792798	rs3828599	rs3792797	rs4958434	rs8177426	rs8177427	rs8177437	rs4958874	rs8177438	rs869975	rs869976	rs8177444		rs8177447	rs11548	rs2230303	rs2070593	rs736775	rs2277940										
htSNP	yes	yes				yes	yes		yes	yes	yes	yes	yes		yes	yes	yes	yes	yes	yes		yes	yes	yes	yes	yes	yes	yes											
Chimp	T	C	A	C	A	C	C	T	T	C	G	G	A	G	A	G	G	C	C	G	G	T	G	C	C	T	A	T	T										
1	C	T	A	T	A	T	T	A	T	T	G	G	C	G	G	G	G	C	C	G	A	A	A	C	C	T	G	C	T	62	0.304	2	0.04	25	0.40	18	0.38	17	0.37
2	C	T	A	T	A	T	T	A	T	T	A	A	C	G	G	G	C	T	T	A	A	A	A	C	T	T	A	T	C	25	0.123	2	0.04	7	0.11	12	0.25	4	0.09
3	T	C	A	T	A	T	T	A	T	T	G	A	A	A	A	A	G	T	C	G	A	A	A	T	C	T	G	T	T	11	0.054	4	0.08	1	0.02	1	0.02	5	0.11
4	T	C	A	T	A	T	T	A	T	T	G	A	A	A	A	A	G	T	C	G	A	A	A	T	C	T	G	C	T	7	0.034	7	0.15						
5	C	C	C	C	T	C	C	T	T	C	G	G	C	G	G	G	G	T	C	G	A	A	A	C	C	T	A	T	T	7	0.034			5	0.08			2	0.04
6	C	C	C	C	T	C	C	T	T	C	G	A	A	A	A	A	G	T	C	G	A	A	A	T	C	T	G	T	T	6	0.029	3	0.063					3	0.07
7	C	T	A	T	A	T	C	A	T	T	G	G	C	G	G	G	G	C	C	G	A	A	A	C	C	T	G	C	T	6	0.029			6	0.10				
8	T	C	A	T	A	T	T	A	T	T	G	G	C	G	G	G	G	C	C	G	A	A	A	C	C	T	G	C	T	5	0.025	2	0.04	2	0.03			1	0.02
9	T	C	A	T	A	T	C	A	T	T	G	A	A	A	A	A	G	T	C	G	A	A	A	T	C	T	G	T	T	5	0.025			5	0.08				
10	T	C	A	T	A	T	T	A	T	T	G	G	C	G	G	G	G	C	C	G	G	T	G	C	C	T	G	C	T	3	0.015	3	0.063						
11	C	C	C	C	T	C	C	T	T	C	G	G	C	G	G	G	G	C	C	G	A	A	A	C	C	T	G	T	T	3	0.015	2	0.04					1	0.02
12	T	C	A	T	A	T	T	A	T	T	G	G	A	A	A	G	G	T	C	G	A	A	A	T	C	T	G	C	T	3	0.015	2	0.04					1	0.02
13	T	C	A	T	A	T	T	A	A	T	G	G	C	G	G	G	G	C	C	G	A	A	A	C	C	T	G	C	T	3	0.015	2	0.04			1	0.02		
14	C	T	A	T	A	T	T	A	A	T	G	G	A	G	G	G	G	T	T	G	G	A	G	C	C	G	G	C	T	3	0.015	3	0.063						
15	T	C	A	T	A	T	T	A	T	T	G	G	A	G	A	G	G	C	C	G	G	T	G	C	C	T	A	T	T	3	0.015					3	0.06		
16	C	T	A	T	A	T	T	A	T	T	A	A	C	G	G	G	G	T	C	G	A	A	A	C	T	T	A	T	T	2	0.010	2	0.04						
17	C	T	A	T	A	T	T	A	T	T	A	A	A	G	G	G	G	C	C	G	G	T	G	C	C	G	G	C	T	2	0.010	2	0.04						
18	C	C	C	C	T	C	C	T	T	C	G	G	C	G	A	G	G	C	C	G	G	A	G	C	C	T	G	T	T	2	0.010	2	0.04						
19	T	T	A	T	A	T	T	A	T	T	G	A	C	A	A	G	G	T	C	G	A	A	A	T	C	T	G	T	T	2	0.010			1	0.02			1	0.02
20	C	T	A	T	A	T	T	A	T	T	G	G	C	G	G	G	G	C	C	G	A	A	A	C	C	T	G	T	C	2	0.010			2	0.03				
21	T	C	A	T	A	T	C	A	T	T	G	G	C	G	G	G	G	C	C	G	A	A	A	C	C	T	G	C	T	2	0.010			2	0.03				
22	C	T	A	T	A	T	C	A	T	T	G	A	A	A	A	A	G	T	C	G	A	A	A	T	C	T	G	T	T	2	0.010			2	0.03				
23	C	T	A	T	A	T	T	A	T	T	A	A	C	G	G	G	C	T	C	G	A	A	A	C	C	T	A	T	T	2	0.010					1	0.02	1	0.02
24	C	T	A	T	A	T	T	A	T	T	G	G	C	G	G	G	G	C	C	G	A	A	A	C	C	T	G	T	T	2	0.010					1	0.02	1	0.02
25	T	C	A	T	A	T	T	A	A	C	G	G	C	G	G	G	G	T	C	G	A	A	A	C	C	T	G	C	T	2	0.010					1	0.02	1	0.02
26	C	T	A	T	A	T	T	A	T	T	G	G	C	A	A	G	G	C	C	G	A	A	A	C	C	T	G	C	T	2	0.010							2	0.04
27	C	C	A	T	A	T	T	A	T	T	A	A	A	G	A	G	G	T	C	G	G	A	G	C	C	G	G	T	T	2	0.010					1	0.02	1	0.02
28	C	C	A	T	A	T	C	T	T	C	G	G	C	G	G	G	G	T	C	G	A	A	A	C	T	T	A	T	C	2	0.010					2	0.04		
29	C	T	C	C	T	C	T	A	T	T	A	A	C	G	G	G	C	T	T	A	A	A	A	C	C	T	A	T	T	2	0.010					2	0.04		
30	T	C	A	C	A	T	C	T	A	C	G	G	C	G	G	G	G	T	C	G	A	A	A	C	C	T	A	C	T	2	0.010					2	0.04		
31	C	C	C	T	T	C	T	A	T	T	G	G	C	G	G	G	G	C	C	G	A	A	A	C	C	T	G	T	T	2	0.010					2	0.04		
32	T	C	A	T	A	T	T	A	T	T	A	A	A	A	A	A	G	T	C	G	A	A	A	T	C	T	G	T	T	1	0.005	1	0.02						
33	C	C	C	C	T	C	C	T	T	C	G	A	A	A	A	G	G	T	C	G	A	A	A	T	C	T	G	T	T	1	0.005	1	0.02						
34	T	C	A	T	A	T	T	A	A	T	A	A	A	G	G	G	G	C	C	G	G	T	G	C	C	G	G	T	T	1	0.005	1	0.02						
35	C	C	C	C	T	C	C	T	T	C	A	A	A	G	G	G	G	C	C	G	G	T	G	C	C	T	G	C	T	1	0.005	1	0.02						
36	T	C	C	C	T	T	T	A	T	T	G	G	A	A	A	A	G	T	C	G	G	T	G	C	C	T	A	T	T	1	0.005	1	0.02						
37	C	C	A	C	T	C	C	T	T	C	G	G	C	G	G	G	G	C	C	G	A	A	A	C	C	T	G	C	T	1	0.005	1	0.02						
38	C	T	A	T	A	T	T	A	T	T	A	A	C	G	G	G	G	T	C	G	A	A	A	C	T	T	A	T	C	1	0.005	1	0.02						
39	T	C	A	T	A	T	T	A	T	T	A	G	A	G	A	G	G	C	C	G	G	T	G	C	C	T	G	T	T	1	0.005	1	0.02						
40	C	C	C	C	T	C	C	T	T	C	A	A	A	G	A	G	G	C	C	G	G	T	G	C	C	G	G	C	T	1	0.005	1	0.02						
41	C	C	C	C	T	C	C	T	T	C	A	A	A	G	G	G	G	C	C	G	G	T	G	C	C	G	G	C	T	1	0.005	1	0.02						
42	C	T	C	C	T	C	C	T	T	C	G	G	C	G	G	G	G	T	C	G	A	A	A	C	C	T	A	T	T	1	0.005			1	0.02				
43	C	T	A	T	A	T	T	A	T	T	A	A	C	G	G	G	C	T	T	A	A	A	A	C	T	T	A	C	T	1	0.005			1	0.02				
44	C	T	A	T	A	T	T	A	T	T	A	A	C	G	G	G	G	C	C	G	A	A	A	C	C	T	G	C	T	1	0.005			1	0.02				
45	C	T	A	T	A	T	C	A	T	T	A	A	C	G	G	G	C	T	T	A	A	A	A	C	T	T	A	T	C	1	0.005			1	0.02				
46	T	C	A	T	A	T	T	A	T	T	G	G	C	G	G	G	G	C	C	G	A	A	A	C	C	T	G	T	T	1	0.005							1	0.02
47	C	T	A	T	A	T	T	A	T	T	A	A	C	G	G	G	C	T	C	A	A	A	A	C	C	T	A	T	T	1	0.005							1	0.02
48	C	T	A	T	A	T	T	A	T	T	G	G	C	G	G	G	C	C	C	G	A	A	A	C	C	T	G	T	C	1	0.005							1	0.02
49	C	C	C	C	T	C	C	T	T	C	G	A	C	A	A	A	G	T	C	G	A	A	A	T	C	T	G	T	T	1	0.005							1	0.02
50	T	C	A	T	A	T	T	A	T	T	A	A	A	G	G	G	G	C	C	G	G	T	G	C	C	G	G	T	T	1	0.005							1	0.02
51	T	C	A	T	A	T	T	A	T	T	G	A	C	G	G	G	G	C	C	G	A	A	A	C	C	T	A	T	T	1	0.005					1	0.02		
Unique Haplotypes																														51		24		15		14		19	
