# Supplementary material for: Polymorphism analysis of six selenoprotein genes: support for a selective sweep at the glutathione peroxidase 1 locus (3p21) in Asian populations
Source: BMC Genet. 2006 Dec 11;7:56. doi: 10.1186/1471-2156-7-56 (PMC1769511; doi:10.1186/1471-2156-7-56)
Supplement: Additional File 10 — Most Probable PHASED Haplotypes at the GPX4 Locus Determined Using Only Those SNPs With a Minimum Rare Allele Frequency of ≥ 0.05. Haplotype frequencies are provided for the combined SNP500 DNA population (n = 102), and for each of the 4 ethnic subpopulations, AA (n = 24), CA (n = 31), HI (n = 23), and PR (n = 24; n = 23 for GPX1). RS# refers to the SNPs reference cluster ID, a unique SNP ID assigned by dbSNP[77]. Location refers to SNP position relative to the ATG, Stop codon, or Intron/Exon position mapped to the provided genomic reference sequences. Similarly, the Prettybase ID# provides the location of each nucleotide variant/SNP, but refers to the nucleotide sequence position relative to the start of the genomic reference sequence. For convenience, we have identified a possible subset of SNPs for each gene that are most likely to capture the full variation at the locus in a new, larger data set. These so called haplotype tagged SNPs (htSNPs) are indicated by the word Yes. For reference purposes, an imputed Chimp haplotype was determined by aligning the human locus of interest to the Chimp genome using the Chimp BLAT Search program at the UCSC Genome Bioinformatics Site. GPX4 Haplotype Frequencies. The software program PHASE was used to define haplotypes for the GPX4 locus. Haplotype frequencies for each ethnic population, SNP locations, RS#, and htSNP data are provided. [file 1471-2156-7-56-S10.pdf]

Glutathione Peroxidase 4 (GPX4) Haplotypes

Prettybase	1200	1322	1339	1347	1383	1419	1464	1531	5685	5823	6053	6243	6368	6543	ALL (n=204)		AA (n=48)		CA (n=62)		PR (n=48)		HI (n=46)	
SNP Location	-2051	-1929	-1912	-1904	-1868	-1832	-1787	-1720	IVS6 +17	+44	+274	+464	+589	+764	n	f	n	f	n	f	n	f	n	f
RS#	rs757228	rs757229	rs757230	rs4588110	rs3746166	rs3746165	rs757231	rs1808194	rs8178977	rs713041	rs207571	rs2074451	rs2074452	rs2075711										
htSNP			yes						yes	yes	yes		yes	yes										
Chimp	G	G	T	A	T	G	G	G	G	C	C	G	C	G										
1	A	G	A	A	C	A	A	G	G	T	C	T	C	A	65	0.319	6	0.13	21	0.34	19	0.41		
2	G	C	G	G	T	G	G	A	G	C	C	G	T	G	49	0.240	2	0.04	15	0.24	14	0.30	18	0.39
3	G	C	G	G	T	G	G	A	C	C	T	G	C	G	27	0.132	5	0.10	9	0.15	7	0.15	6	0.13
4	G	C	G	G	T	G	G	A	G	C	T	G	C	G	14	0.069	10	0.21	2	0.03	1	0.02	1	0.02
5	G	C	G	G	T	G	G	A	C	C	T	G	T	G	9	0.044	9	0.19						
6	A	G	A	A	C	A	A	G	C	C	C	G	T	G	7	0.034	4	0.08	2	0.03			1	0.02
7	A	G	A	A	C	A	A	G	G	C	C	T	C	A	6	0.029			4	0.06	1	0.02	1	0.02
8	G	C	G	G	T	G	G	A	G	C	C	G	C	G	4	0.020	2	0.04	1	0.02			1	0.02
9	A	G	A	A	C	A	A	G	C	C	T	G	T	G	4	0.020	2	0.04			2	0.04		
10	G	C	A	A	T	G	A	A	G	T	C	T	C	A	3	0.015	2	0.04	1	0.02				
11	A	G	G	G	C	A	G	G	G	T	C	T	C	A	3	0.015	2	0.04	1	0.02				
12	G	C	G	G	T	G	G	A	G	T	C	G	T	G	3	0.015	2	0.04						
13	A	C	A	A	C	A	A	G	G	C	C	T	C	A	2	0.010			2	0.03				
14	A	C	A	A	C	A	A	G	G	T	C	T	C	A	2	0.010			2	0.03				
15	G	C	G	G	T	G	G	A	G	C	C	G	C	A	2	0.010					1	0.02	1	0.02
16	G	C	G	G	T	G	G	A	G	C	T	G	T	G	1	0.005	1	0.02						
17	G	C	G	G	T	G	G	A	G	T	C	G	C	G	1	0.005	1	0.02						
18	G	C	G	G	T	A	G	A	G	C	C	G	T	G	1	0.005			1	0.02			19	0.41
19	A	G	A	G	C	A	A	G	C	C	T	G	C	G	1	0.005			1	0.02				
20	A	G	A	A	C	A	A	G	C	T	C	T	C	A	1	0.005					1	0.02		
												Unique Haplotypes			20		13		13		8		8	
