# Supplementary material for: Polymorphism analysis of six selenoprotein genes: support for a selective sweep at the glutathione peroxidase 1 locus (3p21) in Asian populations
Source: BMC Genet. 2006 Dec 11;7:56. doi: 10.1186/1471-2156-7-56 (PMC1769511; doi:10.1186/1471-2156-7-56)
Supplement: Additional File 11 — Most Probable PHASED Haplotypes at the SEPP1 Locus Determined Using Only Those SNPs With a Minimum Rare Allele Frequency of ≥ 0.05. Haplotype frequencies are provided for the combined SNP500 DNA population (n = 102), and for each of the 4 ethnic subpopulations, AA (n = 24), CA (n = 31), HI (n = 23), and PR (n = 24; n = 23 for GPX1). RS# refers to the SNPs reference cluster ID, a unique SNP ID assigned by dbSNP[77]. Location refers to SNP position relative to the ATG, Stop codon, or Intron/Exon position mapped to the provided genomic reference sequences. Similarly, the Prettybase ID# provides the location of each nucleotide variant/SNP, but refers to the nucleotide sequence position relative to the start of the genomic reference sequence. For convenience, we have identified a possible subset of SNPs for each gene that are most likely to capture the full variation at the locus in a new, larger data set. These so called haplotype tagged SNPs (htSNPs) are indicated by the word Yes. For reference purposes, an imputed Chimp haplotype was determined by aligning the human locus of interest to the Chimp genome using the Chimp BLAT Search program at the UCSC Genome Bioinformatics Site. SEPP1 Haplotype Frequencies. The software program PHASE was used to define haplotypes for the SEPP1 locus. Haplotype frequencies for each ethnic population, SNP locations, RS#, and htSNP data are provided. [file 1471-2156-7-56-S11.pdf]

Selenoprotein P (SEPP1) Haplotypes

Prettybase	12926	13607	14067	14151	45230	45245	52457	57698	57937	58377	58570	60478	60634	60930	ALL (n=204)		AA (n=48)		CA (n=62)		PR (n=48)		HI (n=46)	
SNP Location	IVS4 +2809	A234T	+14	+98	+31177	+31192	+38404	+43645	+43884	+44324	+44517	+46425	+46581	+46877	n	f	n	f	n	f	n	f	n	f
RS#	rs230816	rs3877899	rs7579	rs6413428	rs12055266	rs12055087	rs2972783	rs3797311	rs3797310	rs2972994		rs7730190	rs1862630	rs6414899										
htSNP		yes			yes		yes		yes				yes											
Chimp	C	G	G	T	A	G	A	T	G	C	C	T	G	C										
1	T	G	G	T	A	G	G	T	G	T	T	T	T	C	74	0.363	20	0.42	20	0.32	20	0.42	14	0.30
2	C	A	G	C	A	G	A	T	G	C	C	T	G	T	37	0.181	14	0.29	15	0.24	2	0.04	6	0.13
3	C	G	A	T	G	T	A	C	A	C	C	C	G	C	34	0.167			12	0.19	11	0.23	11	0.24
4	T	G	G	T	A	G	G	T	G	T	T	T	G	C	14	0.069	6	0.13	2	0.03			6	0.13
5	C	G	G	T	A	G	A	T	G	C	C	T	G	C	13	0.064	2	0.04			10	0.21	1	0.02
6	C	A	A	T	G	T	A	T	G	C	C	C	G	C	5	0.025			4	0.06	1	0.02		
7	C	G	A	T	G	T	A	C	A	C	C	T	G	C	4	0.020			2	0.03	1	0.02	1	0.02
8	C	G	A	T	A	T	A	C	A	C	C	T	G	C	3	0.015	2	0.04	1	0.02				
9	C	G	A	T	A	T	A	C	A	C	C	C	G	C	3	0.015			1	0.02			2	0.04
10	C	A	G	C	A	G	A	T	G	C	C	T	G	C	3	0.015			1	0.02			2	0.04
11	C	G	G	C	A	G	A	T	G	C	C	T	G	T	2	0.010	2	0.04						
12	T	G	G	T	A	G	G	T	G	T	C	T	T	C	2	0.010							2	0.04
13	C	G	G	T	A	G	G	T	G	T	T	T	G	C	1	0.005	1	0.02						
14	C	G	A	T	G	G	A	C	A	C	C	C	G	C	1	0.005	1	0.02						
15	T	G	G	T	A	G	A	T	G	T	T	T	G	C	1	0.005			1	0.02				
16	T	G	G	T	A	G	G	T	G	C	T	T	T	C	1	0.005			1	0.02				
17	T	G	A	T	G	T	A	C	A	C	C	C	G	C	1	0.005			1	0.02				
18	C	G	A	T	G	T	A	T	A	C	C	C	G	C	1	0.005			1	0.02				
19	C	G	G	T	G	T	A	C	A	C	C	C	G	C	1	0.005							1	0.02
20	C	G	A	T	G	G	A	T	A	C	C	C	G	C	1	0.005					1	0.02		
21	T	G	G	T	A	T	G	T	G	T	T	T	T	C	1	0.005					1	0.02		
22	C	G	A	T	A	T	A	T	A	C	C	C	G	C	1	0.005					1	0.02		
												Unique Haplotypes			22		8		13		9		10	
