# Supplementary material for: Polymorphism analysis of six selenoprotein genes: support for a selective sweep at the glutathione peroxidase 1 locus (3p21) in Asian populations
Source: BMC Genet. 2006 Dec 11;7:56. doi: 10.1186/1471-2156-7-56 (PMC1769511; doi:10.1186/1471-2156-7-56)
Supplement: Additional File 12 — Most Probable PHASED Haplotypes at the TXNRD1 Locus Determined Using Only Those SNPs With a Minimum Rare Allele Frequency of ≥ 0.05. Haplotype frequencies are provided for the combined SNP500 DNA population (n = 102), and for each of the 4 ethnic subpopulations, AA (n = 24), CA (n = 31), HI (n = 23), and PR (n = 24; n = 23 for GPX1). RS# refers to the SNPs reference cluster ID, a unique SNP ID assigned by dbSNP[77]. Location refers to SNP position relative to the ATG, Stop codon, or Intron/Exon position mapped to the provided genomic reference sequences. Similarly, the Prettybase ID# provides the location of each nucleotide variant/SNP, but refers to the nucleotide sequence position relative to the start of the genomic reference sequence. For convenience, we have identified a possible subset of SNPs for each gene that are most likely to capture the full variation at the locus in a new, larger data set. These so called haplotype tagged SNPs (htSNPs) are indicated by the word Yes. For reference purposes, an imputed Chimp haplotype was determined by aligning the human locus of interest to the Chimp genome using the Chimp BLAT Search program at the UCSC Genome Bioinformatics Site. TXNRD1 Haplotype Frequencies. The software program PHASE was used to define haplotypes for the TXNRD1 locus. Haplotype frequencies for each ethnic population, SNP locations, RS#, and htSNP data are provided. [file 1471-2156-7-56-S12.pdf]

Thioredoxin Reductase 1 (TXNRD1) Haplotypes

Prettybase	104	2498	2791	2972	4658	29281	29309	31689	47707	64088	64440	66518	66537	ALL (n=204)		AA (n=48)		CA (n=62)		PR (n=48)		HI (n=46)	
SNP Location	-27130	-24736	-24443	-24262	-22576	IVS2 +57	IVS2 +85	L55L	IVS10 +158	IVS12 +8907	+111	+2189	+2208	n	f	n	f	n	f	n	f	n	f
RS#		rs4077561	rs10735393	rs1128446		rs5018287		rs4964287	rs10778322	rs11112003	rs11611385	rs10047589	rs17808524										
htSNPs	yes	yes	yes	yes	yes	yes					yes	yes	yes										
Chimp	G	T	T	C	C	A	T	C	C	C	C	C	G										
1	G	C	T	C	C	G	T	C	C	C	C	C	C	70	0.343	14	0.29	12	0.19	31	0.65	13	0.28
2	G	T	T	G	C	A	T	T	T	T	C	T	G	31	0.152	9	0.19	11	0.18	3	0.06	8	0.17
3	T	C	T	C	C	G	T	C	C	C	C	C	C	22	0.108	2	0.04	13	0.21	1	0.02	6	0.13
4	G	T	T	C	C	A	T	T	T	C	T	T	G	15	0.074	1	0.02	6	0.10	3	0.06	5	0.11
5	G	T	G	C	C	A	A	C	C	C	C	C	G	11	0.054			7	0.11			4	0.09
6	G	T	G	C	C	A	T	C	C	C	C	C	G	8	0.039			2	0.03	5	0.10	1	0.02
7	G	T	G	C	G	A	T	C	C	C	C	C	G	7	0.034	4	0.08	2	0.03	1	0.02		
8	G	T	T	C	C	A	T	T	T	T	C	T	G	5	0.025	4	0.08					1	0.02
9	G	C	T	C	C	A	T	C	C	C	C	C	G	5	0.025	2	0.04	2	0.03			1	0.02
10	G	T	T	G	C	A	T	T	C	T	C	T	G	4	0.020	1	0.02	1	0.02			2	0.04
11	G	T	T	C	C	A	T	T	T	C	C	T	G	3	0.015	2	0.04					1	0.02
12	G	T	T	C	C	G	T	C	C	C	C	C	C	3	0.015	1	0.02			2	0.04		
13	G	T	G	C	G	G	T	C	C	C	C	C	C	3	0.015	3	0.06						
14	G	C	T	G	C	A	T	T	T	T	C	T	G	3	0.015			3	0.05				
15	G	C	T	G	C	A	T	T	T	T	C	C	G	2	0.010	1	0.02					1	0.02
16	G	T	G	C	C	A	T	T	T	C	C	T	G	2	0.010	2	0.04						
17	G	T	G	C	C	G	T	C	C	C	C	C	C	2	0.010			1	0.02			1	0.02
18	G	C	G	C	G	A	T	C	C	C	C	C	G	1	0.005	1	0.02						
19	G	C	G	C	G	A	T	C	C	C	C	T	G	1	0.005	1	0.02						
20	G	T	G	C	C	A	A	C	C	C	C	T	G	1	0.005			1	0.02				
21	G	C	G	C	C	A	A	C	C	C	C	C	G	1	0.005			1	0.02				
22	G	T	G	C	C	A	T	T	T	C	C	T	C	1	0.005							1	0.02
23	G	T	G	C	C	A	T	C	C	C	C	T	G	1	0.005							1	0.02
24	T	C	T	G	C	G	T	C	C	C	C	C	C	1	0.005					1	0.02		
25	G	T	T	C	C	G	T	C	T	C	T	T	G	1	0.005					1	0.02		
											Unique Haplotypes			25		15		13		9		14	
