# Supplementary material for: Polymorphism analysis of six selenoprotein genes: support for a selective sweep at the glutathione peroxidase 1 locus (3p21) in Asian populations
Source: BMC Genet. 2006 Dec 11;7:56. doi: 10.1186/1471-2156-7-56 (PMC1769511; doi:10.1186/1471-2156-7-56)
Supplement: Additional File 13 — Estimates for linkage disequilibrium (LD) and location of major haplotype blocks across 6 selenoprotein loci, stratified by ethnic subpopulation. Pair wise plots (D') across 6 selenoprotein loci based on genotype data obtained from re-sequencing DNA samples from individuals of AA (n = 24), CA (n = 31), HI (n = 23) and PR (n = 24; n = 23 for GPX1)heritage from the SNP500 DNA population. Re-sequenced genes include a) GPX1, b) GPX2, c) GPX3, d) GPX4, e) SEPP1, and f) TXNRD1. SNP identifiers are indicated on the abscissas. Numbers within cells correspond to LD values (D'). The LD color scheme is stratified according to the logarithm of the odds (LOD) score and D': LOD <2 (white for D'<1 and blue for D' = 1) or LOD >2 (shades of pink/red for D'<1 and bright red for D' = 1). Haplotype blocks were created using the algorithm of Gabriel et al, Science 2002 [76]. 95% confidence bounds on D' were generated and each comparison was called "strong LD", "inconclusive" or "strong recombination". A block was created if 95% of informative comparisons were "strong LD". LD Plots For Ethnic Subpopulations. Estimation of linkage disequilibrium (D') and the location of major haplotype blocks across each of the six selenoprotein loci is provided; the data in this file is stratified by ethnic subpopulation. [file 1471-2156-7-56-S13.pdf]

Estimation of linkage disequilibrium and location of major haplotype blocks across six selenoprotein loci, stratified by ethnic subpopulation.

GPX1.AA

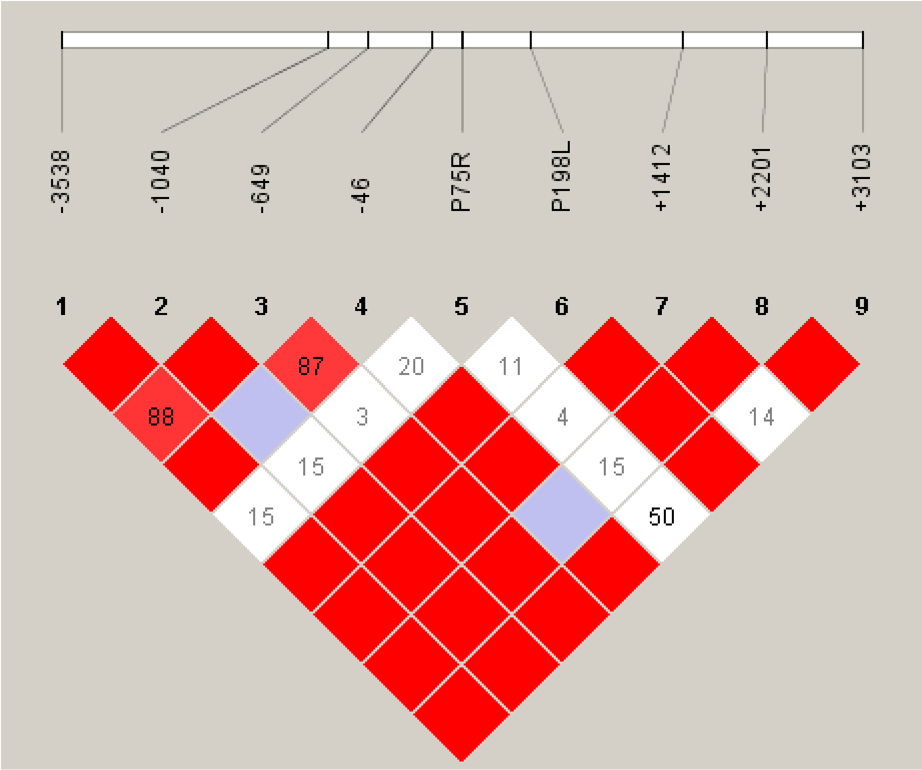

GPX1.CA

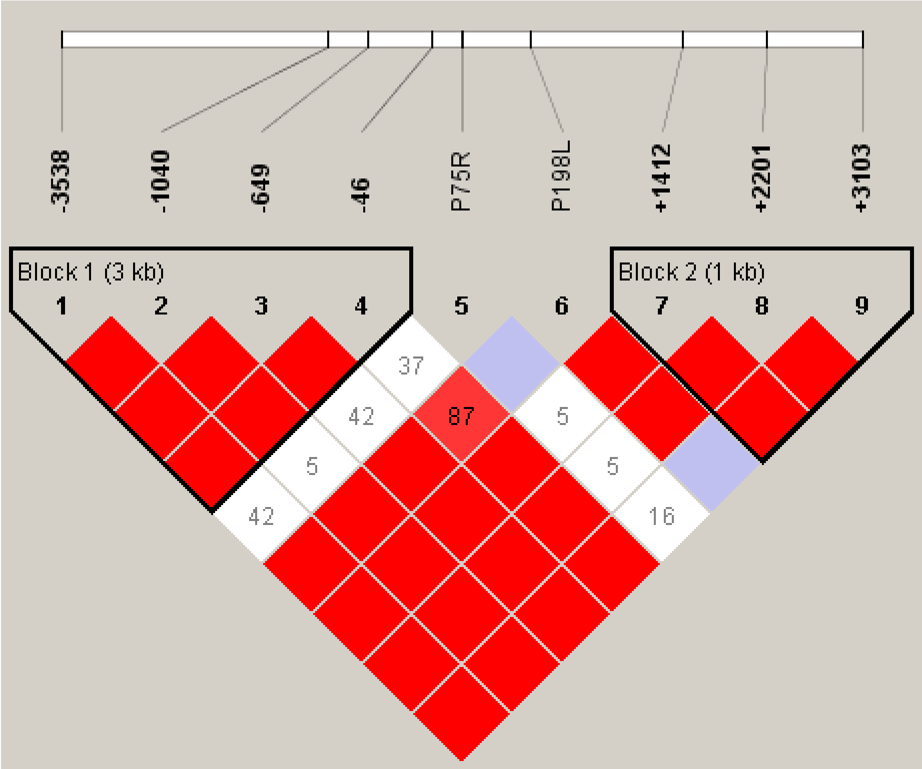

-3538
-1040
-649
-46
P75R
P198L
+1412
+2201
+3103
Block 1 (3 kb)
Block 2 (1 kb)
1
2
3
4
5
6
7
8
9
37
42
87
5
5
5
42
16

GPX1.HI

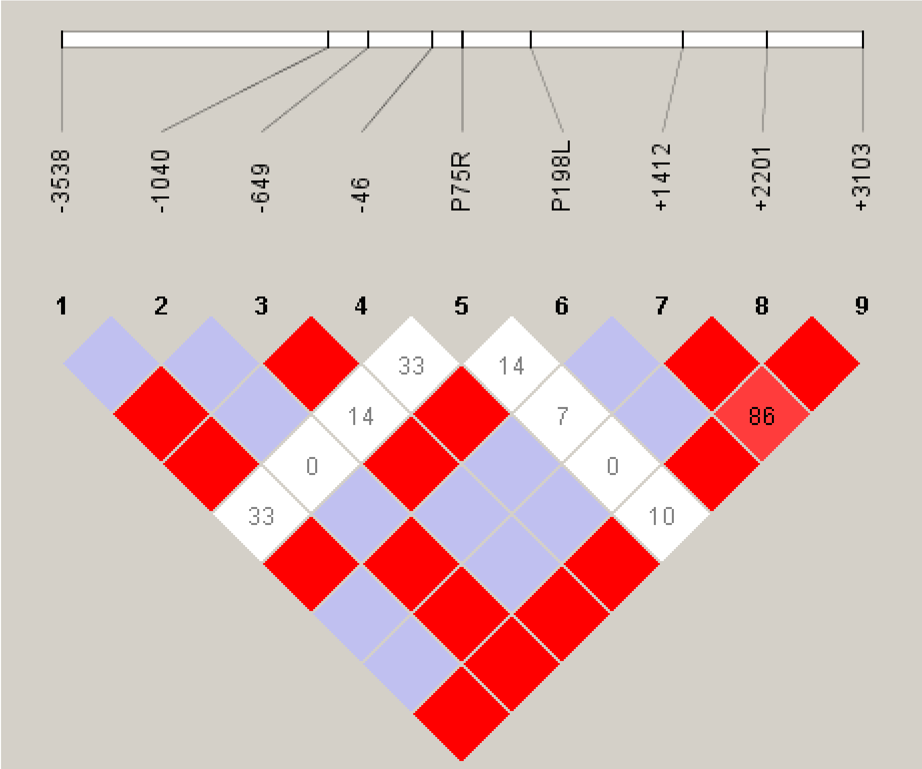
-3538
-1040
-649
-46
P75R
P198L
+1412
+2201
+3103
1
2
3
4
5
6
7
8
9
33
14
14
7
86
0
0
33
10

GPX1.PR

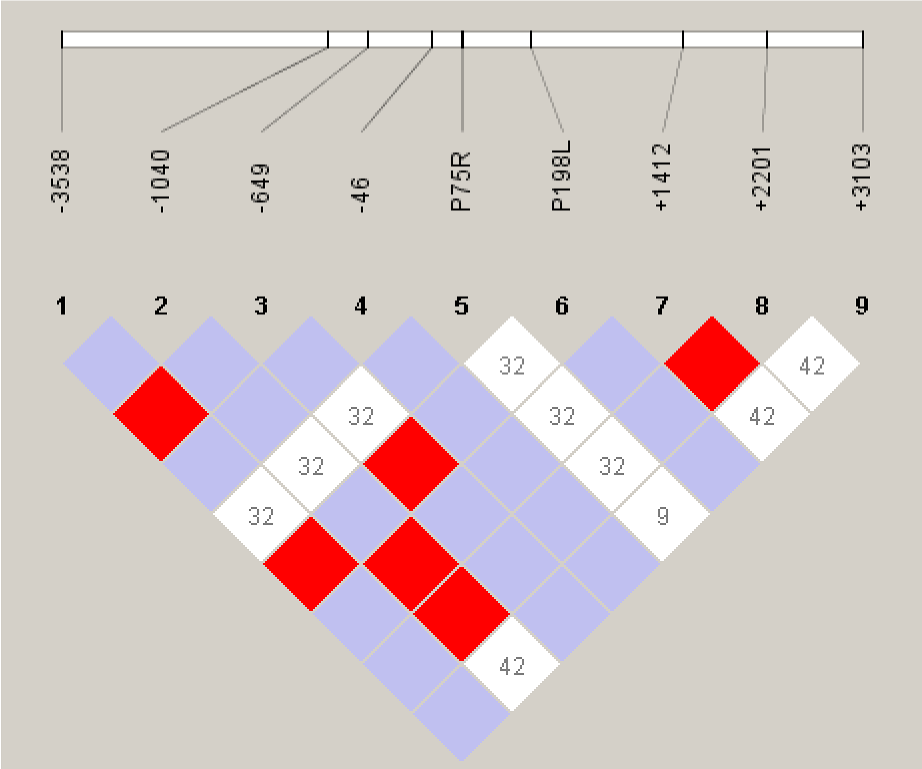

-3538
-1040
-649
-46
P75R
P198L
+1412
+2201
+3103
1
2
3
4
5
6
7
8
9
32
42
32
32
42
32
32
32
9
42

GPX2.AA

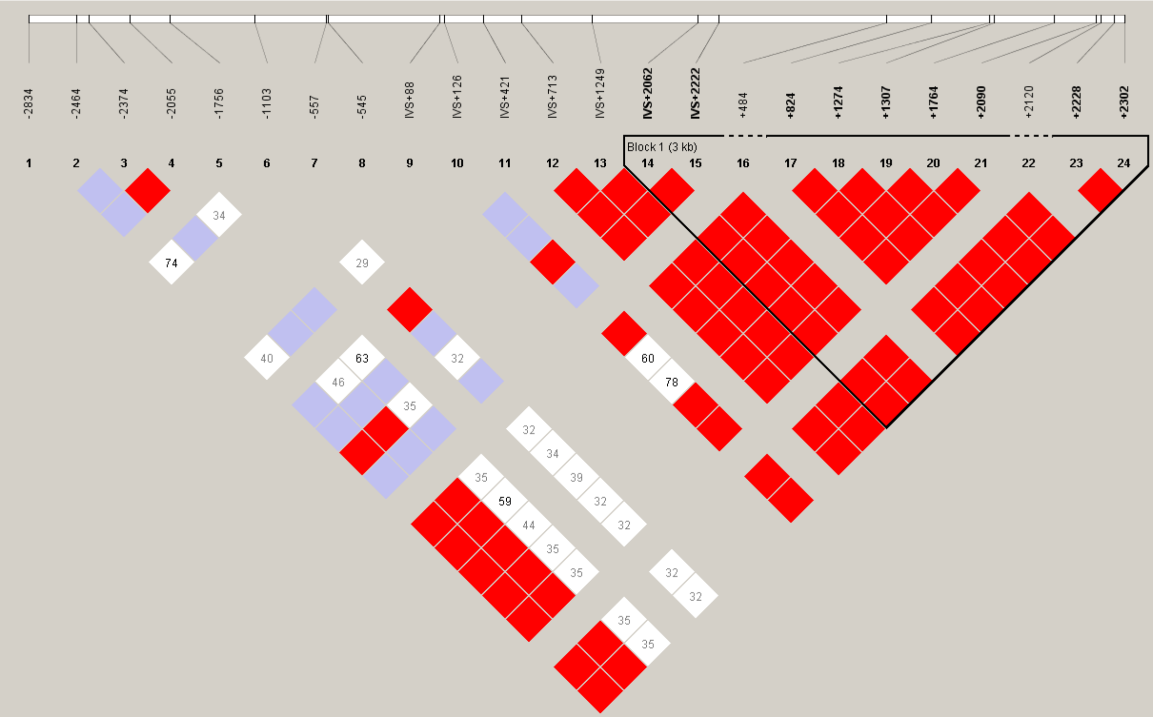

-2834
-2464
-2374
-2055
-1756
-1103
-557
-545
IVS+88
IVS+126
IVS+421
IVS+713
IVS+1249
IVS+2062
IVS+2222
+484
+824
+1274
+1307
+1764
+2090
+2120
+2228
+2302
Block 1 (3 kb)
1
2
3
4
5
6
7
8
9
10
11
12
13
14
15
16
17
18
19
20
21
22
23
24

GPX2.CA

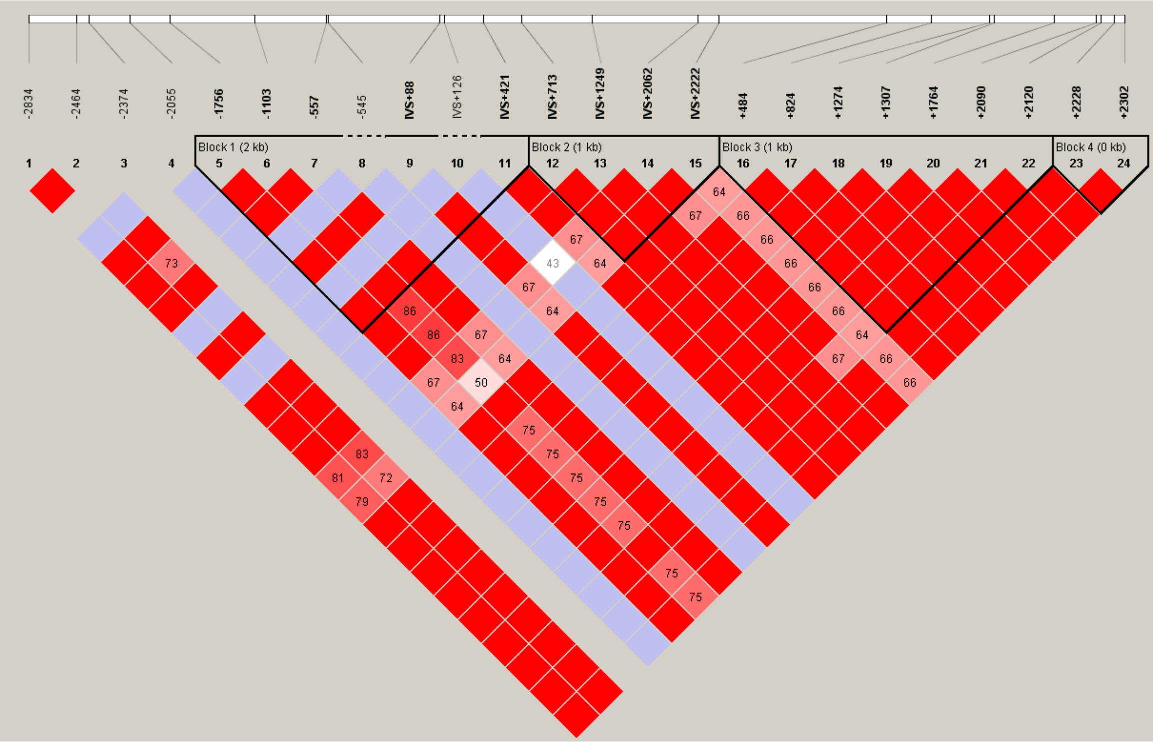
-2834
-2464
-2374
-2055
-1756
-1103
-557
-545
IVS+88
IVS+126
IVS+421
IVS+713
IVS+1249
IVS+2062
IVS+2222
+484
+824
+1274
+1307
+1764
+2090
+2120
+2228
+2302
Block 1 (2 kb)
Block 2 (1 kb)
Block 3 (1 kb)
Block 4 (0 kb)
1 2 3 4 5 6 7 8 9 10 11 12 13 14 15 16 17 18 19 20 21 22 23 24

GPX2.HI

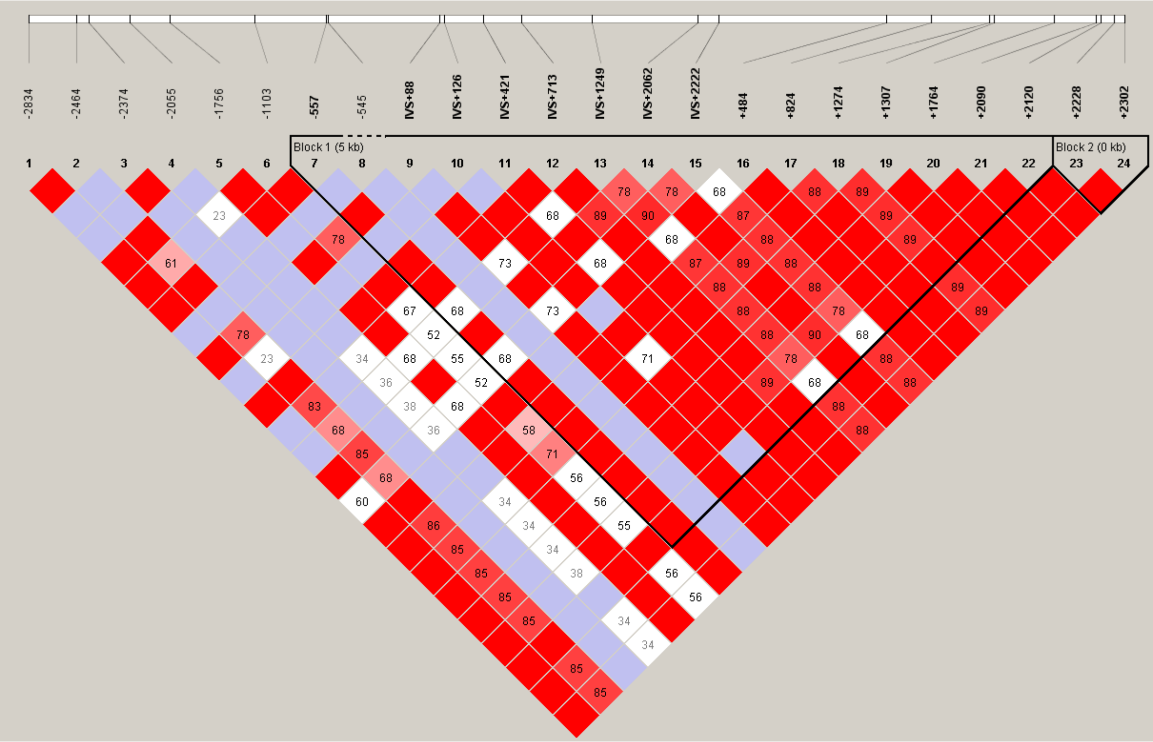
-2834
-2464
-2374
-2055
-1756
-1103
-557
-545
IVS+88
IVS+126
IVS+421
IVS+713
IVS+1249
IVS+2062
IVS+2222
+484
+824
+1274
+1307
+1764
+2090
+2120
+2228
+2302
Block 1 (5 kb)
Block 2 (0 kb)
1 2 3 4 5 6 7 8 9 10 11 12 13 14 15 16 17 18 19 20 21 22 23 24

GPX2.PR

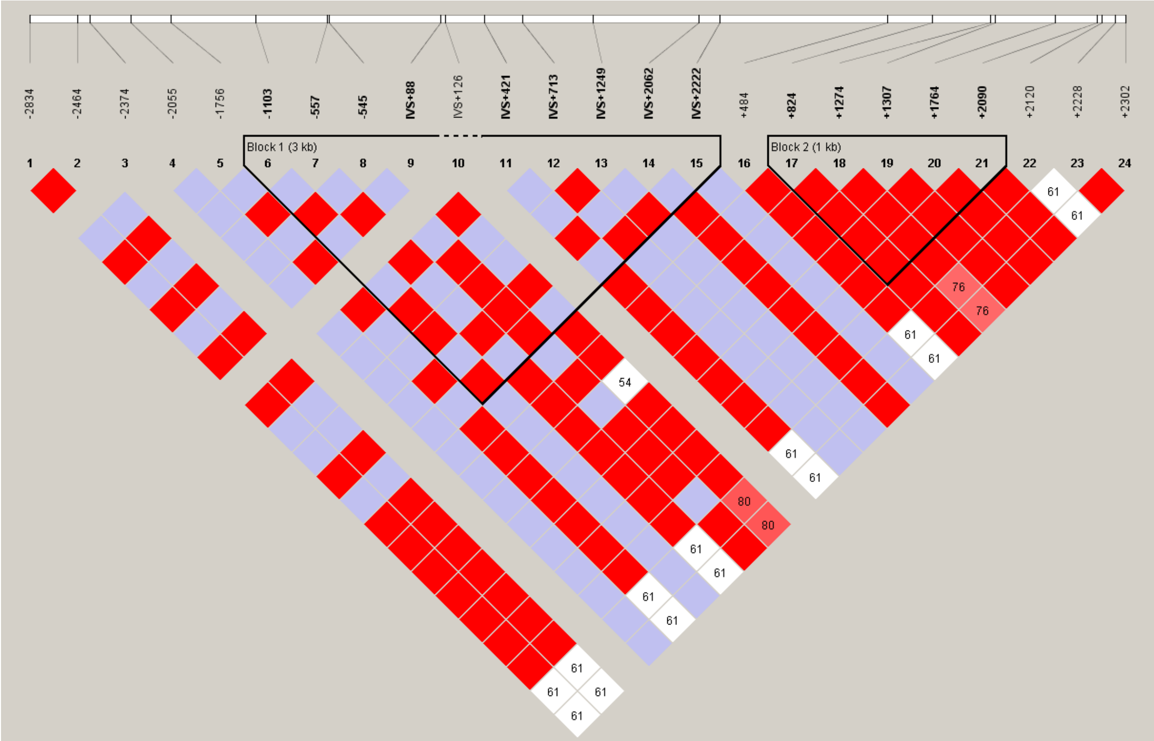

-2834
-2464
-2374
-2055
-1756
-1103
-557
-545
IVS+88
IVS+126
IVS+421
IVS+713
IVS+1249
IVS+2062
IVS+2222
+484
+824
+1274
+1307
+1764
+2090
+2120
+2228
+2302
Block 1 (3 kb)
Block 2 (1 kb)
1
2
3
4
5
6
7
8
9
10
11
12
13
14
15
16
17
18
19
20
21
22
23
24
61
61
76
76
61
61
54
61
61
80
80
61
61
61
61
61
61
61
61

GPX3.AA

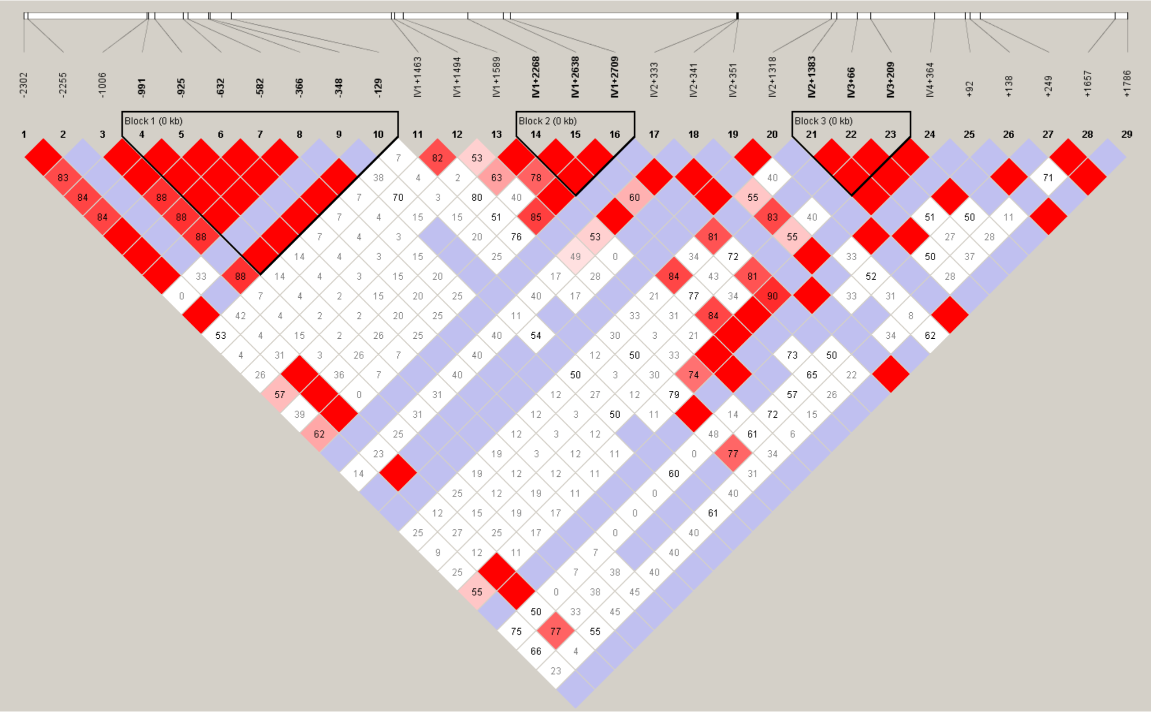

-2302
-2255
-1006
-991
-925
-632
-582
-366
-348
-129
IV1+1463
IV1+1494
IV1+1589
IV1+2268
IV1+2638
IV1+2709
IV2+333
IV2+341
IV2+351
IV2+1318
IV2+1383
IV3+66
IV3+209
IV4+364
+92
+138
+249
+1657
+1786
Block 1 (0 kb)
Block 2 (0 kb)
Block 3 (0 kb)
1 2 3 4 5 6 7 8 9 10 11 12 13 14 15 16 17 18 19 20 21 22 23 24 25 26 27 28 29

GPX3.CA

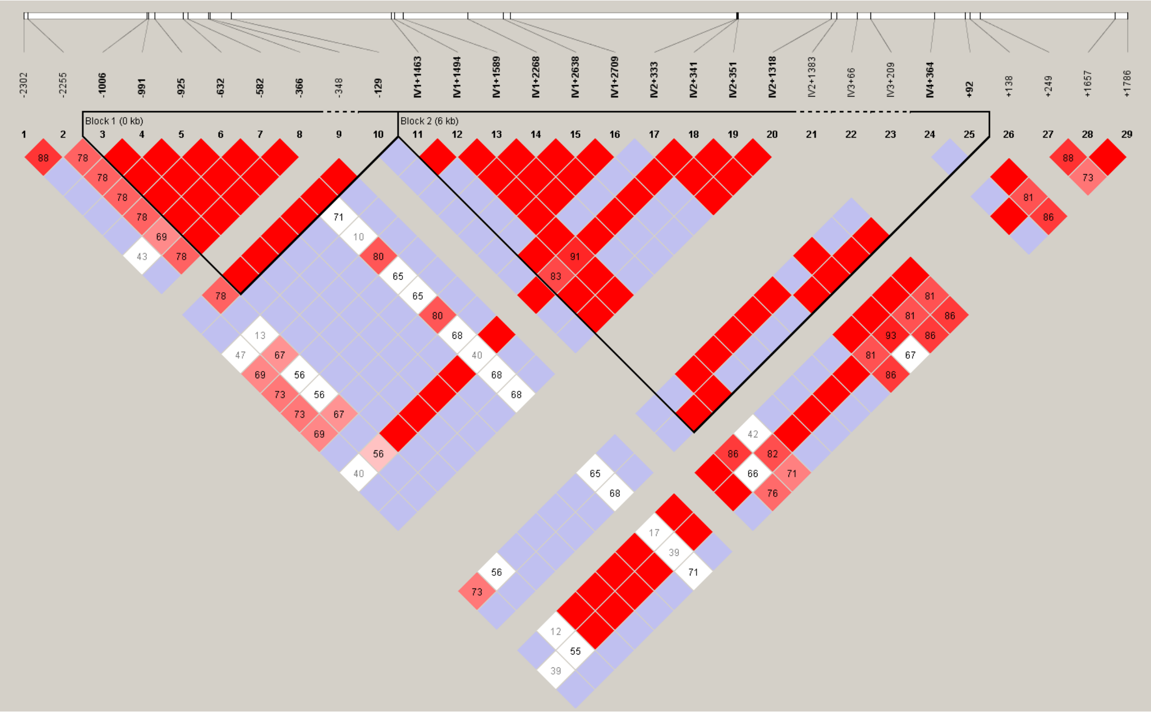

-2302
-2255
-1006
-991
-925
-632
-582
-366
-348
-129
IV1+1463
IV1+1494
IV1+1589
IV1+2268
IV1+2638
IV1+2709
IV2+333
IV2+341
IV2+351
IV2+1318
IV2+1383
IV3+66
IV3+209
IV4+364
+92
+138
+249
+1657
+1786
Block 1 (0 kb)
Block 2 (6 kb)
1 2 3 4 5 6 7 8 9 10 11 12 13 14 15 16 17 18 19 20 21 22 23 24 25 26 27 28 29

GPX3.HI

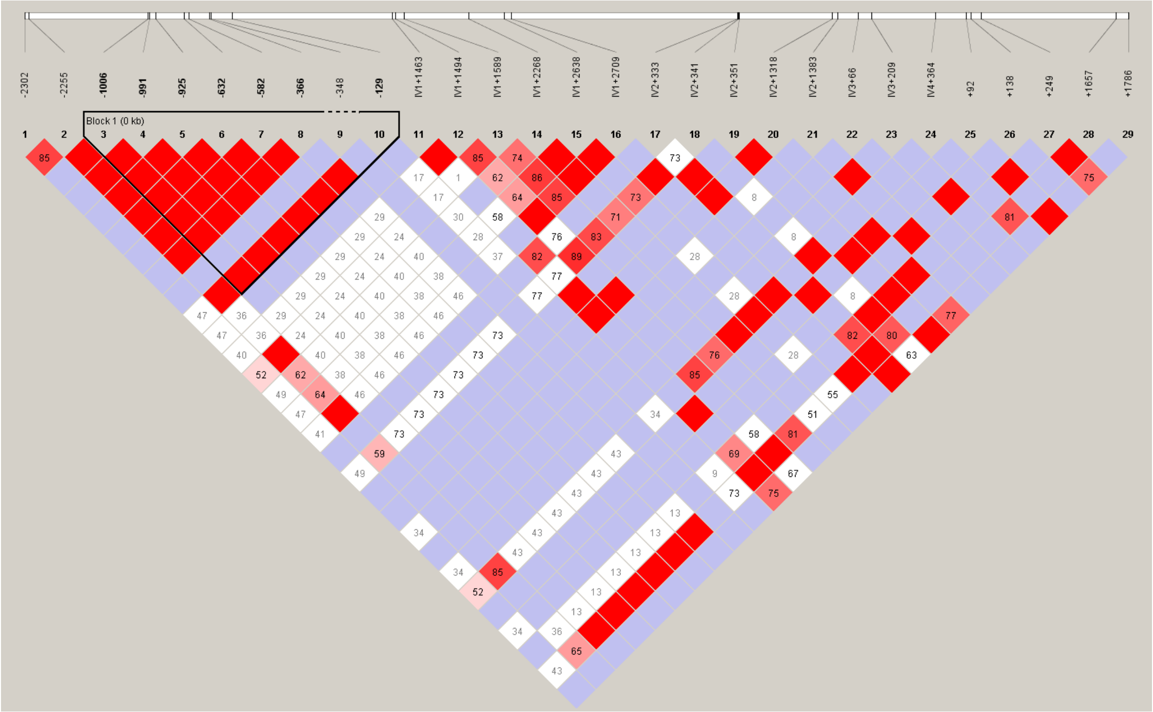
-2302
-2255
-1006
-991
-925
-632
-582
-366
-348
-129
IV1+1463
IV1+1494
IV1+1589
IV1+2268
IV1+2638
IV1+2709
IV2+333
IV2+341
IV2+351
IV2+1318
IV2+1383
IV3+66
IV3+209
IV4+364
+92
+138
+249
+1657
+1786
Block 1 (0 kb)
1 2 3 4 5 6 7 8 9 10 11 12 13 14 15 16 17 18 19 20 21 22 23 24 25 26 27 28 29

GPX3.PR

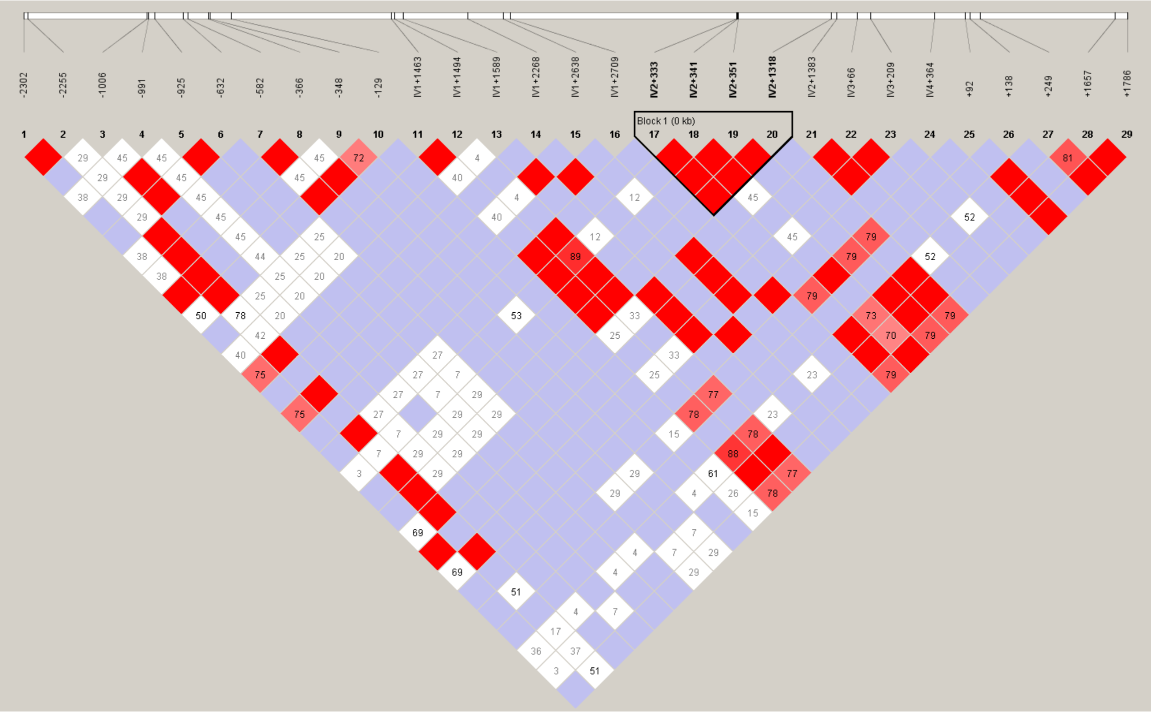
-2302
-2255
-1006
-991
-925
-632
-582
-366
-348
-129
IV1+1463
IV1+1494
IV1+1589
IV1+2268
IV1+2638
IV1+2709
IV2+333
IV2+341
IV2+351
IV2+1318
IV2+1383
IV3+66
IV3+209
IV4+364
+92
+138
+249
+1657
+1786
Block 1 (0 kb)
1 2 3 4 5 6 7 8 9 10 11 12 13 14 15 16 17 18 19 20 21 22 23 24 25 26 27 28 29

GPX4.AA

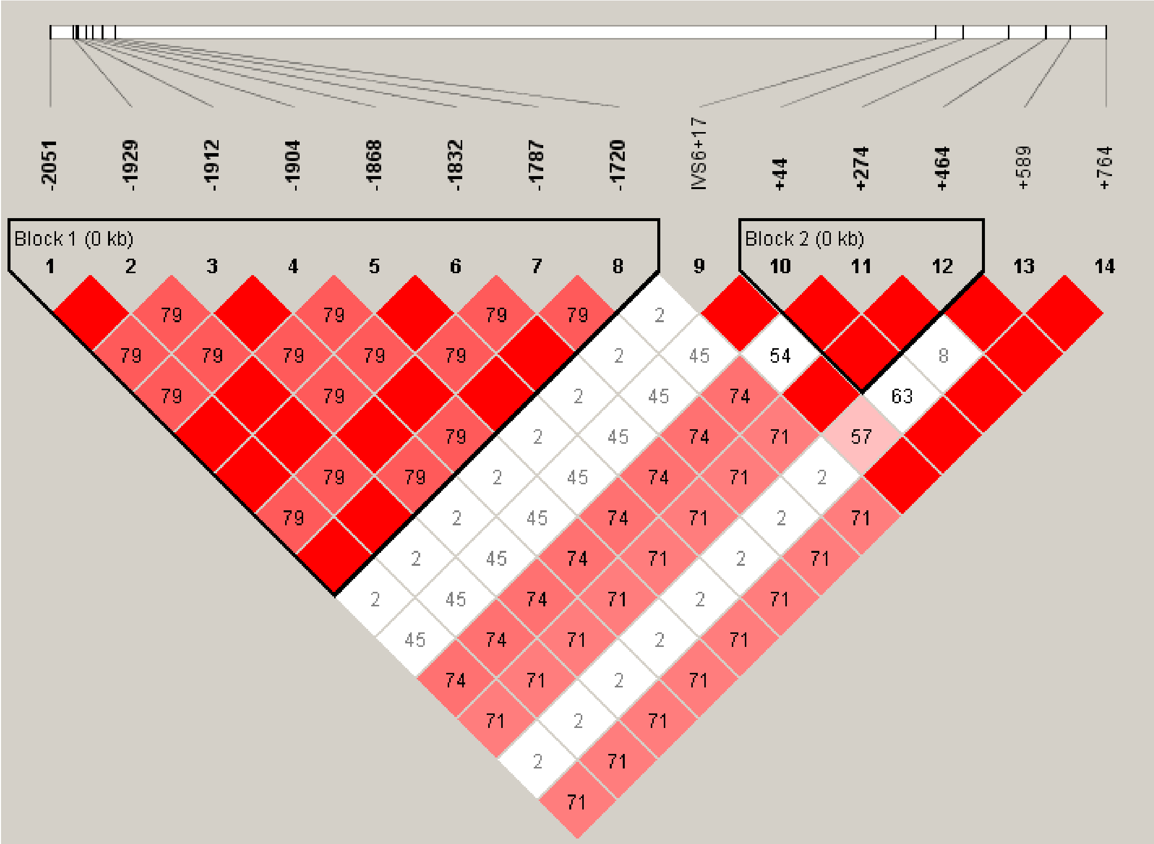
-2051
-1929
-1912
-1904
-1868
-1832
-1787
-1720
IVS6+17
+44
+274
+464
+589
+764
Block 1 (0 kb)
Block 2 (0 kb)
1 2 3 4 5 6 7 8 9 10 11 12 13 14

GPX4.CA

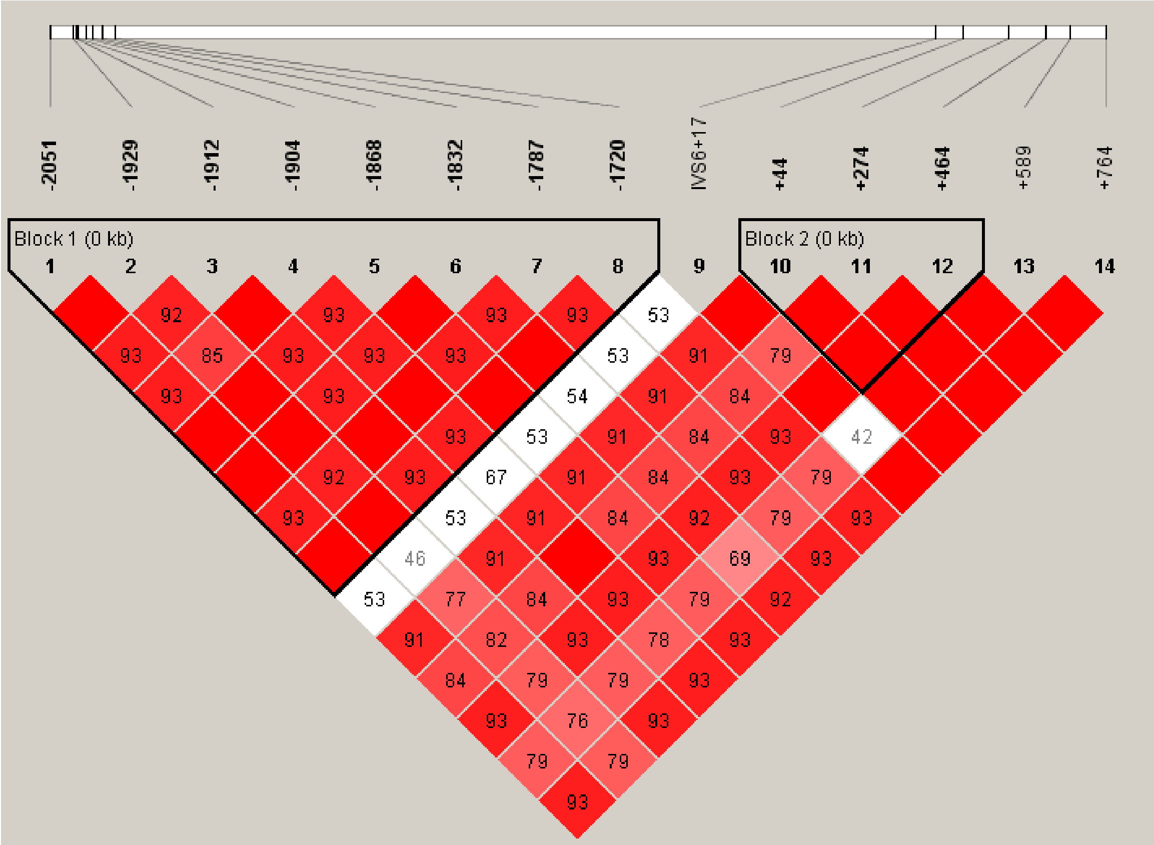
-2051
-1929
-1912
-1904
-1868
-1832
-1787
-1720
IVS6+17
+44
+274
+464
+589
+764
Block 1 (0 kb)
Block 2 (0 kb)
1
2
3
4
5
6
7
8
9
10
11
12
13
14

GPX4.HI

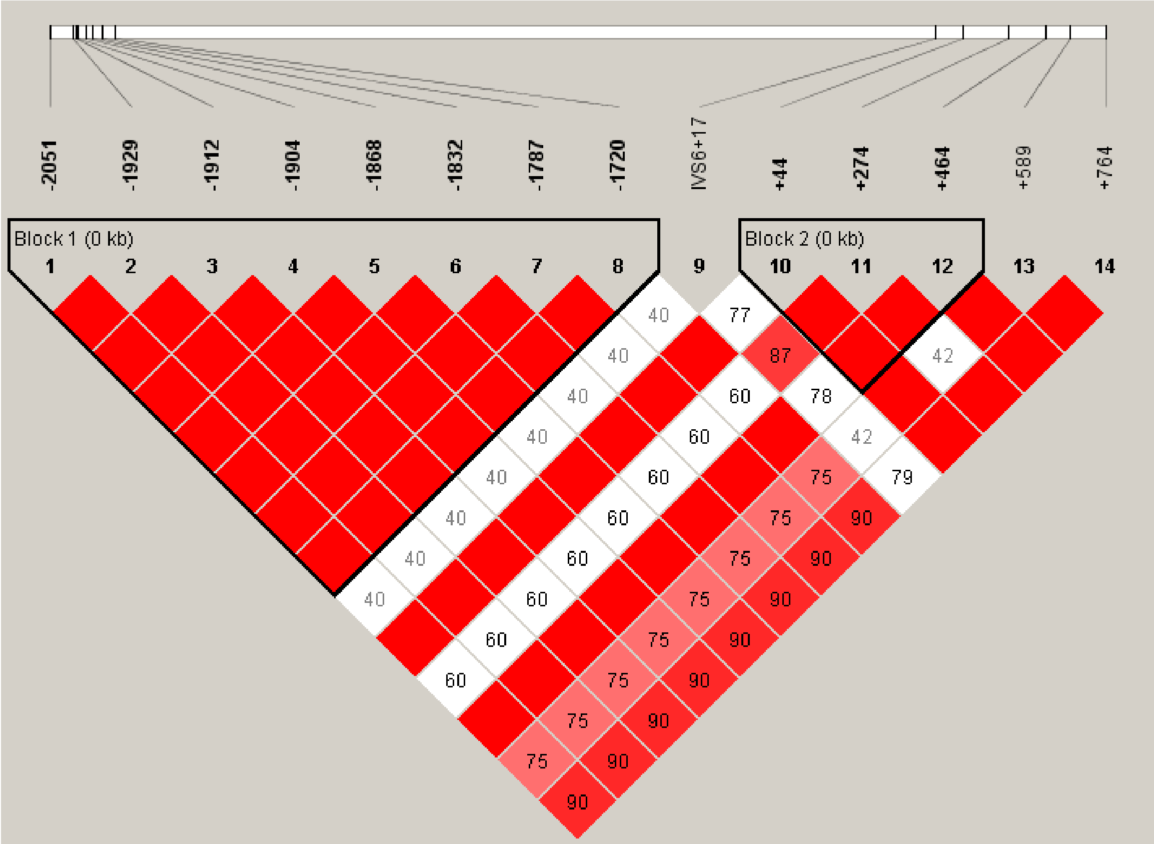
-2051
-1929
-1912
-1904
-1868
-1832
-1787
-1720
IVS6+17
+44
+274
+464
+589
+764
Block 1 (0 kb)
Block 2 (0 kb)
1 2 3 4 5 6 7 8 9 10 11 12 13 14

GPX4.PR

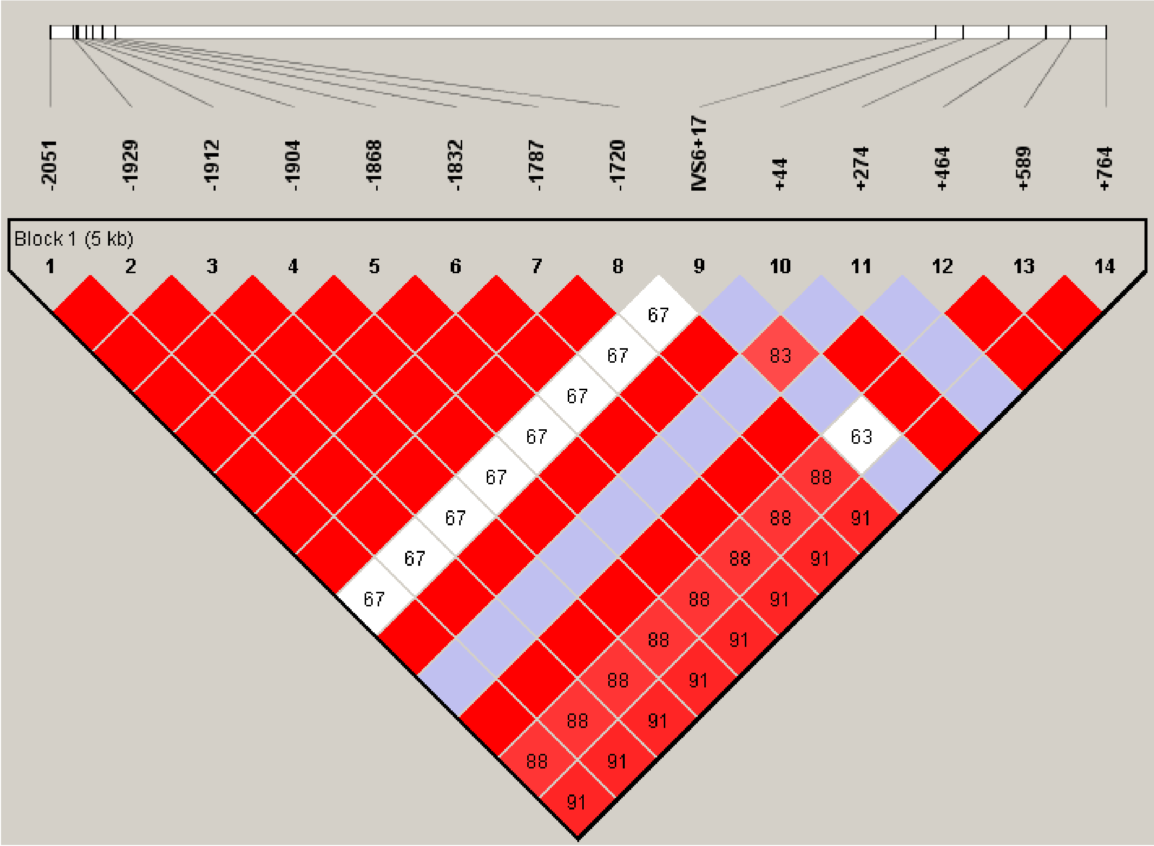

-2051
-1929
-1912
-1904
-1868
-1832
-1787
-1720
IVS6+17
+44
+274
+464
+589
+764
Block 1 (5 kb)
1 2 3 4 5 6 7 8 9 10 11 12 13 14
67
67
67
67
67
67
67
67
83
63
88
88
88
88
88
88
88
91
91
91
91
91
91
91

SEPP1.AA

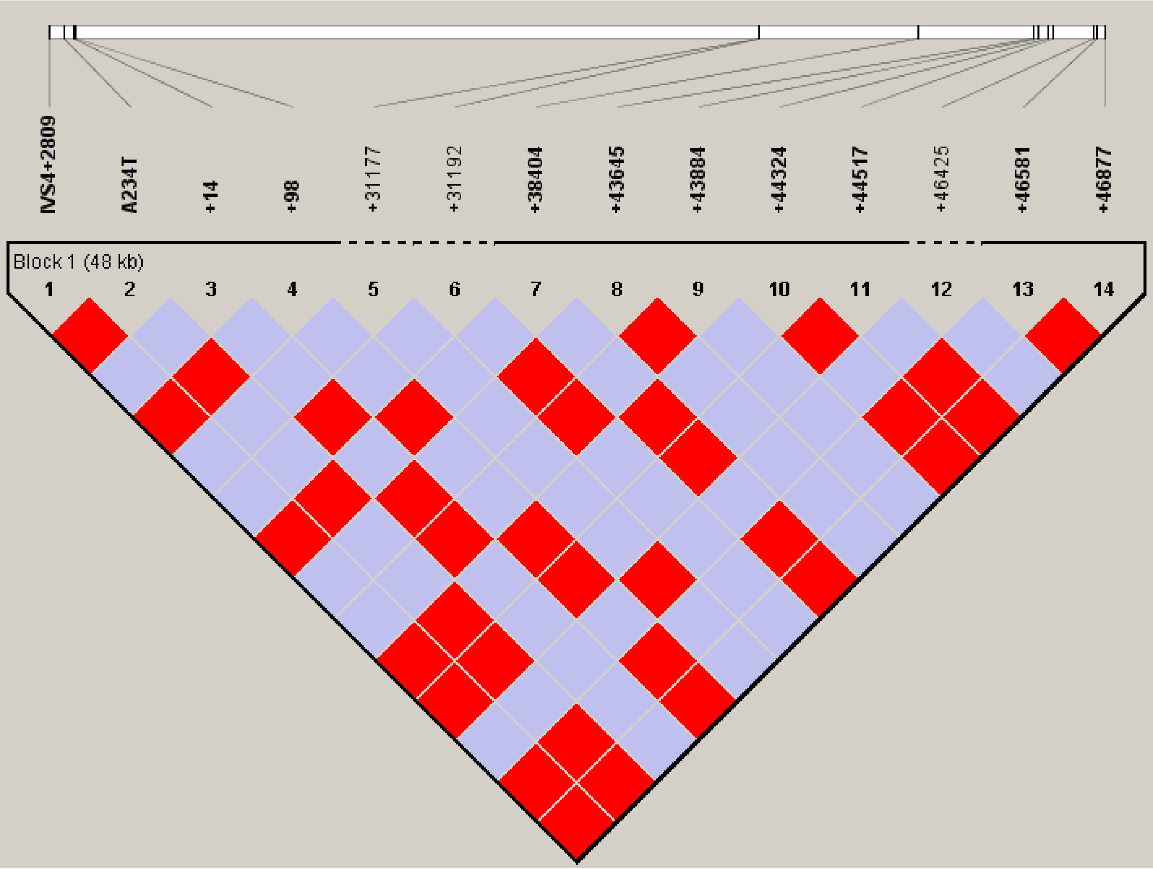

IVS4+2809
A234T
+14
+98
+31177
+31192
+38404
+43645
+43884
+44324
+44517
+46425
+46581
+46877
Block 1 (48 kb)
1
2
3
4
5
6
7
8
9
10
11
12
13
14

SEPP1.CA

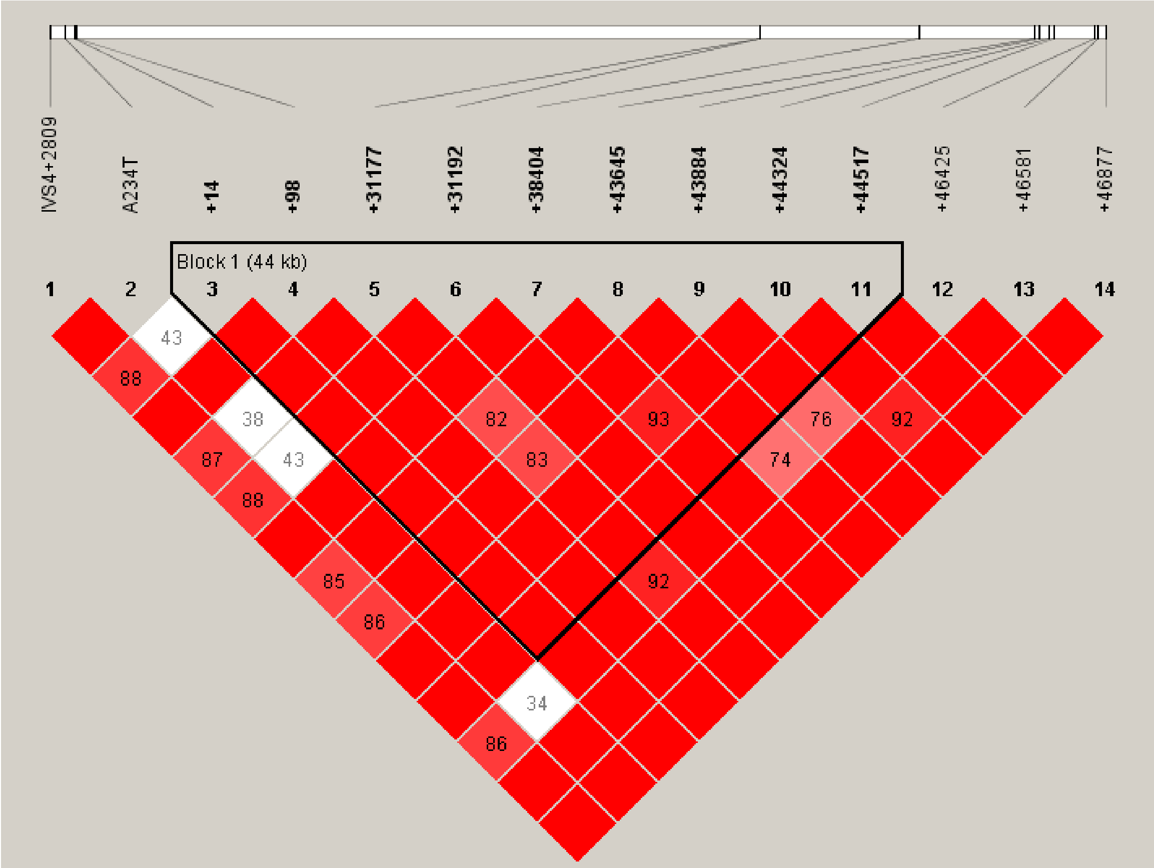
IVS4+2809
A234T
+14
+98
+31177
+31192
+38404
+43645
+43884
+44324
+44517
+46425
+46581
+46877
Block 1 (44 kb)
1
2
3
4
5
6
7
8
9
10
11
12
13
14
43
88
38
87
43
88
82
83
93
76
74
92
85
86
92
34
86

SEPP1.HI

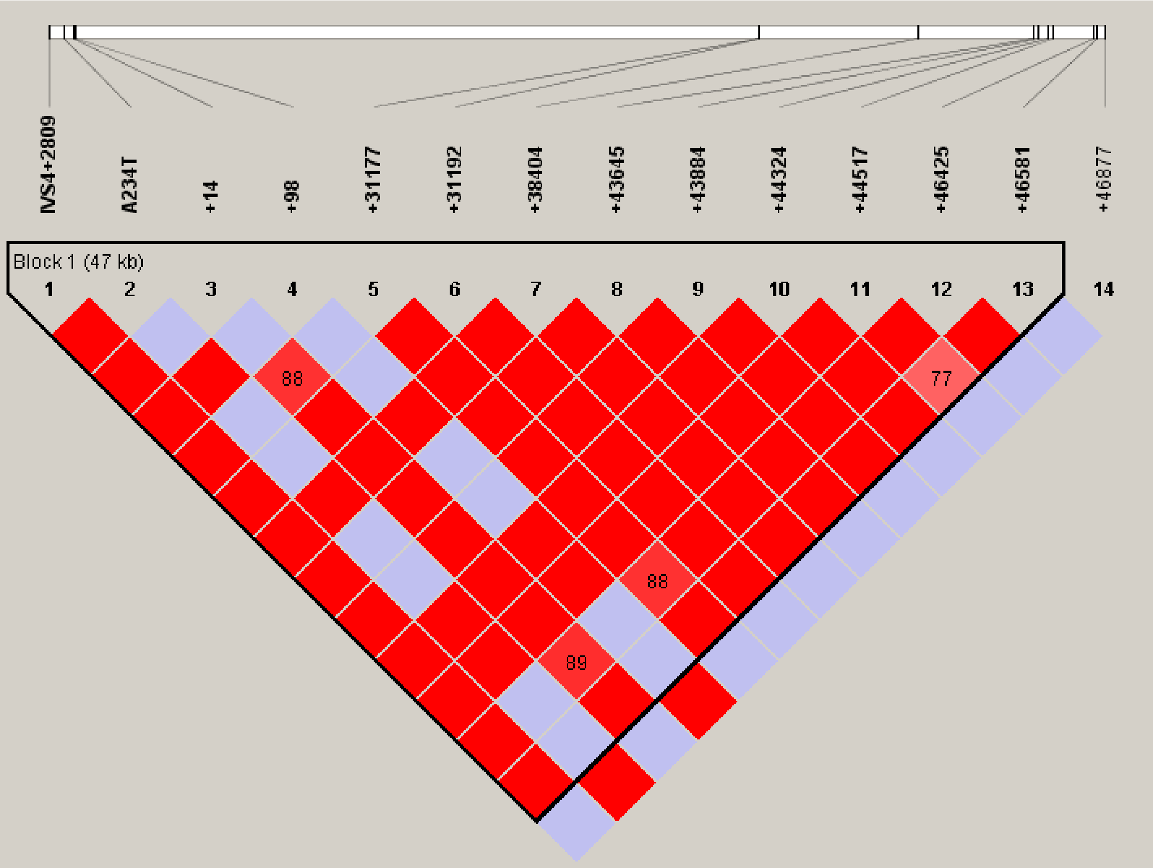
IVS4+2809
A234T
+14
+98
+31177
+31192
+38404
+43645
+43884
+44324
+44517
+46425
+46581
+46877
Block 1 (47 kb)
1
2
3
4
5
6
7
8
9
10
11
12
13
14
88
77
88
89

SEPP1.PR

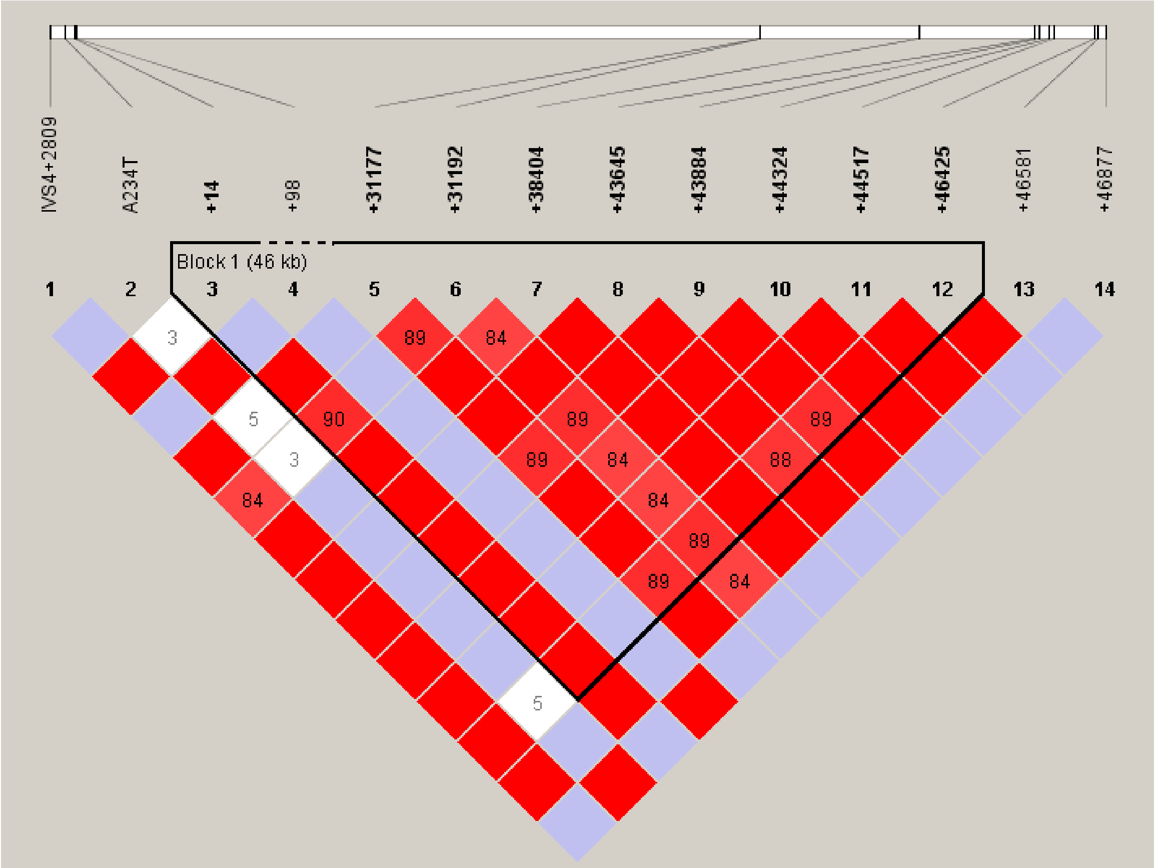
IVS4+2809
A234T
+14
+98
+31177
+31192
+38404
+43645
+43884
+44324
+44517
+46425
+46581
+46877
Block 1 (46 kb)
1
2
3
4
5
6
7
8
9
10
11
12
13
14
3
89
84
5
90
89
89
3
89
84
88
84
84
89
89
84
5

TXNRD1.AA

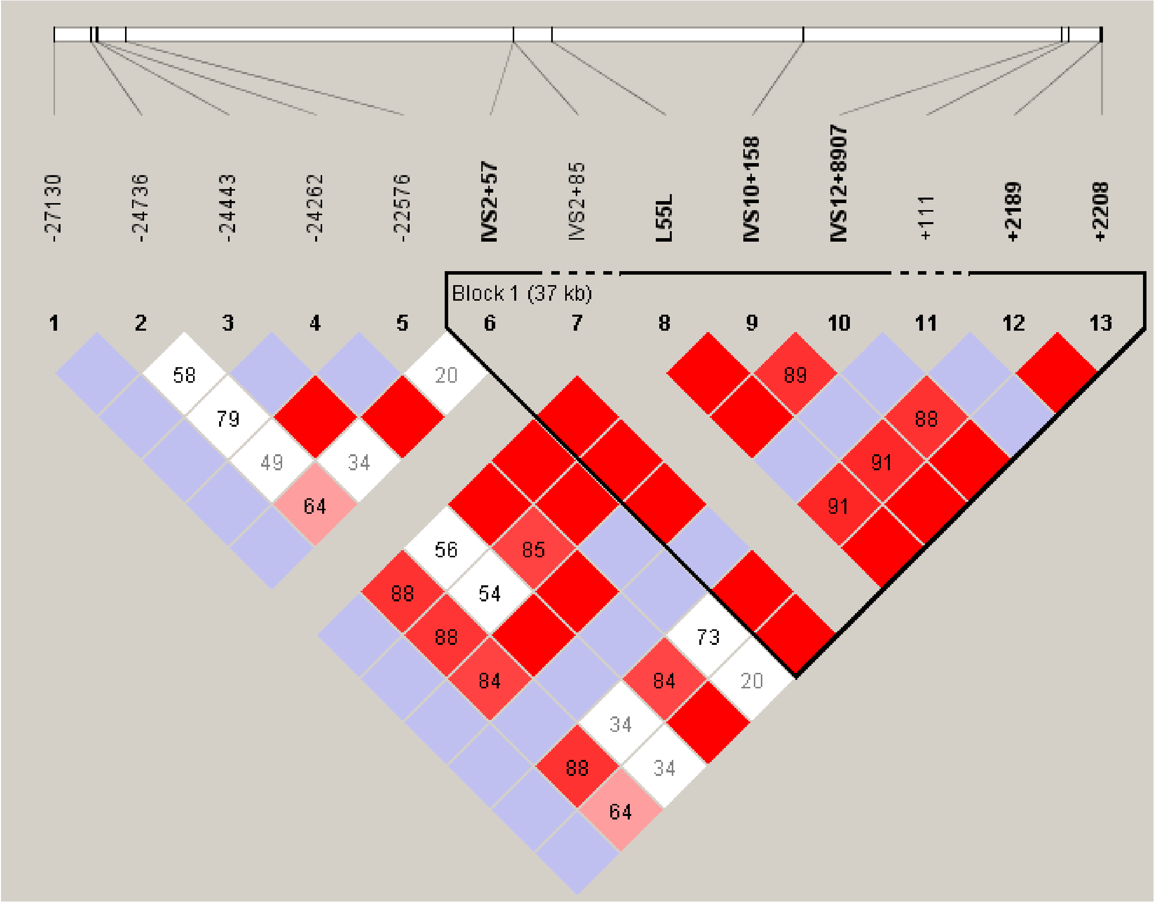

-27130
-24736
-24443
-24262
-22576
IVS2+57
IVS2+85
L55L
IVS10+158
IVS12+8907
+111
+2189
+2208
Block 1 (37 kb)
1
2
3
4
5
6
7
8
9
10
11
12
13
58
20
89
79
88
49
34
91
64
91
56
85
88
54
88
73
84
84
20
34
88
34
64

TXNRD1.CA

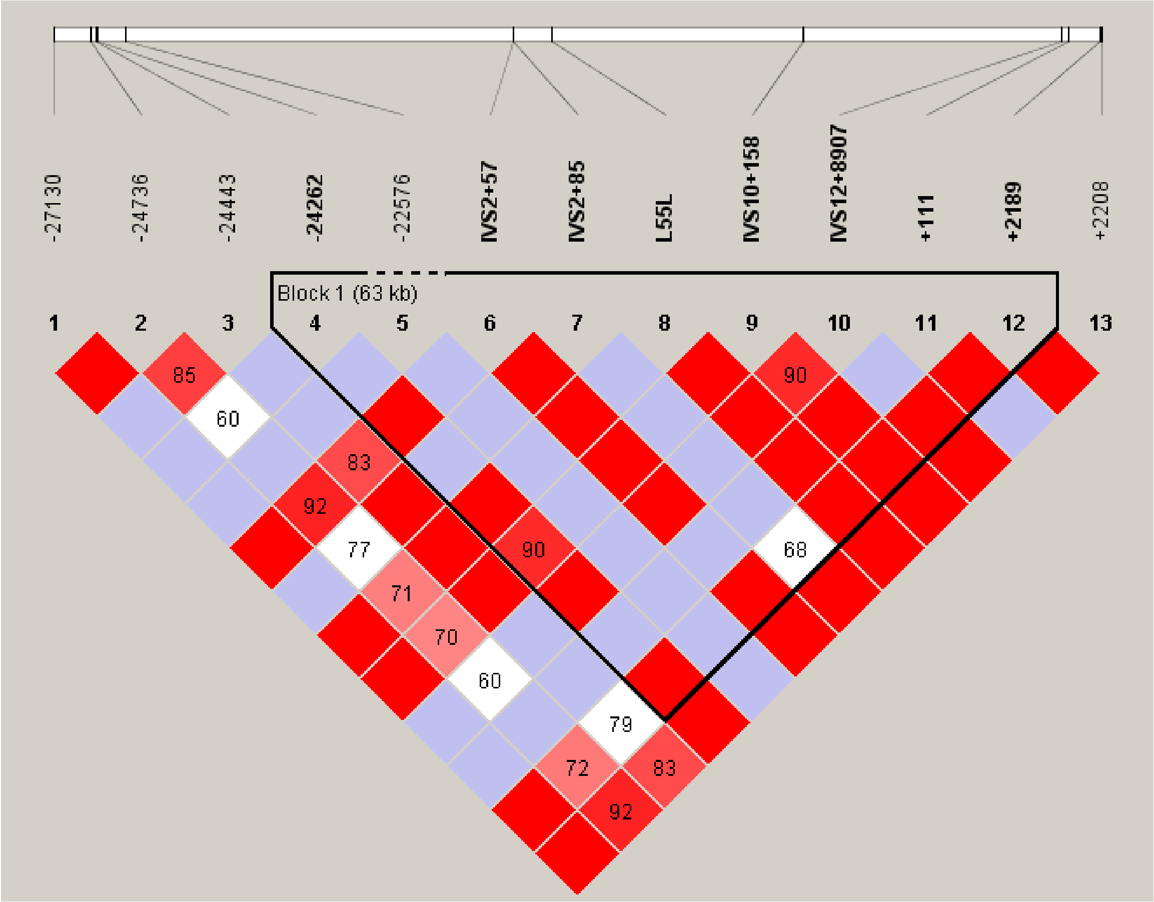

-27130
-24736
-24443
-24262
-22576
IVS2+57
IVS2+85
L55L
IVS10+158
IVS12+8907
+111
+2189
+2208
Block 1 (63 kb)
1
2
3
4
5
6
7
8
9
10
11
12
13
85
60
83
92
77
90
68
90
71
70
60
79
72
83
92

TXNRD1.HI

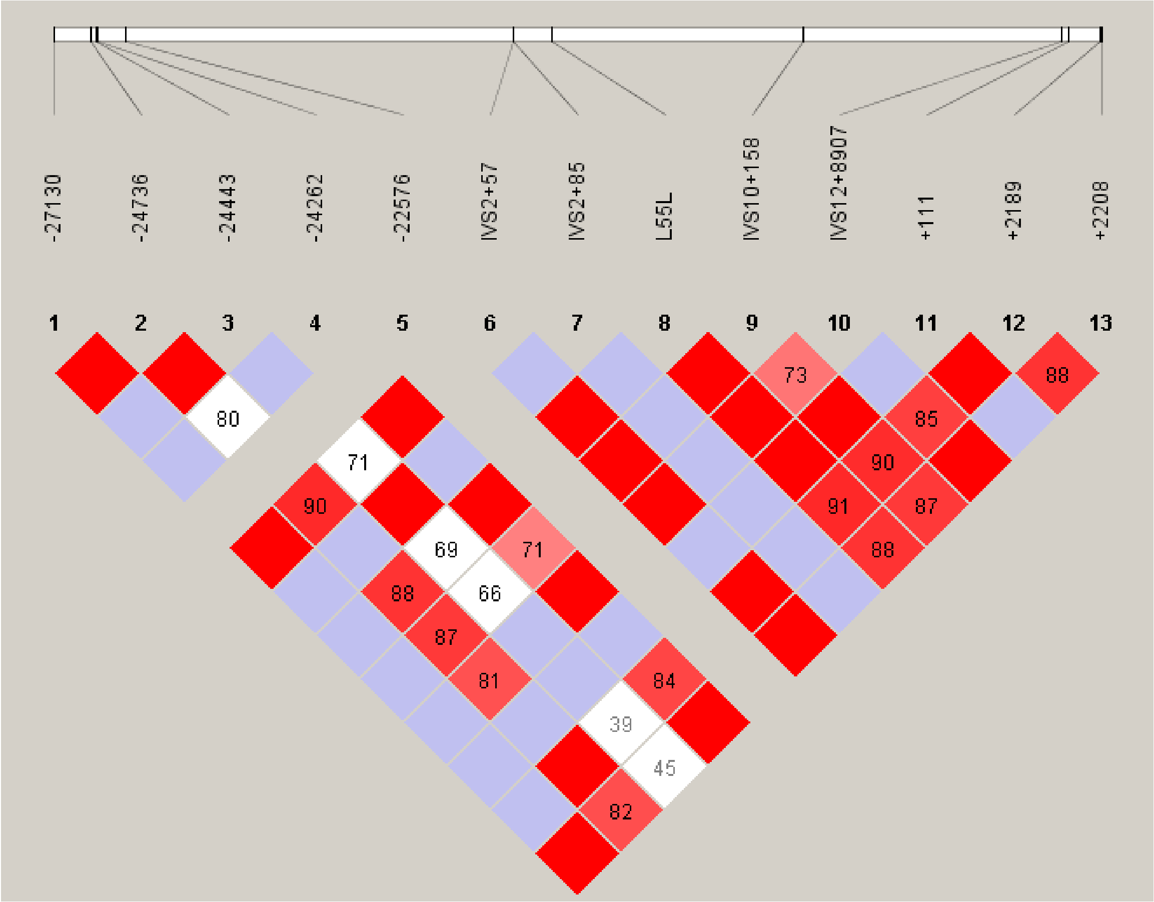

-27130
-24736
-24443
-24262
-22576
IVS2+57
IVS2+85
L55L
IVS10+158
IVS12+8907
+111
+2189
+2208
1
2
3
4
5
6
7
8
9
10
11
12
13
80
71
90
69
71
88
66
87
81
84
39
45
82
73
88
85
90
91
87
88

TXNRD1.PR

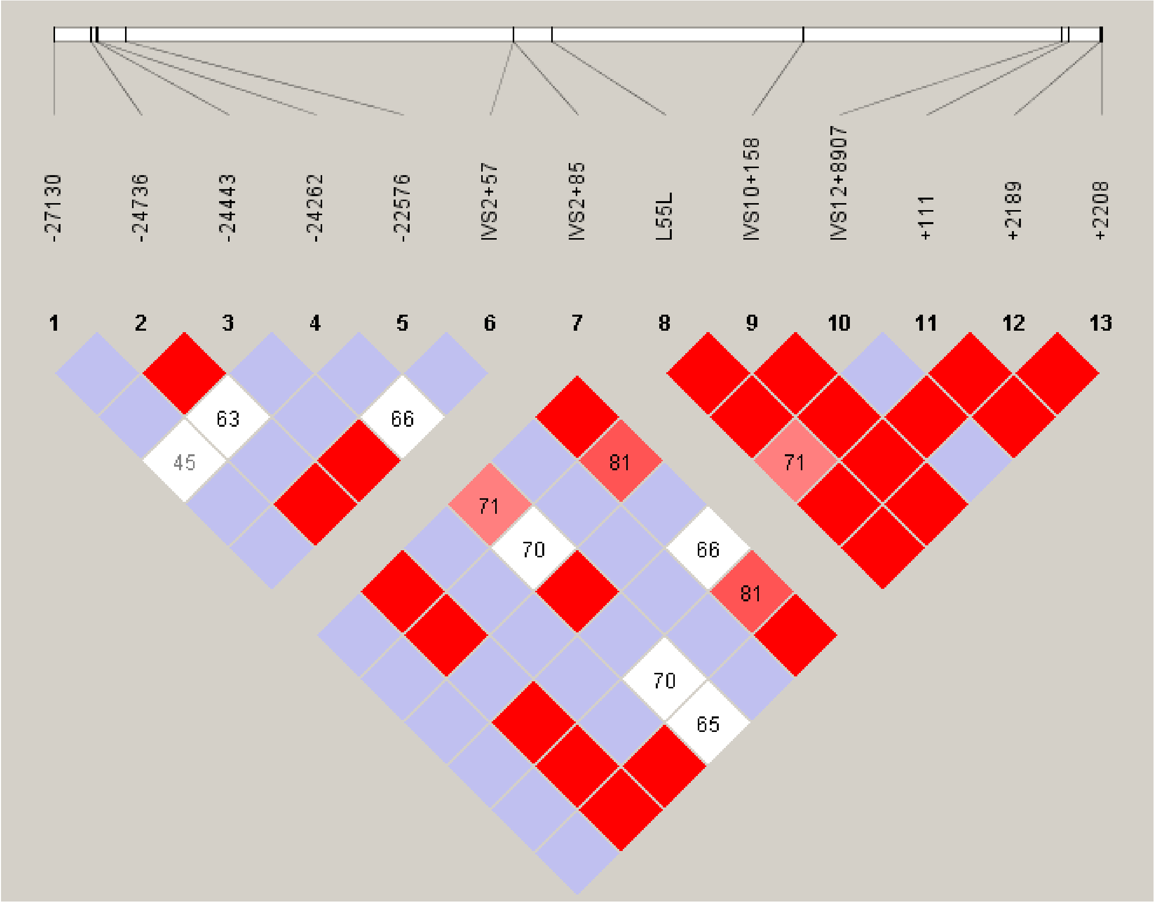

-27130
-24736
-24443
-24262
-22576
IVS2+57
IVS2+85
L55L
IVS10+158
IVS12+8907
+111
+2189
+2208
1
2
3
4
5
6
7
8
9
10
11
12
13
63
66
45
81
71
71
70
66
81
70
65
